# Dual Targeting of MDM4 and FTH1 by MMRi71 for Induced Protein Degradation and p53-Independent Apoptosis in Leukemia Cells

**DOI:** 10.3390/molecules27227665

**Published:** 2022-11-08

**Authors:** Rati Lama, Samuel L. Galster, Chao Xu, Luke W. Davison, Sherry R. Chemler, Xinjiang Wang

**Affiliations:** 1 Department of Pharmacology and Therapeutics, Roswell Park Comprehensive Cancer Center, Buffalo, NY 14263, USA; 2 Department of Chemistry, University at Buffalo, State University of New York, Buffalo, NY 14260, USA

**Keywords:** MMRi, quinolinol, MDM4, E3 ligase, p53, ubiquitination, FTH1, apoptosis, leukemia

## Abstract

MDM2 and MDM4 are cancer drug targets validated in multiple models for p53-based cancer therapies. The RING domains of MDM2 and non-p53-binder MDM2 splice isoforms form RING domain heterodimer polyubiquitin E3 ligases with MDM4, which regulate p53 stability in vivo and promote tumorigenesis independent of p53. Despite the importance of the MDM2 RING domain in p53 regulation and cancer development, small molecule inhibitors targeting the E3 ligase activity of MDM2-MDM4 are poorly explored. Here, we describe the synthesis and characterization of quinolinol derivatives for the identification of analogs that are capable of targeting the MDM2-MDM4 heterodimer E3 ligase and inducing apoptosis in cells. The structure-activity-relationship (SAR) study identified structural moieties critical for the inhibitory effects toward MDM2-MDM4 E3 ligase, the targeted degradation of MDM4 and FTH1 in cells, and anti-proliferation activity. Lead optimization led to the development of compound **MMRi71** with improved activity. In addition to accumulating p53 proteins in wt-p53 bearing cancer cells as expected of any MDM2 inhibitors, **MMRi71** effectively kills p53-null leukemia cells, an activity that conventional MDM2-p53 disrupting inhibitors lack. This study provides a prototype structure for developing MDM4/FTH1 dual-targeting inhibitors as potential cancer therapeutics.

## 1. Introduction

The tumor suppressor p53 (TP53) is a well-established drug target whose activation has been shown to induce tumor regression in several models [1,2,3]. In normal and cancer cells, p53 activity is inhibited mainly by MDM2 (murine double minute 2) and MDM4, RING domain-containing proteins [4,5]. MDM4 (also known as MDMX) is the only MDM2 homolog that is expressed at levels higher than MDM2 due to its increased expression and protein stability in most cancer types. The N-terminus of both MDM2 and MDM4 binds to the p53 transactivation domain and inhibits the p53-dependent transcriptional transactivation of the downstream genes, while the C-terminal RING domain of MDM2 can bind to E2 ubiquitin-conjugating enzymes for p53 ubiquitination and to the RING domain of MDM4 to form heterodimers [5,6]. The development of small molecule inhibitors of the MDM2-p53 interaction has been a research focus for decades since the discovery of the first-in-class small molecule Nutlin3a [7], and several Nultlin3-like MDM2 inhibitors are in clinical trials [8]. These inhibitors are intended to bind to the p53-binding pocket of MDM2, which prevents MDM2 from binding to p53, thus releasing p53 from MDM2-mediated inhibition and eliciting tumor suppression in a p53-dependent manner. Accordingly, p53 mutation confers intrinsic and acquired resistance to MDM2-p53 disruptor inhibitors [9]. Further, this type of MDM2 inhibitor is challenged with resistance conferred by MDM4 overexpression in cancer cells since MDM4 can bind to and inhibit the p53 transactivation domain in the absence of MDM2 [10,11,12].

The significance of the RING-RING interaction of MDM2-MDM4 in regulating the p53 degradation and p53 function in vivo has been established by in vitro biochemical studies [13,14,15] and mouse genetic modeling [4,16,17,18]. Crystallographic studies on the RING heterodimers of MDM2-MDM4 found that the RING heterodimer forms a new E2—interacting interface in the MDM2 RING domain [19]. We have shown how MDM2 alone only mediates the multi-monoubiquitination of p53, and MDM2-MDM4 heterodimers mediate p53 polyubiquitination for degradation. Both the mono and polyubiquitination activity of MDM2 depend on the same E2—binding interface, and all three components of the MDM2/MDM4/p53 tertiary complex are substrates for polyubiquitination by the RING heterodimer E3 ligase [15]. Further, in a mouse model expressing an intact RING domain but an E3-dead MDM2L466A mutant, we have shown that the E3 ligase activity of MDM2-MDM4 RING heterodimers is not only essential for p53 regulation in vivo but is also required for cell cycle progression independent of p53 [20]. We explored the RING-RING interaction of MDM2-MDM4 as a new targeting interface for drug development and identified several primary hits with a potent inhibitory effect on the MDM2-MDM4-mediated ubiquitination of p53 while lacking inhibitory activity toward the NEDD4-1 E3 ligase and the control enzyme. The hits were designated as MMRi for the MDM2-MDM4 RING inhibitors [21]. In a secondary screen, we identified **MMRi62** to be a modifier of the MDM2-MDM4 E3 ligase activity, inducing MDM4 degradation in cells and p53-independent apoptosis in leukemic cells [22], as well as ferroptosis in pancreatic cancers associated with the degradation of FTH1 (Ferritin Heavy Chain 1) [23]. Here, we reported structural activity relationship studies (SAR) leading to the identification of **MMRi71** as an inhibitor of the E3 ligase activity of MDM2-MDM4, an inducer of MDM4 and FTH1 dual protein degradation and p53-independent apoptosis in leukemia cells. Interestingly, the anti-cancer potency of **MMRi67**, the first lead in the development of the structurally optimized prodrug **MMRi71**, was not readily apparent until benzoic acid was introduced as an ethyl benzoate ester. Conversely, the inhibition of E3 ligase activity is only observed when **MMRi67** is in its benzoic acid form.

## 2. Results and Discussion

### 2.1. Structural Requirement for the Anti-Proliferative Activity of ***MMRi62*** and ***MMRi67*** Derivatives

**Figure 1:**
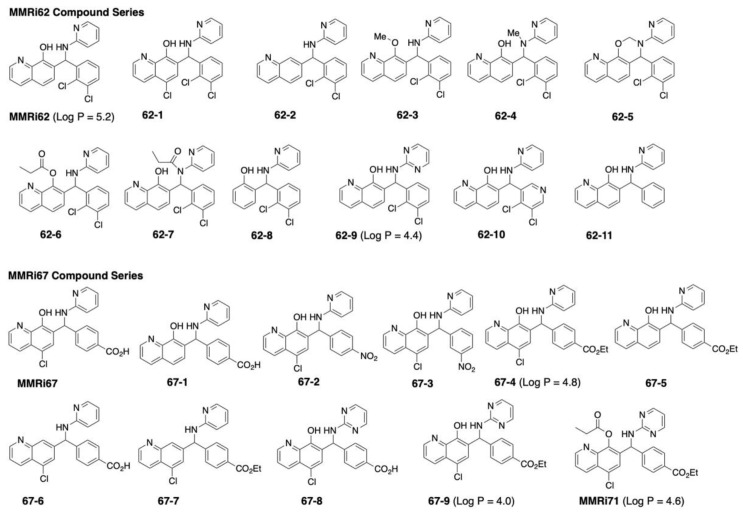
Structures of **MMRi62** and **MMRi67** compound series.

Primary hits of **MMRi6** are quinolinols identified in an enzyme-based HTS for inhibition toward the MDM2-MDM4 RING-domain mediated E3 ligase reaction [21]. With available **MMRi6** analogs from the Hit2Lead library, we performed secondary screens and identified two analogs with unique properties: **MMRi62** binds to preformed MDM2-MDM4 RING heterodimers for preferential MDM4 ubiquitination and acts as an MDM4-degrader in cells with potent apoptosis-inducing activity; while **MMRi67** was the most potent MDM2-MDM4 E3 ligase inhibitor, it lacked MDM4 degradation and pro-apoptotic activity in cells [22]. To our knowledge, **MMRi67** is the only small molecule with demonstrated inhibitory activity against the MDM2-MDM4 E3 ligase. The capability of p53-independent apoptosis induction by **MMRi62** promises the potential application of the quinolinol derivatives as novel cancer therapies for killing p53-mutant drug-resistant cancer cells. This potential motivated us to perform SAR studies on **MMRi62** and **MMRi67** for lead optimization (Figure 1) with the hope of identifying a compound that possessed both MDM2-MDM4 E3 ligase inhibition and cancer cell growth inhibition activities.

Anti-proliferation assays were performed for the compounds shown in Figure 1 using the wtp53-bearing NALM6 (p53-dependent) and shP53NALM6 (p53-independent) leukemia cell lines. The chemical structures of **MMRi62** and **MMRi67** differed in a few positions. **MMRi62** had two chlorines on the phenyl ring and none on the quinolinol ring, while **MMRi67** had chlorine on the quinolinol ring and a carboxylic acid on the phenyl ring (Figure 1). To test the impact of a quinolinol chloro-substituent on the **MMRi62** series, we synthesized **62-1**, which was added to this substituent. This analog showed a somewhat weaker cell growth inhibitory effect in the NALM6 and shP53NALM6 cells than **MMRi62** (Table 1). Analog **62-1** lost a comparatively greater extent of activity in the p53-independent cell line. To test the importance of the quinolinol chlorine on the **MMRi67** series, we synthesized **67-1**, which lacked this substituent. This compound was found to have a similar (weak) leukemic cell growth inhibition to **MMRi67**. We concluded from these data that the quinolinol chlorine substituent modestly reduced compound cytotoxicity.

We next probed the modification of the carboxylic acid on the phenyl ring of **MMRi67**. Analog **67-2**, which replaced the carboxylic acid in **MMRi67** with a similar electron-withdrawing nitro group, and its corresponding 3-nitro analog, **67-3,** both showed over 20 times better IC_50_ against NALM6 and shP53NALM6 cell growth than **MMRi67**. We hypothesized that if the carboxylic acid in **MMRi67** existed substantially in its charged carboxylate form, its cell permeability would be low, thereby reducing its ability to inhibit cancer cell growth. Indeed, the ethyl ester analog **67-4** proved to be a much more potent cell growth inhibitor, with over a 50-fold lower IC_50_ than the **MMRi67** of 0.22 μM and 0.21 μM in the NALM6 and shP53NALM6 cells, respectively. Analog **67-5**, an ethyl ester derivative that lacks the chloro functionality on the quinolinol, showed a modestly better antiproliferative activity than **67-4** (Table 1).

The critical role of the quinolinol’s hydroxyl moiety in both **MMRi62** and **MMRi67** was determined. Analogs lacking the hydroxyl, **62-2**, **67-6,** and **67-7**, all showed poor activity in the cell proliferation assays compared to their lead compounds. Analog **67-6** completely lost its antiproliferative activity in the leukemia cells with IC_50_ > 100 μM. Analog **67-7**, with the ethyl ester group substitution but lacking the hydroxyl, showed an 80-150-fold weaker cell proliferation inhibitory activity compared to **67-5**.

The importance of the respective protons on the phenol and amine was additionally tested in the **62** series. Methylated phenol **62-3** was 70-fold and 172-fold less active than **MMRi62** in the NALM6 and shP53NALM6 cells, respectively. Methylated amine **62-4**, cyclic aminal **62-5,** and an esterase-labile ester analog **62-6** also showed a weaker anti-leukemic cell growth activity compared to the parent **MMRi62**. Interestingly, the amide analog **62-7,** lacking the amine proton, showed a modestly better antiproliferative activity. The amide bond was not expected to be very metabolically labile; instead, the intact **62-7** was expected to be responsible for the antiproliferative activity. These data may support some covalent reactivity with biochemical targets (the *N*-acetyl-2-aminopyridine could be better solvolysis leaving group than 2-aminopyridine,). An **MMRi62** analog containing the phenol but lacking quinoline heterocycle **62-8** also showed increased IC_50_s against cell growth.

The water solubility of these MMRi compounds, estimated by a calculated logP, indicated that they were slightly lipophilic (**MMRi62** LogP = 5.1, analog **67-4**, LogP = 4.8). We hypothesized that their water solubility, which would aid in formulation, could be enhanced by the addition of heteroatoms to the 2-aminopyridine ring. Therefore, a pyrimidine domain was substituted in **MMRi62** to generate **62-9** and in **MMRi67** to generate analogs **67-8** and **67-9**. The predicted LogP of the pyrimidine analog **62-9** was 4.4 and for the pyrimidino analog **67-9** was 4.0. The analysis of these more water-soluble analogs in the antiproliferation of leukemic cells indicated that their activities were only modestly diminished, suggesting that the pyrimidine modification was tolerated. Analog **62-10**, with additional nitrogen in the 2,3-dichlobenzene ring, was 100 times less active than **MMRi62**, indicating that not all changes were tolerated on this aryl ring. In addition, it should be noted that **62-11** lacked substitution on the benzene ring and was ten times less potent than **MMRi62**. Analogs **62-10** and **62-11** were likely less active than **MMRi62** for different reasons since their impact on the electron density of their respective arene is opposite: **62-10**’s pyridine should be more electron-poor than **MMRi62**’s dichlorobenzene, while **62-11**’s unsubstituted phenyl ring should be more electron-rich than **MMRi62**’s dichlorobenzene. Such electron-density changes can impact the solvolysis potential of their respective benzylic amines.

**Scheme 1:**
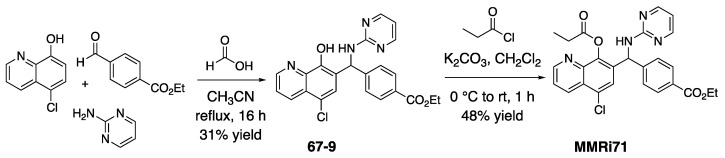
Synthesis of **MMRi71**. A representative synthesis is shown for **MMRi71**, the lead compound our study concludes with. For synthesis details of **MMRi71**, **MMRi62,** and **MMRi67** series see the M and M and Supporting Information.

Although the phenol is critical to the antiproliferative activity of the MMRi compounds, it can be seen as a potential source of molecular aggregation, either via a hydrogen bonding network or transient metal chelation [24]. Thus, to mitigate these potential unwanted reactivities [25], an esterase-labile propionate moiety was introduced to the lead pyrimidinyl analog **67-9** to generate the prodrug **MMRi71** (Scheme 1). We noted that **MMRi71** could also be converted to **67-8** if both of its esters are hydrolyzed by cellular esterases. **MMRi71**, an IC_50_ of 0.23 μM in the NALM6 cells and 0.29 μM in the shP53NALM6 cells, showed improved p53-independent antiproliferative activity in leukemia cells and was selected as a potential candidate for further investigation.

### 2.2. ***MMRi67*** Is a Bona Fide Inhibitor of MDM2-MDM4 E3 Ligase

**Figure 2:**
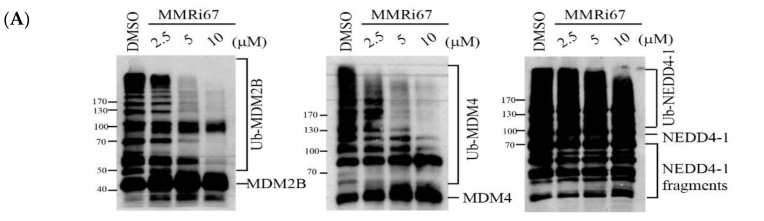

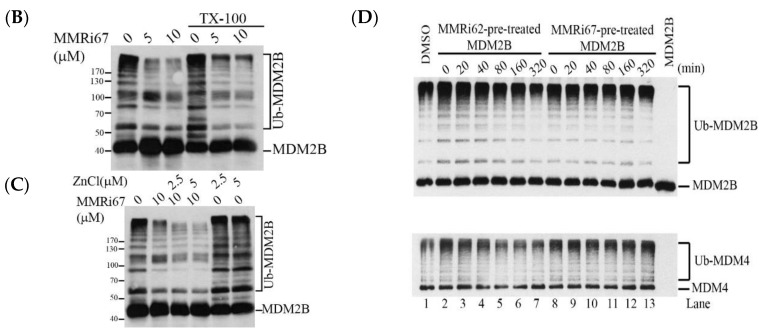
**MMRi67** is a small molecule inhibitor of MDM2-MDM4 E3 ligase activity. (**A**) **MMRi67** inhibits the ubiquitination of MDM2B (left) and MDM4 (middle) by MDM2B-MDM4 E3 complex but not NEDD4-1 (right) in the in vitro E3 ligase assays. WB analysis of MDM4, MDM2B, and NEDD4-1 using specific antibodies after in vitro ubiquitination reaction in the presence of solvents or indicated concentrations of **MMRi67**. The smearing bands of ubiquitinated MDM4 (Ub-MDM4), ubiquitinated MDM2B (Ub-MDM2B), and ubiquitinated NEDD4-1 (Ub-NEDD4-1) are indicated. (**B**) The E3 ligase inhibitory activity of **MMRi67** is not abolished by the presence of 0.01% Triton X-100. Same procedure as in (**A**) except the ubiquitination reaction was performed in the presence of 0.01% Triton X-100. Only MDM2B data are shown. (**C**) The E3 ligase inhibitory activity of **MMRi67** is not abolished by the presence of increasing concentrations of Zn to neutralize the potential chelating capability of **MMRi67**. The same procedure as in (**A**) except for the presence of indicated concentrations of Zn. Only MDM2B data are shown. (**D**) Preincubation of MDM2B proteins with **MMRi67** does not abolish MDM2B E3 ligase activity. Same procedure as in (**A**) except MDM2B was preincubated with either 10 μM **MMRi62** or **MMRi67** for the indicated min before 10-times dilution and was used for ubiquitination reaction; the preincubation did not affect the ubiquitination of MDM2B or MDM4 by the MDM2B-MDM4 E3 complex.

False positive hits in biochemical or cell-based screens with so-called PAINS (pan assay interference compounds) are of concern and hinder lead optimization [26,27]. We reasoned that **MMRi62** and **MMRi67** are unlikely PAINS since we used NEDD4-1 as a non-specific control E3 ligase in validation assays [21], which was further validated in this study by the selectivity of **MMRi67** for Mdm2B versus NEDD4-1 (Figure 2A). Moreover, **MMRi62** and **MMRi67** have a distinct effect on MDM2-MDM4 E3 ligase activity, MDM4 degradation, and apoptosis induction despite their subtle structural difference [22]. The identification of PAINS from a real hit requires more than simply running a filter screen [28,29]. However, some chemical behaviors of PAINS can be tested, including protein reactivity, colloidal aggregation, and metal chelation [26,27]. To rule out colloidal aggregator activity, we performed an E3 ligase assay in the presence or absence of 0.01% of the non-ionic detergent, Trixton-X-100. Our results indicated that the **MMRi67**-mediated inhibition of MDM2-MDM4 E3 ligase was not affected in the presence of the detergent (Figure 2B).

The structure of **MMRi67** predicts the metal chelation potential [24]. Since Zn is required for maintaining the RING domain structure, it is possible that **MMRi67** chelates away Zn and collapses the RING structure, leading to the dissociation of the RING-RING interaction between MDM2-MDM4 and the loss of the E3 ligase activity. We hypothesized that an excessive amount of Zn would neutralize the chelating activity of **MMRi67** and cancel its inhibition of MDM2-MDM4 E3 ligase activity. We performed an E3 ligase assay in the presence of Zn, and our results indicated that the presence of Zn did not affect the ability of **MMRi67** to inhibit MDM2-MDM4 E3 ligase activity.

The preincubation of MDM4 proteins with either **MMRi62** or **MMRi67** for a period from 20 to 320 min at 10 μM did not affect the E3 ligase activity of the MDM2-MDM4 E3 complex (data not shown). However, the preincubation of MDM2B proteins with **MMRi62** but not **MMRi67** inhibited MDM2-MDM4 E3 ligase activity (Figure 2D), suggesting that covalent binding contributes to some extent to **MMRi62**-mediated effects on the MDM2B-MDM4 E3 ligase reaction (e.g., via the alkylation of a quinone methide formed from 2-aminopyridine loss via the solvolysis of **MMRi62**. See Supporting Information for further discussion). This covalent binding potential of **MMRi62** is similar to that of a macrophage migration inhibitory factor (MIF) inhibitor [30], which shares structural similarities with **MMRi62**. However, the covalent binding is not involved in the inhibitory mechanism of **MMRi67** toward the MDM2-MDM4 E3 ligase in vitro. Taken together, our results suggest that **MMRi67** bears a specific inhibitory activity toward the MDM2-MDM4 E3 ligase.

### 2.3. Structural Requirement of ***MMRi67*** Derivatives for Inhibitory Activity toward MDM2-MDM4 E3 Ligase

**Figure 3:**
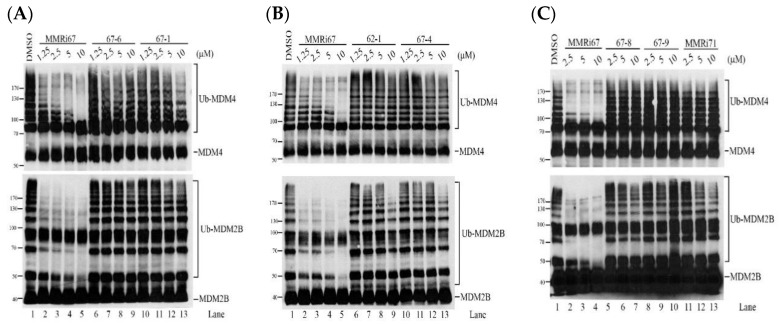
Assessment of the inhibitory effect of **MMRi67** derivatives toward MDM2B-MDM4 E3 ligase in the in vitro E3 ligase assays. (**A**) Comparison of **MMRi67** with hydroxyl to hydrogen substituted (**67-6**) and chloro to hydrogen substituted (**67-1**) analogs in inhibition of the MDM4 and MDM2B ubiquitination in vitro. WB analysis of MDM4 or MDM2B using specific antibodies after in vitro ubiquitination reaction in the presence of a solvent or indicated compound at the indicated concentrations. The smearing bands of ubiquitinated MDM4 (Ub-MDM4) and MDM2B (Ub-MDM2B) are indicated. (**B**) Same analysis as in (**A**) except for the comparison of **MMRi67** with carboxylic acid substituted analogs **62-1** and **67-4** in the inhibition of MDM4 and MDM2B ubiquitination in vitro. (**C**) Same analysis as in (**A**) except for the comparison of **MMRi67** with pyridine substituted analogs **67-8** and pyridine, hydroxyl, and carboxylic acid substituted analogs **67-9** and **MMRi71** in the inhibition of MDM4 and MDM2B ubiquitination in vitro.

To identify the pharmacophore of **MMRi67**, it involved the inhibition of the MDM2-MDM4 E3 ligase, and some of its analogs (Figure 1) were evaluated in MDM2-MDM4 E3 ligase assays. Analogs **67-1**, lacking the quinolinol chlorine substituent, and **67-6**, lacking the phenol, both lost the strong E3 ligase inhibitory activity of **MMRi67**. However, **67-1** showed a modest inhibition at 10 μM (Figure 3A). It is possible that the phenol of **MMRi67** is involved in a critical molecular interaction that can be impacted by the phenolic pKa (which would be lowered by the quinolinol chlorine substituent). To check whether the benzoic acid in **MMRi67** plays a critical role in its E3 ligase activity for MDM2 and MDM4, **62-1** with a dichlorophenyl substitution and **67-4** (Figure 1) containing the ethyl benzoate were evaluated with E3 ligase assays. The results showed weak inhibition of MDM2 and MDM4 ubiquitination at concentrations of 10 μM for both the **62-1** and **67-4** derivatives, indicating that the E3 ligase inhibitory action of **MMRi67** strongly depends on the carboxylic acid substituent (Figure 3B). It is possible that the carboxylic acid is also involved in a critical molecular interaction with its biochemical target.

A pyridine-to-pyrimidine change in **MMRi67** to generate analog **67-8** resulted in the loss of the MDM4 E3 ligase inhibitory effect; however, partial inhibition of the MDM2B E3 ligase at high concentrations was observed. Analog **67-9**, containing a pyrimidine and ethyl benzoate, lost both MDM4 and MDM2B E3 ligase effects. **MMRi71**, containing a pyrimidine and ethyl benzoate as well as a phenolic propionate ester, lost activity to inhibit MDM4 ubiquitination but retained an inhibitory effect on MDM2B ubiquitination in a dose-dependent manner (Figure 3C). Overall, the hydroxyl, chloro, carboxylic acid, and pyridine functionalities of **MMRi67** all impacted its MDM4-MDM2B E3 ligase inhibition.

### 2.4. Induced MDM4 and FTH1 Degradation Is Associated with the Pro-Apoptotic Activity of ***MMRi67*** Derivatives

**Figure 4:**
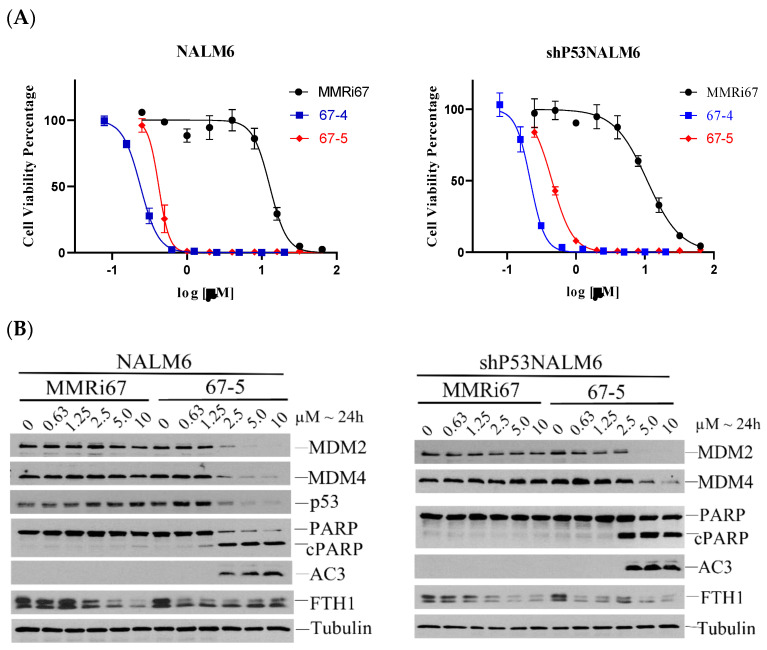
Anti-proliferation activity of **67-5** is associated with the downregulation of MDM2, MDM4, and FTH1 protein expression and caspase 3 activations. (**A**) Improved anti-proliferation activity of **67-4** and **67-5** compared with **MMRi67**. Antiproliferation assays in NALM6 and shp53NALM6 cells. The IC_50_ in NAML6 cells was 12.8 μM for **MMRi67** and 0.38 μM for 67-5. The IC_50_ in shP53NAML6 cells was 10.9 μM for **MMRi67** and 0.45 μM for **67-5**. (**B**) Improved antiproliferation activity of **67-5** is associated with the downregulation of MDM2, MDM4, and FTH1, as well as caspase activation and PARP cleavage. WB analysis of indicated proteins and apoptotic effect induced by **67-5** compared to **MMRi67** in both NALM6 and shP53NALM6 cells.

The p53-independent pro-apoptotic activity of the quinolinol compound **MMRi62** was associated with an induced MDM4 and FTH1 degradation in cells [22,23]. To determine whether the increased antiproliferative activity of the potent **MMRi67** analogs was also associated with p53-independent apoptosis and MDM2/MDM4 and FTH1 protein degradation, we conducted a Western blot analysis for the activated Caspase 3 and caspase-mediated PARP cleavage. Consistent with its improved anti-proliferation activity, analog **67-5**, lacking the quinolinol chloro of **MMRi67**, and bearing the ethyl benzoate, induced the downregulation of MDM4/MDM2, caspase 3 activation, and PARP cleavage in a concentration-dependent manner (Figure 4A,B). Moreover, the pro-apoptotic effect of **67-5** was independent of p53 since the apoptotic response to **67-5** between the NALM6 and shp53NALM6 cells showed no difference. Similar results were obtained with **67-4** (data not shown). These results demonstrate that the improved cell-killing ability of **67** derivatives uses a similar mechanism of action as **MMRi62**, i.e., MDM4 degradation is a necessary molecular event associated with apoptosis induction [22]. Although the **67-5** effectively induced downregulation of FTH1, as far as the apoptosis of leukemia cells is concerned, it appeared that MDM2/MDM4 degradation, but not FTH1 degradation, was associated with apoptosis induction, since **MMRi67** also induced FTH1 degradation at concentrations ≥2.5 μM yet did not elicit any apoptotic response (Figure 4B). Nevertheless, we could not exclude the role of FTH1 degradation in predisposing cells to apoptosis since FTH1 is a ferroptosis target whose depletion increases reactive oxygen species (ROS) and induces ferroptosis in other cell types [23,31].

**Figure 5:**
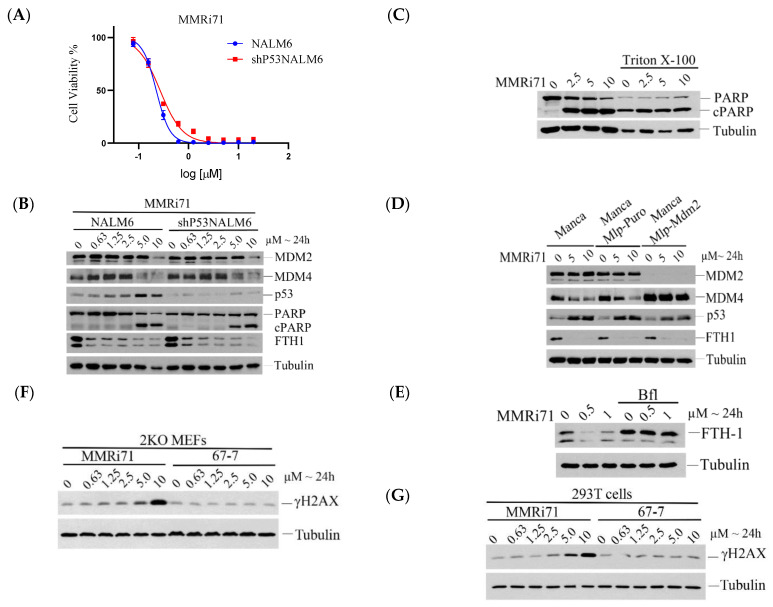
Characterization of **MMRi71** in cells. (**A**) The anti-proliferation activity of **MMRi71** is p53-independent with IC_50_ of 0.23 μM in NALM6 and 0.29 μM in shp53NALM6 cells. (**B**) WB analysis shows how **MMRi71** induces MDM4/MDM2 and FTH1 protein degradation and apoptotic effect in NALM6 and shP53NALM6 cells. (**C**) The pro-apoptotic effect of **MMRi71** is not affected by the presence of 0.0025% Triton X-100 in NALM-6 cells, as shown by the WB analysis of apoptotic PARP cleavage. (**D**) **MMRi71**-induced MDM4 degradation is MDM2 dependent. WB analysis showing Mdm2 knockdown in Manca-mlp-Mdm2 cells and abolished **MMRi71**-induced MDM4 degradation. (**E**) **MMRi71**-induced FTH1 degradation is lysosome dependent. WB showed the rescue of **MMRi71**-induced FTH1 degradation by 50 nM lysosome inhibitor Bafilomycin A1 (Bfl) in NALM6 cells. (**F**) (**G**) DNA damage induction by **MMRi71** and **67-7**. Mouse p53/Mdm2 double knockout MEFs (2KO) (**F**) or human 293T cells (**G**) were treated with indicated concentrations of **MMRi71** and **67-7** for 24 h at the indicated concentrations, followed by WB analysis for gammaH2AX and Tubulin with specific antibodies. Tubulin served as a protein loading control.

Analog **MMRi71** showed strong anti-leukemic activity in the cell proliferation inhibitory assay along with the dose-dependent inhibition of MDM2B E3 ligase activity. We hypothesized that the structural modifications in **MMRi71** (compared to **MMRi67**) would increase water solubility (pyridine changed to pyrimidine) and decrease molecular self-aggregation (via hydrogen bonding or through metals involving the phenol, changed to the propionate ester) while maintaining the cell permeability potential of the candidate (by applying the ethyl benzoate in place of the benzoic acid) and its optimal effect on cellular targets (given that esters can be hydrolyzed in cells). To test this, anti-proliferation assays showed that **MMRi71** had the same potency in inhibiting the growth of NALM6 and shP53NALM6 cells with an IC_50_s of 0.2 μM (Figure 5A), indicating p53-independent cytotoxicity. Interestingly, the phenol ester **MMRi71** was substantially more cytotoxic than phenol ester **62-6** even though their parent phenols **67-9** and **MMRi62** have similar cell growth IC_50′_s. The greater aqueous solubility of the pyrimidine lends to **MMRi71** may contribute to a more rapid ester hydrolysis and to its active form, phenol **67-9**.

**MMRi71**-treated NALM6 and shP53NALM6 cells were used in the Western blot analysis for changes in the MDM4 and FTH1 proteins. The **MMRi71** treatment induced effective downregulation of MDM4 and FTH1 in both NALM6 and shP53NALM6 cells, and the induced apoptosis shown by the PARP cleavage indicated a p53-independent mechanism (Figure 5B). To test whether **MMRi71**-induced apoptosis was not a result of colloidal aggregation, we performed cellular experiments in the presence of Triton X-10. Since the presence of 0.01%, Trixton-X-100 appeared to induce apoptotic cell death in the NALM6 cells, and the tolerable concentrations of Triton X-100 were between 0.001 and 0.0025% (data not shown). We performed cellular experiments in the presence of 0.0025% Triton X-100, and the results showed that **MMRi71** induced a similar extent of apoptotic responses indicated by the cleaved PARP in the presence of the detergent (Figure 5C), suggesting that **MMRi71**-induced apoptosis was not a result of colloidal aggregation.

We have shown that **MMRi62**-induced MDM4 degradation was MDM2-dependent [22] and that **MMRi62**-induced FTH1 degradation was dependent on a lysosomal pathway [23]. To determine if the **MMRi71**-induced MDM4 degradation occurred via the same mechanism as **MMRi62**, we performed a WB analysis of MDM4 degradation by **MMRi71** using a pair of MDM2-high MANCA lymphoma cells in which MDM2 was either stably knocked down by lentivirus-mediated microRNA (miRNA) or left untouched with a control miRNA. Our results indicated that MDM4 expression levels were elevated in MDM2-knockdown MANCA-mlp-MDM2 cells and that treatment with **MMRi71** did not induce MDM4 degradation. The elevated MDM4 expression in the MDM2-knockdown cells was consistent with the report that MDM2 promoted the ubiquitination and degradation of MDM4 [32]. The abolishment of MDM4 degradation by **MMRi71** in the absence of MDM2 in MANCA-mlp-MDM2 cells suggests that **MMRi71**-induced MDM4 degradation is MDM2-dependent (Figure 5D). To determine whether the **MMRi71**-induced FTH1 degradation was lysosome dependent, we performed a rescue experiment with the lysosome inhibitor Bafilomycin A1 (BAF1). Our results showed that BAF1 fully rescued FTH1 in the NALM6 cells (Figure 5E). These results suggested that **MMRi71** induced MDM2-dependent proteasomal degradation of MDM4 and the lysosome-dependent degradation of FTH1, the same mechanisms of action used by **MMRi62** [22], in addition to its potential inhibitory activity toward MDM2-MDM4 E3 ligase activity.

The substantially improved activity of **MMRi71** over **MMRi67** may be due to its increased covalent binding capability or its improved cellular permeability, or both. **MMRi62**, which has structural similarities to **MMRi71**, did show some covalent binding effects to MDM2 (Figure 2D). Based on the studies with a quinolinol analog MIF inhibitor, covalent binders in this molecular class can be specific inhibitors [30]. Whether **MMRi71** is a covalent inhibitor targeting cellular MDM2-MDM4 for degradation needs further characterization in future studies.

Quinolinol compounds were reported to cause DNA damage via chelating metal ions and the generation of ROS [33]. To test whether **MMRi71** caused DNA damage, we used **MMRi71** and the inactive compound **67-7,** which lacked the metal chelating capability to treat the p53/Mdm2-double knockout (2KO) MEFs and human 293T cell lines, followed by detecting γ-H2AX which is an indicator of DNA damage [34]. The use of 2KO MEFs and 293T cells will exclude the apoptosis-associated γ-H2AX signal due to apoptotic DNA fragmentation since these two cell types are resistant to apoptosis. Our results showed that **MMRi71** but not the inactive **67-7** increased γ-H2AX slightly at 5 μM and significantly at 10 μM in both the cell lines (Figure 5F,G). These results suggest that **MMRi71** at concentrations of ≥5 μM has the potential to induce DNA damage, possibly by **MMRi71**-induced ROS. These results are consistent with our observation that **MMRi62** induces ROS generation in pancreatic cells [23]. ROS induced by **MMRi62**-like compounds likely involves both metal chelation and FTH1 degradation, which releases ferrous iron, although metal chelation is not involved in its action on the E3 ligase activity (Figure 2C). Since **MMRi71** induced apoptosis at ≥5 μM at 24 h in the NALM6 cells (Figure 5C) and **67-7** did not generate ROS is an inactive compound, these results conclude that ROS generation is required for the antitumor activity of **MMRi71**. However, we speculate that ROS-induced DNA damage itself is not the major mechanism contributing to cell killing by **MMRi62**-like compounds since they have a high cancer-selective toxicity. We have shown that **MMRi62** inhibits leukemic NALM6 (B-cell precursor leukemia) cell growth at a 125-fold potency compared to its inhibition of normal peripheral blood mononuclear cells (PBMCs) [22]. This 125-fold difference in **MMRi62** sensitivity is not likely the result of DNA damage since DNA damage by radiation kills normal human lymphocytes, and B cell lymphoma cells have a comparable capability with the D_0_ of 1.95Gy for lymphocytes and of 1.38Gy for Burkitt’s lymphoma cell [35], where D_0_ is the radiation dose required to reduce the fraction of the surviving cells to 37% of its previous value. Accordingly, similar to **MMRi62**, the mechanisms of action for **MMRi71** should involve multiple drug targets that predispose cancer cells to its selective toxicity, which warrants identification in future studies.

## 3. Materials and Methods

### 3.1. Representative Chemistry Methods

#### 3.1.1. Ethyl 4-((5-Chloro-8-hydroxyquinolin-7-yl)(pyrimidin ylamino)methyl)benzoate (**67-9**) Synthesis

To a 250 mL dry round-bottomed flask equipped with a reflux condenser, 2-aminopyrimidine (4.3 g, 45.3 mmol, 1.2 equiv), ethyl 4-formylbenzoate (6.8 g, 38.2 mmol, 1.0 equiv), and 5-chloro-8-hydroxyquinoline (8.2 g, 45.8 mmol, 1.2 equiv) were dissolved in CH_3_CN (100 mL). Following the addition of formic acid (1.4 mL, 37.1 mmol 1.0 equiv), the solution was stirred at reflux for 16 h. The solution was allowed to cool to rt, was concentrated, and resuspended in acetone. The heterogenous mixture was filtered, and the precipitate was washed with cold acetone and hexanes to give **67-9** as a white solid (5.2 g, 31% yield). mp = 150–151 °C; ^1^H NMR (300 MHz, CDCl_3_) δ 8.80 (d, *J* = 4.2 Hz, 1H), 8.49 (d, *J* = 8.6 Hz, 1H), 8.30 (d, *J* = 4.8 Hz, 2H), 7.99 (d, *J* = 7.9 Hz, 2H), 7.55 (dd, *J* = 19.6, 10.2 Hz, 4H), 6.79 (d, *J* = 8.2 Hz, 1H), 6.58 (t, *J* = 4.9 Hz, 1H), 6.39 (d, *J* = 8.3 Hz, 1H), 4.34 (q, *J* = 7.2 Hz, 2H), 1.36 (t, *J* = 7.2 Hz, 3H); ^13^C NMR (75 MHz, CDCl_3_) δ 166.3, 161.5, 158.2, 148.7, 148.5, 146.6, 138.7, 133.3, 129.8, 129.4, 127.0, 126.8, 125.6, 123.6, 122.5, 120.7, 111.5, 60.9, 54.8, 14.3; IR neat film: 3293, 2978, 1716, 1583, 1496 cm^−1^; HRMS (ESI) calculated for [C_23_H_20_ClN_4_O_3_] 390.1004, found 390.1011.

#### 3.1.2. Ethyl 4-((8-(Propionyloxy)quinolin-7-yl)(pyrimidin-2-ylamino)methyl)benzoate (**MMRi71**) Synthesis

In a 5 mL dry round-bottomed flask, analog **67-9** (5.0 g, 11.5 mmol, 1.0 equiv) was dissolved in dry CH_2_Cl_2_ (100 mL) under an argon atmosphere. Potassium carbonate (3.18 g, 23.0 mmol, 2.0 equiv) was added, and the solution was cooled to 0 °C. Propionoyl chloride (1.0 mL, 11.5 mmol, 1.0 equiv) was then added to the solution. The mixture was allowed to warm to rt and stirred for 1 h. The reaction mixture was then filtered through Celite and washed with CH_2_Cl_2_. The supernatant was then treated with 1 g of DMT-functionalized silica gel and stirred for 15 min. The mixture was filtered and concentrated. The resulting crude solid was resuspended in Et_2_O and washed with deionized water. The organic layer was dried over Na_2_SO_4_ and then concentrated. The crude mixture was purified by flash column chromatography (silica gel, 50% ether:hexanes) to yield **MMRi71** as a greenish-white solid (2.53 g, 48% yield). mp = 178–179 °C; ^1^H NMR (400 MHz, CDCl_3_) δ 8.91 (d, *J* = 2.8 Hz, 1H), 8.50 (d, *J* = 6.8 Hz, 1H), 8.23 (d, *J* = 4.8 Hz, 2H), 8.00 (d, *J* = 8.4 Hz, 2H), 7.54 (s, 1H), 7.50 (dd, *J* = 8.5, 4.2 Hz, 1H), 7.43 (d, *J* = 8.1 Hz, 2H), 6.83 (d, *J* = 7.8 Hz, 1H), 6.57 (t, *J* = 4.8 Hz, 1H), 6.10 (d, *J* = 7.8 Hz, 1H), 4.36 (q, *J* = 7.1 Hz, 2H), 2.68 (q, *J* = 7.6 Hz, 2H), 1.37 (t, *J* = 7.1 Hz, 3H), 1.20 (t, *J* = 7.5 Hz, 3H); ^13^C NMR (101 MHz, CDCl_3_) δ 172.2, 166.2, 161.3, 158.1, 151.2, 145.5, 144.6, 142.0, 134.3, 133.0, 130.0, 129.8, 129.0, 127.1, 126.7, 125.7, 122.4, 111.8, 61.0, 53.8, 27.3, 14.3, 9.0. IR neat film: 3270, 2982, 1773, 1716, 1578, 1491 cm^−1^; HRMS (ESI) calculated for [C_26_H_23_ClN_4_NaO_4_] 513.1300, found 513.1307.

### 3.2. Biological Assays and Methods

#### 3.2.1. Cell Culture and Small-Molecule Compounds

NALM6 and shP53NALM6 leukemic cell lines were cultured in an RPMI-1640 medium supplemented with 10% fetal bovine serum, 50 U/mL penicillin, and 50 μg/mL streptomycin. The shP53NALM6 cell line was established using pLKO.1-p53 (purchased from Sigma) (Plasmid #19119) [36] followed by puromycin selection at 1 μg/mL for 2 days, then a clonal expansion in the puromycin-free medium. MANCA, MANCA-mlp-puro, and MANCA-mlp-MDM2 were generous gifts from Prof. Jill Bargonetti (Hunter College, CUNY, NY, USA) and were generated as described previously [37] and maintained in a 10% FBS-Pen/Strep- RPMI-1640 medium. Small molecule compounds were synthesized, purified, and characterized in-house and were dissolved in DMSO as 10 mM stocks for cell proliferation assays.

#### 3.2.2. Western Blotting, In Vitro Ubiquitination, and Apoptosis Analysis

The Western blotting procedure and antibodies for the target proteins were described previously [22]. In vitro assays for ubiquitination by MDM2B-MDM4 were performed as described previously with minor modifications [15]. Briefly, the reactions were carried out at 30 °C for 1 h in a volume of 20 μL reaction in the presence of different concentrations of compounds or vehicle solvent DMSO, followed by a WB of p53 with DO-1, MDM2 with rabbit anti-MDM2 (MDM2 (D1V2Z) (#86934, Cell Signaling Technology, Danvers, MA 01923, USA), or MDM4 with a rabbit anti-MDM4 antibody (Proteintech, Rosemont, IL 60018, USA, Cat no: 17914-1-AP). The apoptotic response to compounds was measured by Western blotting using specific antibodies from Cell Signaling Technology, Danvers, MA 01923, USA) for activated caspase 3 (Cleaved Caspase-3 (Asp175) (5A1E) (#9664) and PARP (PARP Antibody #9542).

#### 3.2.3. IC_50_ Measurement and Analysis

The procedure was described previously [22]. Briefly, cells at 10,000/well were plated in 96-well plates at 100 µL/well, and compounds of different concentrations were added to each well at 100 µL/well. After 70 h treatment, 40 µL of 6x resazurin stock solution was added to each well, followed by a 2 h development of fluorescent metabolites by viable cells before reading OD600 in the BioTek Synergy 2 Microplate Reader. The IC_50_ values were obtained by the Chou-Median-Effect Equation using CompuSyn software [38], and dose-effect curves were obtained by GraphPad using the affected fractions of compound-treated wells normalized against no-drug control wells with a non-liner regression model.

## 4. Conclusions

In summary, the antiproliferative activity of these potent MMRi67 derivatives was associated with their effects on the dual degradation of MDM4 and FTH1 compared to MMRi67. The SAR results from MMRi67 derivatives identified the hydroxyl and the chloro in the quinoline ring and the benzoic acid to be the critical structural elements that contribute to MDM2-MDM4 E3 ligase inhibitory effects of MMRi67 in vitro. Whereas MMRi67 is not highly cytotoxic, its ethyl benzoate analogs, including MMRi71, were, and they exhibited a strong MDM2/MDM4 and FTH1 degradation in cells. MMRi71 represents a new class of dual inhibitors targeting the degradation of MDM2/MDM4 and FTH1 for p53-independent apoptosis in leukemia cells. Up to this point, our small molecule structural optimization efforts have focused on discerning and optimizing structural attributes that impact biochemical target interactions, the antiproliferation of cancer cells, and physical features related to compound formulation (optimizing LogP and discouraging metal chelation). Going forward, we will increase our analysis of factors related to in vivo efficacy and toxicity. Currently, we note that the lead compound MMRi71 has a molecular weight of 490.94 g/mol, contains one hydrogen bond donor, seven potential hydrogen-bond acceptor nitrogens and oxygens, and a LogP of 4.6. The calculated total polar surface area of MMRi71 is 101.71 Å^2^, and it has nine rotatable bonds. All of these properties conform to those recommended for orally available drugs [39,40]. Further lead optimization to improve the potency, pharmacokinetics, and pharmacodynamics profiles is warranted for developing clinically useful derivatives in this chemical domain.

## 5. Patents

Provisional patent: Antiproliferative Betti Bases and Prodrugs Thereof, pending.

## Figures and Tables

**Table 1:** **MMRi62** and **MMRi67** compound series IC_50_.

Entry	NALM6 IC_50_ (μM)	shP53NALM6 IC_50_ (μM)
**MMRi62**	0.12 ± 0.001	0.14 ± 0.001
**62-1**	0.29 ± 0.01	0.46 ± 0.04
**62-2**	32.4 ± 2.25	35.5 ± 2.62
**62-3**	8.58 ± 0.83	24.2 ± 4.38
**62-4**	4.84 ± 0.48	4.68 ± 0.52
**62-5**	2.62 ± 0.36	1.51 ± 0.12
**62-6**	1.89 ± 0.53	1.08 ± 0.08
**62-7**	0.35 ± 0.01	0.26 ± 0.02
**62-8**	28.6 ± 2.87	28.5 ± 3.82
**62-9**	0.30 ± 0.02	0.36 ± 0.03
**62-10** **62-11** **MMRi67** **67-1** **67-2** **67-3** **67-4** **67-5** **67-6** **67-7** **67-8** **67-9** **MMRi71**	12.0 ± 1.79 2.10 ± 0.22 **12.80 ± 1.57** 8.93 ± 0.41 0.40 ± 0.01 0.38 ± 0.03 0.53 ± 0.03 0.38 ± 0.03 >100 18.2 ± 6.54 2.13 ± 0.18 0.27 ± 0.02 **0.23 ± 0.01**	27.1 ± 5.58 2.66 ± 0.17 **10.89 ± 1.56** 10.1 ± 0.51 0.47 ± 0.01 0.48 ± 0.02 0.57 ± 0.04 0.45 ± 0.01 >100 31.7 ± 3.52 2.36 ± 0.15 0.37 ± 0.003 **0.29 ± 0.02**

The treatment was tri replicated and repeated two times. IC_50_ ± SD were performed using non-linear regression analysis.

## Data Availability

All data are included in the article and/or Supplementary Materials.

## Supplementary Materials

The following supporting information can be downloaded at: https://www.mdpi.com/article/10.3390/molecules27227665/s1, Experimental procedures and data for characterization of all new compounds, including NMR spectra, are provided in the chemistry supporting information [30,41,42,43,44,45,46,47,48,49].
