# Supplementary material for: Dual Targeting of MDM4 and FTH1 by MMRi71 for Induced Protein Degradation and p53-Independent Apoptosis in Leukemia Cells
Source: Molecules. 2022 Nov 8;27(22):7665. doi: 10.3390/molecules27227665 (PMC9695299; doi:10.3390/molecules27227665)
Supplement: Supplementary file 1 [file molecules-27-07665-s001.zip › molecules-1981371-supplementary.pdf]

Dual Targeting MDM4 and FTH1 by Small-Molecule MMRi71 for Induced Protein Degradation and p53-independent Apoptosis in Leukemia Cells

Rati Lama[1†], Samuel L. Galster[2†], Chao Xu[1], Luke W. Davison,[2] Sherry R. Chemler[2]* and Xinjiang Wang[1]*

Department of Pharmacology and Therapeutics, Roswell Park Comprehensive Cancer Center, Buffalo, NY 14263, USA

Department of Chemistry, University at Buffalo, State University of New York, Buffalo, NY 14260, USA

Table of Contents

Discussion of covalent inhibition mechanism	S-2
General Information	S-2
Synthesis and data for new analogs	S-4
References	S-28

Supporting Information

A. Discussion of potential covalent inhibition mechanism:

We have presented data that indicates that **MMRi62** and perhaps some **62**- and **67**-based analogs (including **MMRi71**), might function in part as covalent inhibitors. We envision covalent inhibition could occur, as previously proposed for related phenolic benzylic amines,[1-3] via a quinone methide forming reaction followed by addition of a protein's nucleophilic functionality (e.g. amine or thiol) to the resulting electrophile. Our further efforts to probe this mechanism are underway and will be reported in due course. Thus far, a covalent protein-**MMRi62** covalent adduct has not been isolated or biochemically identified. We did find **MMRi62** to be stable in C_2D_5OD at 100 °C for 24 h including with added CD_3CO_2D and deuterated pyridine, respectively (^1H NMR analysis, separate experiments), so if **MMRi62** and related analogs are covalent inhibitors, it is likely they selectively bind their targets and become activated to the quinone methide within the target.

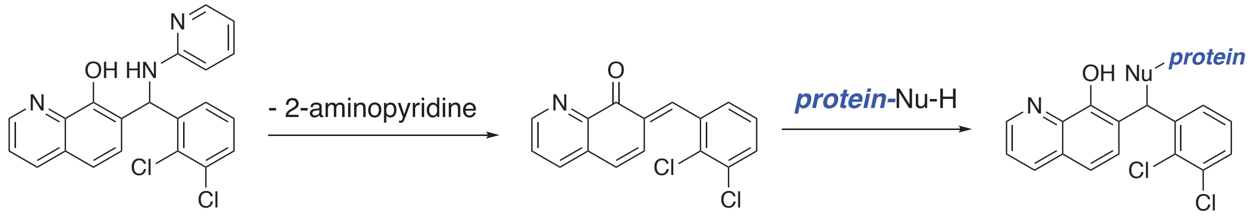

MMRi62 quinone methide covalent adduct

B. Chemistry Experimental Procedures and New Compound Characterization

General Information

All reagents were used out of the bottle as purchased from the supplier without further purification unless otherwise noted. ^1H NMR spectra were recorded in $CDCl_3$ (using 7.26 ppm for reference of $CHCl_3$), CD_2Cl_2 (using 5.30 ppm for reference of CH_2Cl_2) DMSO-d_6 (using 2.50 ppm for

reference of DMSO) at 300 or 400 MHz. ^{13}C NMR spectra were recorded in $CDCl_3$ (using 77.0 ppm as internal reference), CD_2Cl_2 (using 54.0 ppm as internal reference), or DMSO-d_6 (using 40.0 ppm as internal reference) at 75.5 or 101 MHz. IR spectra were taken neat using a Nicolet-Impact 420 FTIR. Wave numbers in cm^{-1} are reported for characteristic peaks. High resolution mass spectra were obtained at SUNY Buffalo's mass spec facility on a ThermoFinnigan MAT XL spectrometer. Melting points were obtained on an electrothermal melting point apparatus and are reported uncorrected. 2-Aminopyridine, 1,3-dichlorobenzaldehyde, 8-hydroxyquinoline, 1-napthol phenol, and 5-chloro-8-hydroxyquinoline were purchased from Acros and used without further purification. N-Phenyl-bis(trifluoromethanesulfonimide) was purchased from AK Scientific used without further purification. 4,5-Dichloropyridine-3-carbaldehyde was purchased from AABlocks and used without further purification. Analogs **67-2** and **67-3** were obtained as part of a compound screening library from Hit2lead Chembridge. Known analog **62-11** was synthesized as previously reported.[4-5] Analogs in the **62** and **67** series were synthesized via a 3-component Betti reactions.[4-6] Ethyl 4-formylbenzoate was synthesized as previously reported.[7]

Statement on compound purity: The purity of each **MMRi62** or **MMRi67** analog compound that was subjected to cellular and biochemical assays was assessed by each compound's ^{1}H NMR spectra, acquired at 300 or 400 MHz.[8] These spectra are included at the end of this SI. Based on these spectra, all compounds were at least 95% pure. In addition, the HPLC trace of **MMRi71** is provided and supports that is is >95% pure.

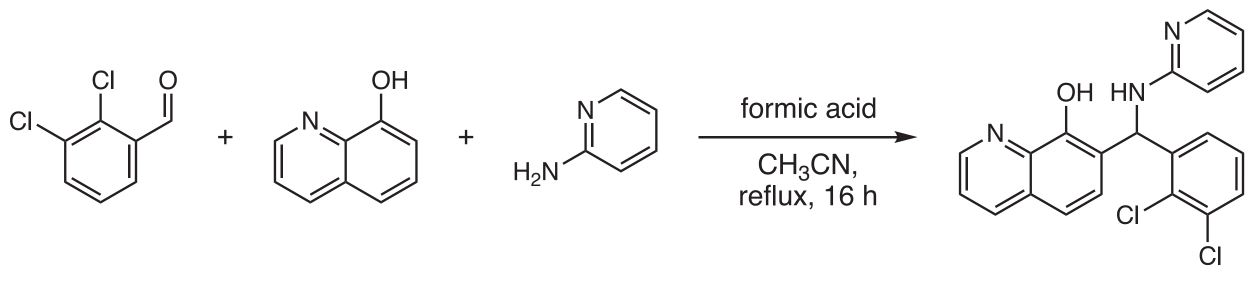

MMRi62, 21% yield

7-((2,3-Dichlorophenyl)(pyridin-2-ylamino)methyl)quinolin-8-ol (MMRi62)

To a dry 50 mL round-bottomed flask, 2-aminopyridine (269 mg, 2.86 mmol, 1.0 equiv.), 2,3-dichlorobenzaldehyde (500 mg, 2.86 mmol, 1.0 equiv.), and 8-hydroxyquinoline (500 mg, 3.43 mmol, 1.2 equiv) were dissolved in CH_3CN (30 mL). Following the addition of formic acid (86 μL, 2.29 mmol 0.80 eq), the solution was refluxed for 16 h. The solution was allowed to cool to rt, concentrated, and the crude mixture was then directly purified by flash column chromatography (silica gel, 10-20% acetone:hexanes) to give **MMRi62** as a white solid (236 mg, 21%). mp = 178-179 °C; ^{1}H NMR (300 MHz, $CDCl_3$) δ 8.76 (d, J = 4.1 Hz, 1H), 8.17 – 8.04 (m, 2H), 7.59 (d, J = 7.8 Hz, 1H), 7.47 – 7.34 (m, 4H), 7.29 (s, 1H), 7.19 (t, J = 7.9 Hz, 1H), 6.68 (d, J = 6.3 Hz, 1H), 6.61 (t, J = 6.1 Hz, 1H), 6.35 (d, J = 8.4 Hz, 1H), 5.55 (d, J = 6.4 Hz, 1H).; ^{13}C NMR (75 MHz, $CDCl_3$) δ 157.5, 149.8, 148.2, 148.1, 141.5, 138.2, 137.8, 136.0, 133.5, 132.0, 129.4, 127.8, 127.2, 127.1, 126.9, 122.0, 121.3, 117.7, 113.7, 106.7, 53.8.; IR neat film: 3351, 3079, 1599, 1571, 1516, 1502 cm^{-1}; HRMS (ESI) calculated for $[C_{21}H_{16}Cl_2N_3O]^+$: 396.0665, found 396.0649.

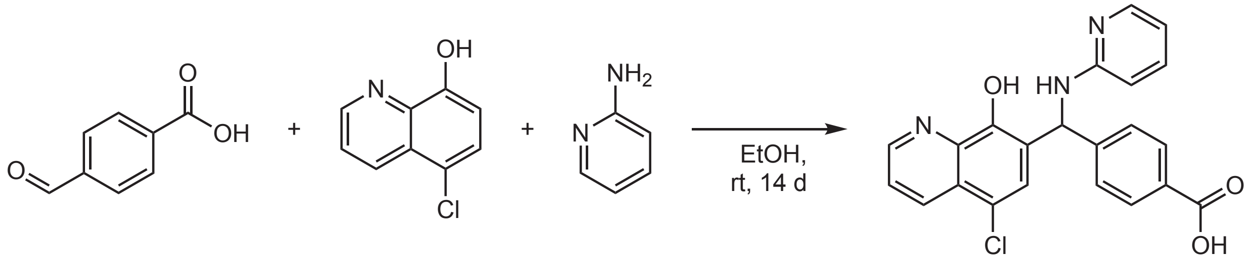

MMRi67, 11% yield

4-((5-Chloro-8-hydroxyquinolin-7-yl)(pyridin-2-ylamino)methyl)benzoic acid (MMRi67)

To a dry 10 mL round-bottomed flask, 2-aminopyridine (94.11 mg, 1.0 mmol, 1.0 equiv.) and 4-carboxybenzaldehyde (150.13 mg, 1.0 mmol, 1.0 equiv.) were dissolved in EtOH (5 mL). Then 5-chloro-8-hydroxyquinoline (179.60 mg, 1.0 mmol, 1.0 equiv) was added, the flask was capped and stirred at rt for 14 d. Upon appearance of a precipitate, the stirring was stopped and the solid allowed to settle. The solid was filtered and washed with HPLC grade hexanes to give **MMRi67** as an orange solid (45 mg, 11%). mp = 199-200 °C; ^{1}H NMR (400 MHz, DMSO-d6) δ 12.83 (bs, 1H), 10.40 (bs, 1H), 8.95 (s, 1H), 8.46 (d, J = 8.5 Hz, 1H), 7.89 (d, J = 6.2 Hz, 3H), 7.77 (s, 1H), 7.74 – 7.66 (m, 1H), 7.53 (d, J = 8.7 Hz, 1H), 7.48 (d, J = 6.3 Hz, 2H), 7.41 (t, J = 7.9 Hz, 1H), 6.96 (d, J = 8.4 Hz, 1H), 6.71 (d, J = 8.4 Hz, 1H), 6.51 (t, J = 6.3 Hz, 1H); ^{13}C NMR (101 MHz, DMSO-d6) δ 167.1, 157.6, 149.4, 149.2, 148.0, 147.4, 138.7, 136.9, 132.5, 129.5, 129.3, 127.3, 126.3, 126.0, 124.9, 122.9, 118.7, 112.5, 109.0, 51.2. IR neat film: 3281, 2953, 1672, 1603, 1576, 1504 cm^{-1}; HRMS (ESI) calculated for $[C_{22}H_{17}Cl_{1}N_{3}O_{3}]^{+}$: 406.0953, found 406.0964.

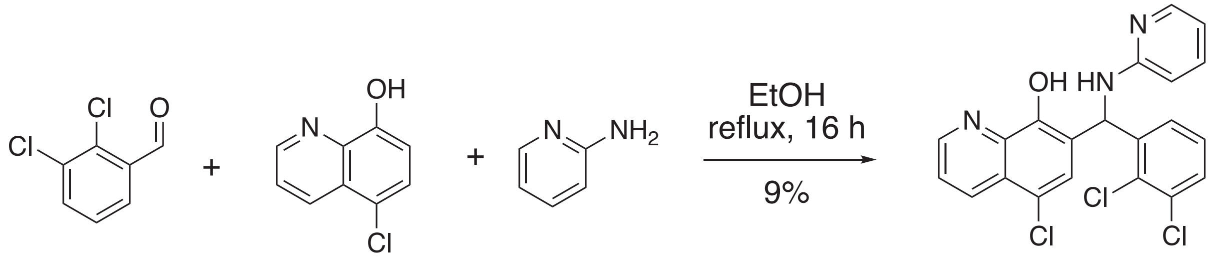

62-1

5-Chloro-7-((2,3-dichlorophenyl)(pyridin-2-ylamino)methyl)quinolin-8-ol (62-1)

In a 50 mL pressure tube, 2-aminopyridine (94 mg, 1 mmol, 1.0 equiv.), 2,3-dichlorobenzaldehyde (175 mg, 1 mmol, 1.0 equiv.), and 5-chloro-8-hydroxyquinoline (180 mg, 1 mmol, 1.0 equiv) were dissolved in absolute ethanol (5 mL). The tube was capped and heated to 80 °C for 16 h. Upon cooling to rt a precipitate formed. This solid was filtered and washed with Et_2O to yield pure **1-15** as a tan solid (40 mg, 9% yield). mp = 178-181 °C; ^{1}H NMR (300 MHz, $CDCl_3$) δ 8.82 (d, J = 4.3 Hz, 1H), 8.48 (d, J = 8.5 Hz, 1H), 8.09 (d, J = 5.0 Hz, 1H), 7.60 – 7.50 (m, 2H), 7.48 (s, 1H), 7.42 (q, J = 4.0 Hz, 2H), 7.21 (t, J = 7.9 Hz, 1H), 6.70 (d, J = 6.3 Hz, 1H), 6.63 (t, J = 6.2 Hz, 1H), 6.37 (d, J = 8.4 Hz, 1H), 5.48 (d, J = 6.3 Hz, 1H); ^{13}C NMR (75 MHz, $CDCl_3$) δ 157.2, 149.0, 148.7, 147.6, 140.8, 138.6, 138.1, 133.6, 133.3, 132.0, 129.7, 127.4, 126.8, 126.7, 125.8, 122.7, 121.8, 121.8, 120.6, 113.9, 107.1, 53.4; IR neat film: 1598, 1574, 1495 cm^{-1}; HRMS (ESI) calculated for $[C_{21}H_{15}Cl_3N_3O]^+$: 430.0275, found 430.0281.

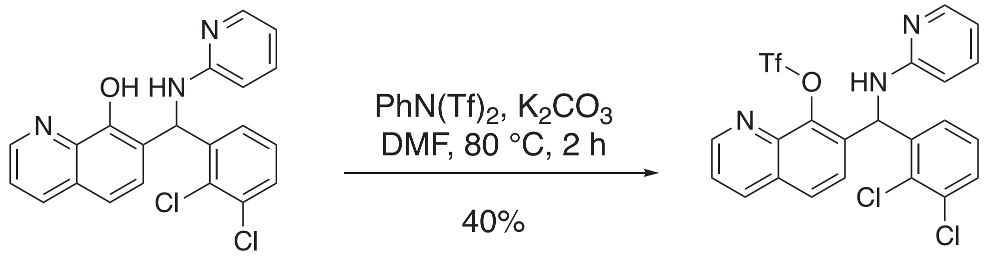

MMRi62 **S-1**

7-((2,3-Dichlorophenyl)(pyridin-2-ylamino)methyl)quinolin-8-yl trifluoromethanesulfonate (S-1)

In a 10 mL reaction tube, **MMRi62** (285 mg, 0.72 mmol, 1.0 equiv.) was dissolved in dry DMF (1 mL) under argon atmosphere. N-Phenyl-bis(trifluoromethanesulfonimide) (385 mg, 1.08 mmol,

1.5 equiv.) and potassium carbonate (150 mg, 1.09 mmol, 1.5 equiv.) were added, the tube was capped and the solution was heated in an 80 °C oil bath for 2 h. The solution was then cooled to rt, diluted with EtOAc, and washed three times with H_2O. The organic layer was dried over Na_2SO_4, and then concentrated. The crude mixture was purified by flash column chromatography (silica gel, 10% acetone/hexanes gradient) to yield **S-1** as a white solid (152 mg, 40% yield). mp = 201-203 °C; ^{1}H NMR (400 MHz, $CDCl_3$) δ 9.04 (d, *J* = 4.2 Hz, 1H), 8.19 (d, *J* = 8.4 Hz, 1H), 8.06 (d, *J* = 5.1 Hz, 1H), 7.78 (d, *J* = 8.6 Hz, 1H), 7.54 (dd, *J* = 8.4, 4.2 Hz, 1H), 7.49 – 7.32 (m, 4H), 7.19 (t, *J* = 7.9 Hz, 1H), 6.69 (d, *J* = 5.2 Hz, 1H), 6.65 (t, 1H), 6.32 (d, *J* = 8.4 Hz, 1H), 5.16 (d, *J* = 5.2 Hz, 1H); ^{13}C NMR (101 MHz, $CDCl_3$) δ 156.9 151.3, 148.3, 144.3, 140.5, 140.0, 137.7, 135.6, 134.0, 132.6, 132.3, 130.1, 129.1, 127.8, 127.4, 126.8, 125.8, 122.8, 120.4, 117.2, 114.4, 106.8, 53.2; IR neat film: 3373, 2924, 2853, 2323, 2050, 1715, 1600, 1574 cm^{-1}; HRMS (ESI) calculated for $[C_{22}H_{15}Cl_2F_3N_3O_3S]^+$: 528.0158, found 528.0179.

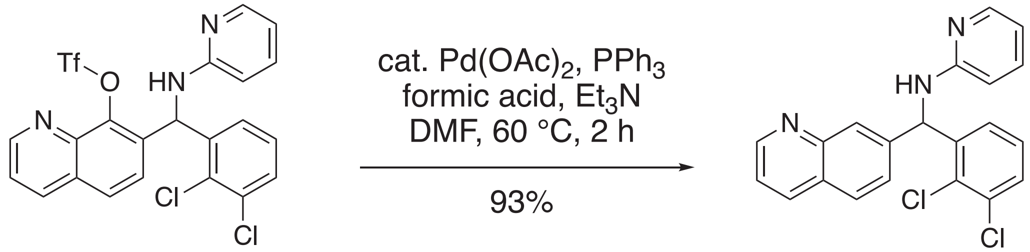

S-1 **62-2**

***N*-((2,3-Dichlorophenyl)(quinolin-7-yl)methyl)pyridin-2-amine (62-2)**

In a 10 mL pressure tube, **S-1** (90 mg, 0.17 mmol, 1.0 equiv), palladium acetate (4 mg, 0.017 mmol, 0.1 equiv), triphenylphosphine (9 mg, 0.034 mmol, 0.2 equiv), and triethylamine (71 µL, 0.51 mmol, 3.0 equiv) were combined in dry DMF (1 mL) under argon atmosphere.[9] Formic acid (13 µL, 0.34 mmol, 2.0 equiv) was added, and the reaction was capped and stirred at 60 °C for 2

h. Upon cooling to rt, the reaction mixture was diluted with brine and extracted with EtOAc. The organic layer was then washed three times with brine. The organic layer was dried over Na_2SO_4 and then concentrated. The crude mixture was purified by flash column chromatography (silica gel, 30% EtOAc:hexanes gradient) to yield **62-2** as a white solid (60 mg, 93% yield). mp = 220-221 °C; ^{1}H NMR (300 MHz, $CDCl_3$) δ 8.94 (d, *J* = 4.2 Hz, 1H), 8.37 (d, *J* = 8.2 Hz, 1H), 8.08 (d, *J* = 8.4 Hz, 2H), 7.64 – 7.55 (m, 1H), 7.50 – 7.35 (m, 4H), 7.25 – 7.18 (m, 2H), 7.01 (d, *J* = 6.6 Hz, 1H), 6.69 – 6.61 (m, 1H), 6.33 (d, *J* = 8.8 Hz, 1H), 5.00 (d, *J* = 6.6 Hz, 1H).; ^{13}C NMR (75 MHz, $CDCl_3$) δ 156.5, 151.3, 144.3, 140.5, 139.8, 138.3, 135.7, 134.0, 132.3, 130.2, 129.1, 127.9, 127.5, 126.8, 125.8, 122.8, 114.3, 107.1, 53.1; IR neat film: 3254, 3062, 3018, 2924, 2854, 2114, 1730, 1671, 1601, 1574, 1501 cm^{-1}; HRMS (ESI) calculated for $[C_{21}H_{16}Cl_2F_3N_3]^+$: 380.0716, found 380.0714.

.

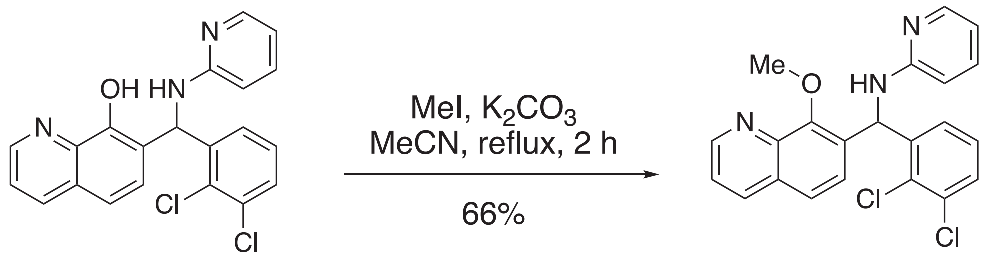

MMRi62 **62-3**

***N*-((2,3-Dichlorophenyl)(8-methoxyquinolin-7-yl)methyl)pyridin-2-amine (62-3)**

In a 10 mL pressure tube, **MMRi62** (50 mg, 0.13 mmol, 1.0 equiv) was dissolved in anhydrous acetonitrile (1 mL) under argon atmosphere. Potassium carbonate (20 mg, 0.14 mmol, 1.1 equiv), followed by methyl iodide (9 µL, 0.14 mmol, 1.1 equiv) were added to the solution. The mixture was stirred at reflux temperature for 2 h. Upon cooling to rt, the reaction mixture was diluted with ethyl acetate and washed with water. The organic layer was dried over sodium sulfate and then concentrated. The crude mixture was purified by flash column chromatography (silica gel, 10%-

20% acetone/hexanes gradient) to yield **62-3** as a white solid (33 mg, 66% yield). mp =122-123 °C; ^{1}H NMR (300 MHz, $CDCl_3$) δ 8.93 (d, *J* = 4.6 Hz, 1H), 8.13 (d, *J* = 8.2 Hz, 1H), 8.07 (d, *J* = 4.9 Hz, 1H), 7.55 – 7.35 (m, 8H), 7.19 (t, *J* = 7.8 Hz, 1H), 6.77 (d, *J* = 6.4 Hz, 1H), 6.61 (t, *J* = 6.2 Hz, 1H), 6.34 (d, *J* = 8.4 Hz, 1H), 5.24 (d, *J* = 6.4 Hz, 1H), 4.05 (s, 1H); ^{13}C NMR (75 MHz, $CDCl_3$) δ 157.4, 154.1, 149.6, 148.3, 142.8, 141.9, 137.7, 136.2, 133.6, 132.4, 132.0, 129.48, 129.45, 127.2, 127.0, 126.3, 123.2, 121.4, 113.8, 106.7, 62.5, 53.8; IR neat film: 3266, 3097, 3019, 1603, 1520, 1503 cm^{-1}; HRMS (ESI) calculated for $[C_{22}H_{18}Cl_2N_3O]^+$: 410.0821, found 410.0814.

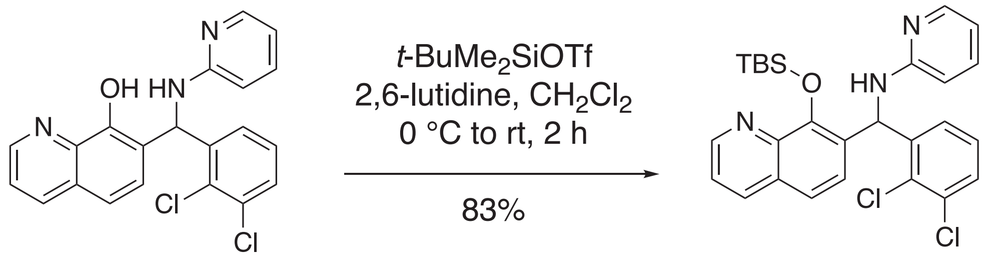

MMRi62 **S-2**

***N*-((8-((*tert*-Butyldimethylsilyl)oxy)quinolin-7-yl)(2,3-dichlorophenyl)methyl)pyridin-2-amine (S-2)**

In a 25 mL round-bottom flask, **MMRi62** (100 mg, 0.128 mmol, 1.0 equiv), and 2,6-lutidine (75 μL, 0.630 mmol, 5.0 equiv) were dissolved in CH_2Cl_2 (10 mL) under argon atmosphere at 0 °C. TBSOTf (0.15 mL, 0.630 mmol, 5.0 equiv) was added dropwise and allowed to stir for 1 h at 0 °C. The reaction was slowly warmed to rt and allowed to stir for an additional 2 h. The reaction mixture was quenched with aqueous $NaHCO_3$ and extracted 3 times with CH_2Cl_2. The organic layers were combined and dried over Na_2SO_4. The organic solution was concentrated and purified by flash column chromatography (silica gel, 10% EtOAc:hexanes gradient) to yield **S-2** as a pale yellow solid (107 mg, 83% yield). mp = 74-77 °C; ^{1}H NMR (300 MHz, $CDCl_3$) δ 8.79 (dd, *J* = 4.1, 1.6 Hz, 1H), 8.11 – 8.01 (m, 2H), 7.54 (dd, *J* = 7.6, 1.3 Hz, 1H), 7.45 – 7.33 (m, 3H), 7.27 (d, *J* = 8.4 Hz, 1H), 7.20 (t, *J* = 7.9 Hz, 1H), 7.13 (d, *J* = 8.5 Hz, 1H), 6.72 (d, *J* = 4.9 Hz, 1H), 6.66 – 6.55 (m, 1H), 6.23 (d, *J* = 8.4 Hz, 1H), 5.21 (d, *J* = 4.7 Hz, 1H), 0.89 (s, 9H), 0.36 (d, *J* = 3.0 Hz, 6H); ^{13}C NMR (75 MHz, $CDCl_3$) δ 157.7, 151.2, 148.0, 147.1, 141.8, 140.3, 137.7, 135.6, 133.7, 132.1, 129.4, 129.0, 127.2, 126.7, 126.4, 121.5, 119.1, 113.7, 106.6, 53.4, 26.2, 19.3, -1.8, -2.1; IR neat film: 3217, 2984, 2927, 2854, 1598, 1573, 1502 cm^{-1}; HRMS (ESI) calculated for $[C_{27}H_{30}Cl_2N_3OSi]^+$: 510.1524, found 510.1530.

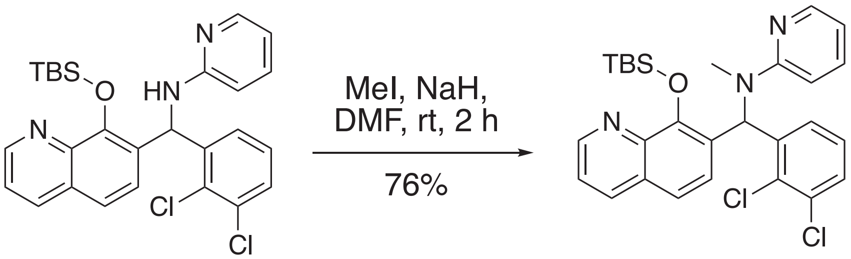

S-2 **S-3**

***N*-((8-((*tert*-Butyldimethylsilyl)oxy)quinolin-7-yl)(2,3-dichlorophenyl)methyl)-*N*-methylpyridin-2-amine (S-3)**

In a 5 mL round-bottom flask, intermediate **S-2** (107 mg, 0.209 mmol, 1.0 equiv) was dissolved in dry DMF (2.5 mL). Once dissolved, NaH (50 mg, 2.09 mmol, 10.0 equiv) was added and stirred for 15 min at rt. After 15 min, CH_3I (0.13 mL, 2.09 mmol, 10.0 equiv) was added to the solution and allowed to stir for an additional 2 h at rt. The solution was then diluted with EtOAc (10 mL), and washed 3 times with H_2O (10 mL). The organic layer was then dried over Na_2SO_4 and concentrated. The crude mixture was then purified by flash column chromatography (silica gel, 10% EtOAc:hexanes gradient) to yield intermediate **S-3** as a white solid (84 mg, 76% yield). mp = 59-61 °C; ^{1}H NMR (300 MHz, $CDCl_3$) δ 8.79 (dd, *J* = 4.1, 1.6 Hz, 1H), 8.19 (dd, *J* = 4.9, 1.2 Hz, 1H), 8.09 (dd, *J* = 8.3, 1.6 Hz, 1H), 7.46 – 7.33 (m, 4H), 7.31 (s, 1H), 7.20 (s, 1H), 7.15 – 7.07 (m, 2H), 7.01 (s, 1H), 6.56 (dd, *J* = 7.0, 5.0 Hz, 1H), 6.46 (d, *J* = 8.6 Hz, 1H), 2.97 (s, 3H), 0.80 (s, 9H), 0.36 (s, 3H), 0.26 (s, 3H); ^{13}C NMR (75 MHz, $CDCl_3$) δ 158.4, 151.4, 147.8, 146.9, 141.6, 140.3, 137.0, 135.6, 133.8, 132.8, 129.3, 129.0, 127.6, 127.4, 127.0, 126.4, 121.3, 118.8, 112.2, 106.3, 58.9, 33.0, 26.0, 19.0, -1.8, -2.0; IR neat film: 2949, 2927, 2893, 2855, 1594, 1561, 1502 cm^{-1}; HRMS (ESI) calculated for $[C_{28}H_{32}Cl_2N_3OSi]^+$: 524.1699, found 524.1686.

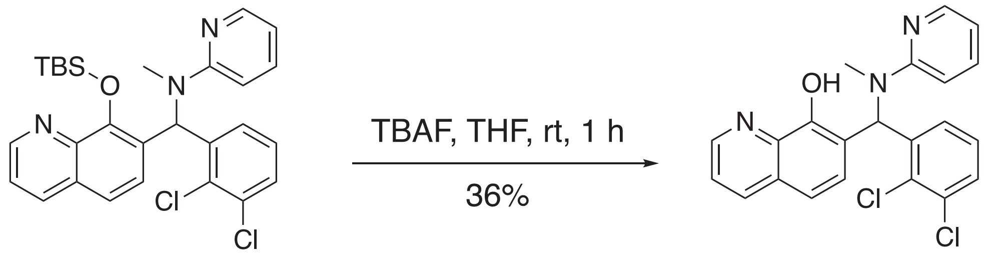

7-((2,3-Dichlorophenyl)(methyl(pyridin-2-yl)amino)methyl)quinolin-8-ol (62-4)

In a 10 mL round-bottomed flask, intermediate **S-3** (84 mg, 0.160 mmol, 1 equiv) was dissolved in dry THF (1 mL) under argon atmosphere at 0 °C. A 1 M solution of TBAF in THF (0.25 mL, 0.240 mmol, 1.5 equiv) was added dropwise at 0 °C and stirred for 1 h. The solution was slowly warmed to rt and stirred for 3 h. The reaction mixture was concentrated and dissolved in EtOAc. The solution was washed with saturated NH_4Cl. The organic layer was dried over Na_2SO_4 and concentrated. The crude mixture was purified by flash column chromatography (silica gel, 10% acetone:hexanes gradient) to yield **62-4** as a white solid (24 mg, 37% yield). mp = 169-170 °C; ^{1}H NMR (400 MHz, $CDCl_3$) δ 8.79 (d, *J* = 2.7 Hz, 1H), 8.24 (d, *J* = 3.7 Hz, 1H), 8.16 (d, *J* = 8.2 Hz, 1H), 7.53 – 7.42 (m, 3H), 7.30 (t, *J* = 7.9 Hz, 1H), 7.22 – 7.10 (m, 3H), 6.69 – 6.53 (m, 2H), 2.99 (s, 3H); ^{13}C NMR (101 MHz, $CDCl_3$) δ 158.5, 150.2, 148.3, 147.9, 141.0, 138.0, 137.3, 136.0, 133.7, 132.6, 129.4, 127.9, 127.5, 127.4, 127.1, 121.9, 120.7, 117.5, 112.4, 106.1, 58.3, 33.1; IR neat film: 3242, 2923, 1593, 1557, 1506 cm^{-1}; HRMS (ESI) calculated for $[C_{22}H_{18}Cl_2N_3O]^+$: 410.0834, found 410.0821.

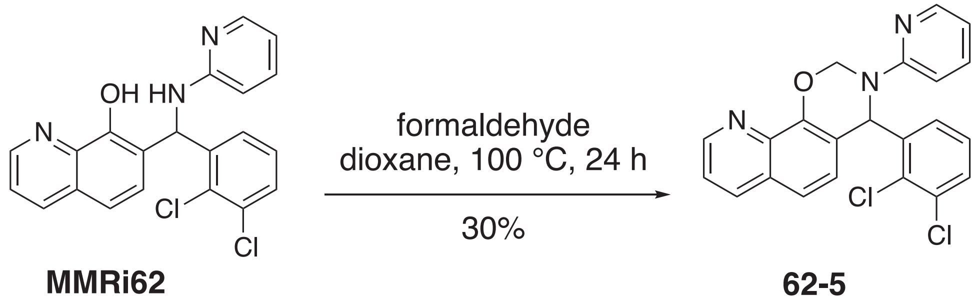

4-(2,3-Dichlorophenyl)-3-(pyridin-2-yl)-3,4-dihydro-2*H*-[1,3]oxazino[5,6-*h*]quinolone (62-5)

To a 10 mL pressure tube, **MMRi62** (100 mg, 0.25 mmol, 1.0 equiv) and paraformaldehyde (8.5 mg, 0.28 mmol, 1.1 equiv) were dissolved in dioxane (1.5 mL).[10] The solution was heated to 100 °C for 24 h. Upon cooling to rt, the solution was diluted with Et_2O, and concentrated. The crude mixture was purified by flash column chromatography (silica gel, 20-30% acetone:hexanes gradient) to give **62-5** as a green-white solid (30 mg, 30%). mp = 115-117 °C; ^1H NMR (400 MHz, $CDCl_3$) δ 8.90 (d, *J* = 2.7 Hz, 1H), 8.25 (d, *J* = 4.8 Hz, 1H), 8.10 (d, *J* = 10.0 Hz, 1H), 7.56 (t, *J* = 6.9 Hz, 1H), 7.46 – 7.35 (m, 4H), 7.33 (s, 2H), 7.21 (d, *J* = 8.4 Hz, 1H), 7.13 – 7.07 (m, 3H), 6.95 (d, *J* = 7.7 Hz, 1H), 6.82 – 6.74 (m, 1H), 6.14 (d, *J* = 12.8 Hz, 1H), 5.22 (d, *J* = 11.0 Hz, 1H); ^{13}C NMR (75 MHz, $CDCl_3$) δ 157.1, 150.3, 149.7, 147.9, 142.2, 139.7, 137.9, 136.0, 134.1, 133.3, 130.3, 129.9, 128.6, 127.0, 126.6, 121.8, 119.5, 119.2, 116.4, 110.1, 73.9, 54.2; IR neat film: 2957, 1736, 1592, 1568, 1503 cm^{-1}; HRMS (ESI) calculated for $[C_{22}H_{16}Cl_2N_3O]^+$: 408.0682, found 408.0665.

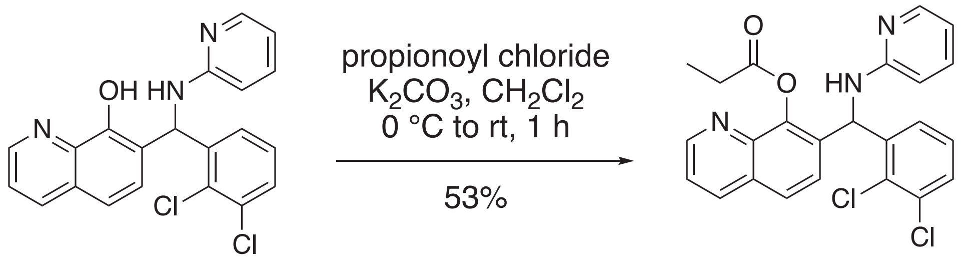

MMRi62 **62-6**

7-((2,3-Dichlorophenyl)(pyridin-2-ylamino)methyl)quinolin-8-yl propionate (62-6)

In a 250 mL dry round bottomed flask, **MMRi62** (3 g, 7.6 mmol, 1.0 equiv) was dissolved in 70 mL of dry CH_2Cl_2 under argon atmosphere. Potassium carbonate (2.0 g, 14.5 mmol, 2.0 equiv) was added and the solution was cooled to 0 °C. Propionoyl chloride (0.67 mL, 7.7 mmol, 1.0 equiv) was then added to the solution. The mixture was allowed to warm to rt and stirred for 1 h. The reaction mixture was then filtered through celite and washed with CH_2Cl_2. The supernatant was then treated with 800 mg DMT-functionalized silica gel and stirred for 15 min. The mixture was filtered and concentrated. The resulting crude solid was resuspended in ether and washed with deionized water. The organic layer was dried over Na_2SO_4 and then concentrated. The crude mixture was purified by flash column chromatography (silica gel, 50-100% ether:hexanes gradient) to yield **62-6** as a white solid (1.8 g, 53% yield). mp = 110-111 °C; ^{1}H NMR (300 MHz, $CDCl_3$) δ 8.92 (d, *J* = 4.2 Hz, 1H), 8.35 (d, *J* = 8.6 Hz, 1H), 8.10 (d, *J* = 5.2 Hz, 1H), 7.48 – 7.39 (m, 3H), 7.34 (dd, *J* = 16.3, 7.8 Hz, 2H), 7.24 – 7.12 (m, 2H), 7.00 (d, *J* = 6.5 Hz, 1H), 6.68 – 6.61 (m, 1H), 6.33 (d, *J* = 8.3 Hz, 1H), 5.03 (d, *J* = 6.5 Hz, 1H), 2.83 (q, *J* = 7.5 Hz, 2H), 1.35 (t, *J* = 7.5 Hz, 3H); ^{13}C NMR (75 MHz, $CDCl_3$) δ 173.3, 156.7, 150.3, 148.4, 147.7, 141.7, 141.2, 137.8, 134.7, 133.8, 132.1, 132.1, 129.8, 127.8, 127.5, 127.0, 125.1, 122.1, 120.7, 114.1, 107.1, 53.7, 27.6, 9.2; IR (neat, thin film): 3384, 2925 2051, 1760, 1599, 1574, 1502, 1479, 1147, 1081, 898, 771 cm^{-1}; HRMS (ESI) calculated for $[C_{24}H_{20}Cl_2N_3O_2]$, $(M+H)^+$: 452.0900, found 408.0928.

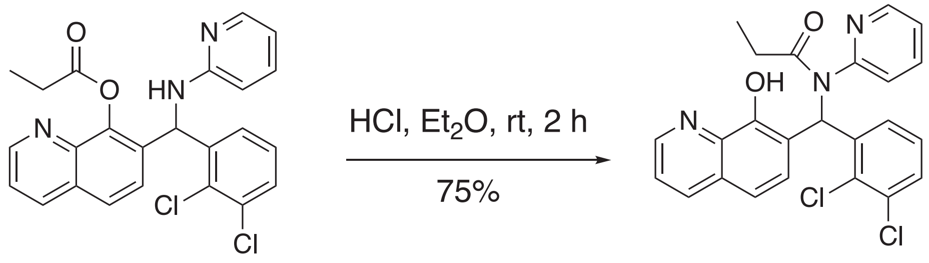

62-6 **62-7**

***N*-((2,3-Dichlorophenyl)(8-hydroxyquinolin-7-yl)methyl)-N-(pyridin-2-yl)propionamide (62-7)**

In a dry 10 mL dry round bottomed flask, analog **62-6** (20 mg, 0.041 mmol, 1.0 equiv) was dissolved in dry Et_2O (1 mL). Hydrochloric acid (2M in ether, 0.1 mL, 0.2 mmol, 4.4 equiv) was then added, and the reaction was stirred for 2 h at rt. The solution was then concentrated and the crude mixture was purified by flash column chromatography (silica gel, 50% ethyl aceate:hexanes) to yield **62-7** as a white solid (15 mg, 75% yield). mp = 175-176 °C; ^{1}H NMR (300 MHz, $CDCl_3$) δ 9.89 (s, 1H), 8.73 (d, *J* = 4.3 Hz, 1H), 8.11 (d, *J* = 8.7 Hz, 1H), 7.99 (d, *J* = 3.4 Hz, 1H), 7.58 (d, *J* = 7.8 Hz, 1H), 7.50 – 7.36 (m, 4H), 7.29 (d, *J* = 8.6 Hz, 1H), 7.20 (s, 1H), 7.11 – 7.01 (m, 1H), 6.69 (d, *J* = 3.8 Hz, 1H), 6.64 – 6.57 (m, 1H), 6.44 (d, *J* = 8.5 Hz, 1H), 2.30 (q, *J* = 7.5 Hz, 2H), 1.08 (t, *J* = 7.5 Hz, 2H); ^{13}C NMR (101 MHz, $CDCl_3$) δ 173.0, 155.0, 151.1, 145.7, 141.2, 139.0, 135.9, 133.7, 131.9, 131.6, 130.4, 130.2, 129.2, 128.0, 127.9, 127.7, 126.3, 124.6, 122.1, 113.3, 109.1, 53.5, 27.4, 9.2, 9.0; IR (neat, thin film): 3352, 2981, 1599, 1572 cm^{-1}; HRMS (ESI) calculated for $[C_{24}H_{20}Cl_2N_2N_3O_2]^+$: 452.0927, found 452.0932.

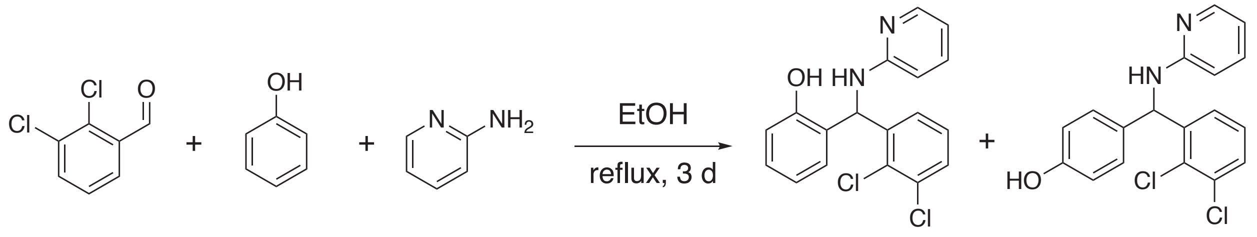

62-8
(24%)

S-4
(25%)

2-((2,3-Dichlorophenyl)(pyridin-2-ylamino)methyl)phenol (62-8)

To a 25 mL round-bottomed flask, 2-aminopyridine (75 mg, 0.796 mmol, 0.93 equiv) and 2,3-dichlorobenzaldehyde (150 mg, 0.857 mmol, 1.0 equiv) was dissolved in absolute ethanol (5 mL). Once fully dissolved, phenol (150 mg, 1.59 mmol, 1.9 equiv) was added to the solution. The mixture was stirred at reflux for 72 h. The reaction was allowed to cool and then concentrated. The crude mixture was purified by flash column chromatography (silica gel, 10-20% acetone:hexanes) to yield **62-8** as a white solid (70 mg, 24% yield), and **S-4** as a white solid (73 mg, 25% yield).

mp = 187-188 °C; ^{1}H NMR (300 MHz, $CDCl_3$) δ 11.30 (s, 1H), 8.08 (dd, J = 5.2, 0.9 Hz, 2H), 7.68 (d, J = 7.6 Hz, 2H), 7.54 – 7.43 (m, 2H), 7.38 (dd, J = 8.0, 0.9 Hz, 2H), 7.19 (t, J = 7.9 Hz, 2H), 7.15 – 7.06 (m, 4H), 6.87 (dd, J = 8.2, 0.7 Hz, 2H), 6.77 (t, J = 7.5 Hz, 2H), 6.72 – 6.63 (m, 2H), 6.46 (t, J = 7.9 Hz, 4H), 6.31 (d, J = 8.2 Hz, 2H); ^{13}C NMR (75 MHz, $CDCl_3$) δ 157.0, 155.8, 146.3, 141.3, 139.0, 133.4, 131.7, 129.5, 128.9, 127.2, 126.4, 126.0, 119.7, 117.1, 113.9, 108.4, 54.8; IR neat film: 3431, 3024, 2981, 2921, 2852, 2686, 2586, 1613, 1598, 1574 cm^{-1}; HRMS (ESI) calculated for $[C_{18}H_{15}Cl_2N_2O]^+$: 345.0558, found 345.055

4-((2,3-Dichlorophenyl)(pyridin-2-ylamino)methyl)phenol (S-4)

mp = 195-196 °C; ^{1}H NMR (300 MHz, $CDCl_3$) δ 8.07 (d, J = 5.2 Hz, 1H), 7.53 – 7.38 (m, 3H), 7.21 (t, J = 7.9 Hz, 1H), 7.01 (d, J = 8.4 Hz, 2H), 6.68 (dd, J = 7.2, 5.2 Hz, 1H), 6.19 (d, J = 8.3 Hz, 1H), 5.94 (d, J = 4.5 Hz, 1H), 5.32 (s, 1H); ^{13}C NMR (75 MHz, $CDCl_3$) δ 156.4, 146.0, 141.1,

139.4, 133.7, 131.5, 130.6, 129.6, 129.2, 127.6, 126.1, 118.6, 116.0, 113.8, 107.0, 58.3; IR neat film: 3433, 3413, 2981, 2799, 2671, 2590, 1604, 1571, 1514, 1502 cm^{-1}; HRMS (ESI) calculated for $[C_{18}H_{15}Cl_2N_2O]^+$: 345.0558, found 345.0556.

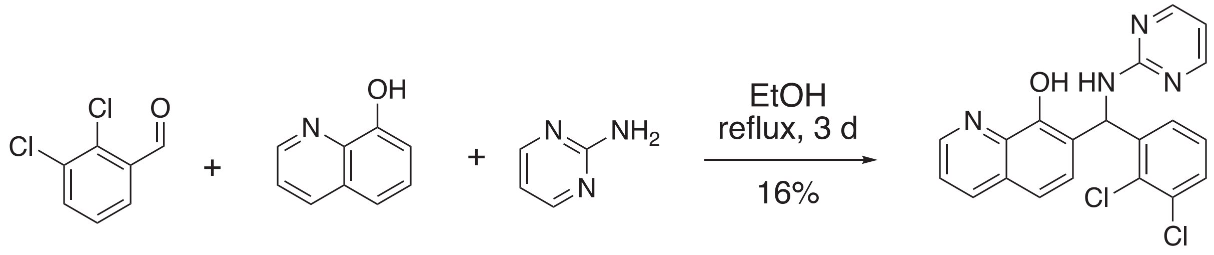

62-9

7-((2,3-Dichlorophenyl)(pyrimidin-2-ylamino)methyl)quinolin-8-ol (62-9)

To a 50 mL pressure tube, 2-aminopyrimidine (54.6 mg, 0.57 mmol, 1.0 equiv) and 2,3-dichlorobenzaldehyde (100 mg, 0.57 mmol, 1.0 equiv) was dissolved in absolute ethanol (5 mL). Once fully dissolved, 8-hydroxyquinoline (100 mg, 0.69 mmol, 1.2 equiv) was added to the solution. The mixture was stirred at reflux for 72 h. The reaction was allowed to cool to rt and then concentrated. The crude mixture was purified by flash column chromatography (silica gel, 10%-20% acetone:hexanes gradient) to yield **62-9** as a white solid (37.5 mg, 16% yield). mp = 98-99 °C ;^{1}H NMR (300 MHz, $CDCl_3$) δ 8.73 (d, J = 5.7 Hz, 1H), 8.25 (d, J = 4.8 Hz, 2H), 8.11 (d, J = 8.3 Hz, 1H), 7.60 (d, J = 7.8 Hz, 1H), 7.46 – 7.34 (m, 3H), 7.29 (s, 1H), 7.18 (t, J = 7.8 Hz, 1H), 7.04 (d, J = 7.7 Hz, 1H), 6.53 (t, J = 4.8 Hz, 1H), 6.35 (d, J = 7.7 Hz, 1H); ^{13}C NMR (75 MHz, $CDCl_3$) δ 161.3, 158.1, 149.8, 148.1, 141.9, 138.2, 136.0, 133.4, 131.9, 129.2, 127.8, 127.5, 126.9, 126.6, 121.9, 121.4, 117.5, 111.3, 53.4; IR; HRMS (ESI) calculated for $[C_{20}H_{15}Cl_2N_4O_1]^+$: 397.0617, found 397.0619.

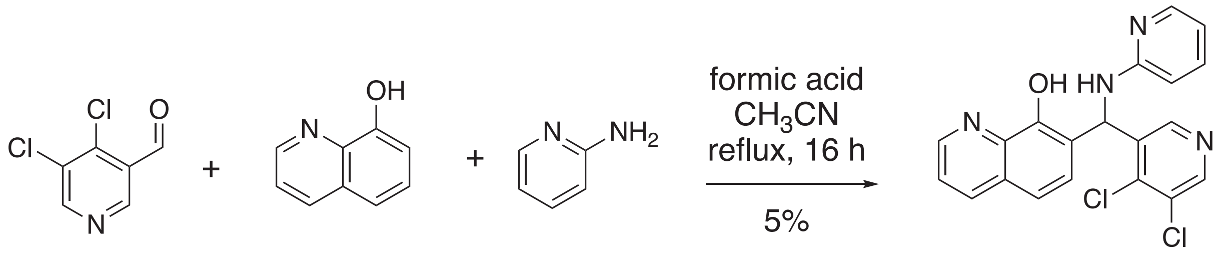

62-10

7-((4,5-Dichloropyridin-3-yl)(pyridin-2-ylamino)methyl)quinolin-8-ol (62-10)

To a 50 mL pressure tube, 2-aminopyridine (27 mg, 0.29 mmol, 1.0 equiv), 4,5-dichloronicotinaldehyde (50 mg, 0.29 mmol, 1.0 equiv.), and 8-hydroxyquinoline (50 mg, 0.34 mmol, 1.2 equiv) were dissolved in CH_3CN (30 mL). Following the addition of formic acid (10 μL, 0.23 mmol 0.80 equiv.), the solution was stirred at reflux for 16 h. The solution was allowed to cool to rt, concentrated, and the crude mixture was then directly purified by flash column chromatography (silica gel, 10-20% acetone:hexanes) to give **62-10** as a white solid (6 mg, 5% yield). mp = 102-103 °C; ^{1}H NMR (400 MHz, $CDCl_3$) δ 8.83 – 8.67 (m, 2H), 8.52 (s, 1H), 8.13 (d, J = 8.3 Hz, 1H), 8.08 (d, J = 5.0 Hz, 1H), 7.55 – 7.34 (m, 3H), 7.31 (d, J = 8.5 Hz, 1H), 6.74 (d, J = 6.6 Hz, 1H), 6.61 (t, J = 7.2 Hz, 1H), 6.41 (d, J = 8.3 Hz, 1H), 5.53 (d, J = 6.5 Hz, 1H); ^{13}C NMR (75 MHz, $CDCl_3$) δ 158.0, 149.7, 148.2, 148.1, 147.0, 143.5, 139.5, 138.0, 136.0, 127.8, 127.4, 124.9, 118.9, 118.1, 117.7, 117.6, 115.0, 113.0, 106.7, 53.2; HRMS (ESI) calc'd for $[C_{20}H_{15}Cl_2N_4O]$, $(M+H)^+$: 397.0623, found: 397.0618.

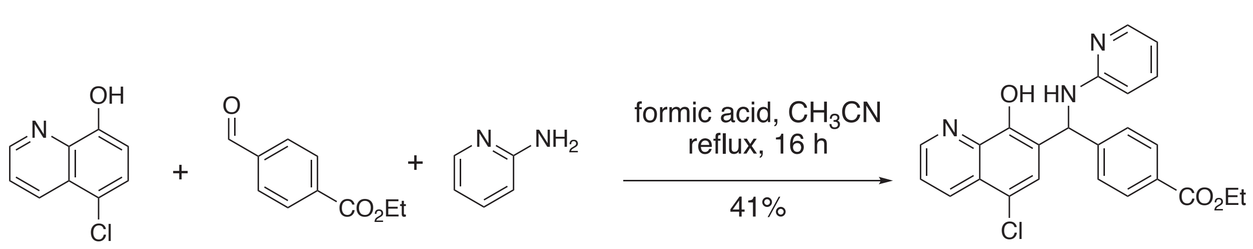

67-4

Ethyl 4-((5-chloro-8-hydroxyquinolin-7-yl)(pyridin-2-ylamino)methyl)benzoate (67-4)

To a 50 mL pressure tube, 2-aminopyridine (26 mg, 0.28 mmol, 1.0 equiv) ethyl 4-formylbenzoate (50 mg, 0.28 mmol, 1.0 equiv.), and 5-chloro-8-hydroxyquinoline (60 mg, 0.34 mmol, 1.2 equiv.) were dissolved in CH_3CN (3 mL). Following the addition of formic acid (10 µL, 0.28 mmol 1.0 equiv.), tube was capped and the solution was stirred at reflux for 16 h. The solution was allowed to cool to rt, concentrated, and the crude mixture was then directly purified by flash column chromatography (silica gel, 10-50% acetone:hexanes) to give **67-4** as a white solid (50 mg, 41% yield). mp = 50-51 C; ^{1}H NMR (300 MHz, $CDCl_3$) δ 8.81 (d, J = 4.3 Hz, 1H), 8.47 (d, J = 8.6 Hz, 1H), 8.10 (d, J = 6.8 Hz, 1H), 8.00 (d, J = 8.1 Hz, 2H), 7.61 (s, 1H), 7.54 (d, J = 8.3 Hz, 3H), 7.39 (t, J = 8.7 Hz, 1H), 6.65 – 6.59 (m, 1H), 6.51 (d, J = 6.5 Hz, 1H), 6.43 (d, J = 8.4 Hz, 1H), 5.59 (d, J = 6.6 Hz, 1H), 4.35 (q, J = 7.1 Hz, 2H), 1.36 (t, J = 7.1 Hz, 3H).; ^{13}C NMR (75 MHz, $CDCl_3$) δ 166.3, 157.5, 148.8, 148.4, 148.0, 146.5, 138.8, 137.8, 133.3, 130.0, 129.7, 127.0, 126.4, 125.7, 123.8, 122.6, 121.0, 113.9, 107.4, 60.9, 54.8, 14.31. IR neat film: 3292, 2977, 1717, 1603, 1574, 1509 cm^{-1} HRMS (ESI) calculated for $[C_{24}H_{21}ClN_3O_3]^+$: 434.1278, found 434.1279.

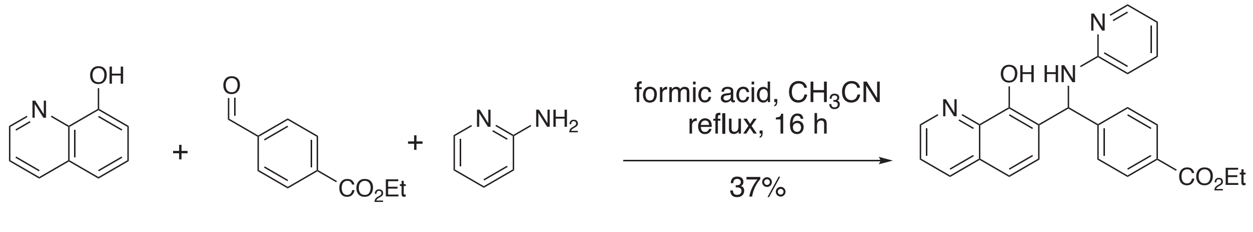

67-5

Ethyl 4-((8-hydroxyquinolin-7-yl)(pyridin-2-ylamino)methyl)benzoate (67-5)

To a 50 mL pressure tube, 2-aminopyridine (26 mg, 0.28 mmol, 1.0 equiv) ethyl 4-formylbenzoate (50 mg, 0.29 mmol, 1.0 equiv.), and 8-hydroxyquinoline (50 mg, 0.34 mmol, 1.2 equiv.) were dissolved in CH_3CN (30 mL). Following the addition of formic acid (10 µL, 0.23 mmol 0.80

equiv.), the solution was stirred at reflux for 16 h. The solution was allowed to cool to rt, concentrated, and the crude mixture was then directly purified by flash column chromatography (silica gel, 10-20% acetone:hexanes) to give **67-5** as a white solid (42 mg, 37% yield). mp = 60-63 ° C; ^{1}H NMR (400 MHz, $CDCl_3$) δ 8.76 (d, *J* = 2.6 Hz, 1H), 8.11 (d, *J* = 10.0 Hz, 1H), 8.05 (d, *J* = 3.5 Hz, 1H), 7.99 (d, *J* = 8.3 Hz, 2H), 7.57 (d, *J* = 8.5 Hz, 2H), 7.52 (d, *J* = 8.5 Hz, 1H), 7.42 (dd, *J* = 8.2, 4.4 Hz, 2H), 7.32 (d, *J* = 8.7 Hz, 1H), 6.65 – 6.58 (m, 1H), 6.57 – 6.46 (m, 2H), 6.28 (s, 1H), 4.34 (q, *J* = 7.2 Hz, 2H), 1.35 (t, *J* = 7.2 Hz, 3H); ^{13}C NMR (101 MHz, $CDCl_3$) δ 166.3, 157.2, 149.1, 148.3, 146.7, 146.3, 138.7, 138.2, 136.1, 129.9, 129.5, 127.8, 126.9, 126.6, 122.8, 122.0, 118.4, 113.4, 107.7, 60.9, 54.9, 14.3. IR neat film: 3375, 2978, 2929, 1710, 1598, 1502 cm^{-1}; HRMS (ESI) calculated for $[C_{24}H_{22}N_3O_3]^+$: 400.1656, found 400.1656.

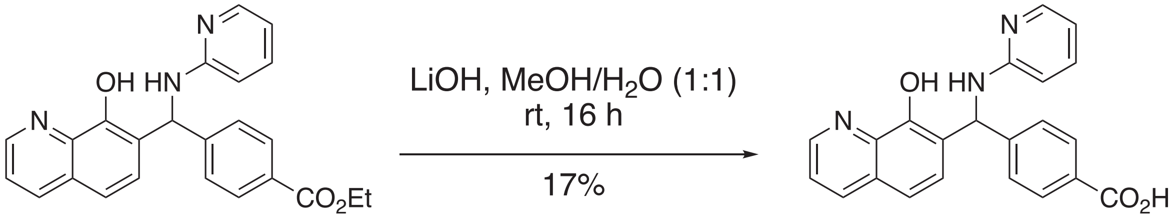

67-5 **67-1**

4-((8-hydroxyquinolin-7-yl)(pyridin-2-ylamino)methyl)benzoic acid (67-1)

To a 20 mL scintillation vial, lithium hydroxide monohydrate (55 mg, 1.3 mmol, 2.7 equiv) was dissolved in 4 mL H_2O and 2 mL MeOH. Ester **67-5** (194 mg, 0.49 mmol, 1.0 equiv) was dissolved in 2 mL MeOH and added to the vial. The vial was capped and stirred at rt for 16 h. Then 2M HCl was added dropwise to the solution until a precipitate persisted. Do not add too much HCl, as the product will dissolve. The precipitate was filtered, washed with 5 mL cold water, 5 mL cold Et_2O, and collected to give **67-1** as a white solid (30.2 mg, 17% yield). mp = 140-142 C; ^{1}H NMR (300 MHz, CD_3OD) δ 8.77 (d, *J* = 5.9 Hz, 1H), 8.17 (d, *J* = 6.7 Hz, 1H), 7.94 (d, *J* = 8.2 Hz, 3H), 7.56

– 7.40 (m, 5H), 7.32 (d, *J* = 8.6 Hz, 1H), 6.67 (t, *J* = 4.3 Hz, 2H), 6.58 (t, *J* = 6.2 Hz, 1H); ^{13}C NMR (75 MHz, CD_3OD) δ 170.6, 159.2, 151.4, 149.8, 148.7, 147.4, 140.1, 139.6, 137.4, 131.7, 131.0, 130.8, 129.6, 128.6, 128.4, 128.0, 125.5, 123.1, 119.3, 114.3, 110.4, 55.2; IR neat film: 3315, 2925, 1652, 1608, 1574 cm^{-1}; HRMS (ESI) calculated for $[C_{22}H_{18}N_3O_3]^+$: 372.1343, found 372.1343.

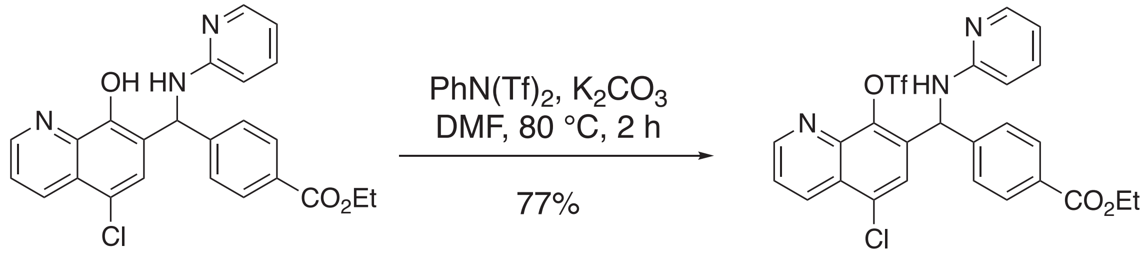

67-4 **S-5**

Ethyl 4-((5-chloro-8-(((trifluoromethyl)sulfonyl)oxy)quinolin-7-yl)(pyridin-2-ylamino)methyl)benzoate (S-5)

In a 50 mL pressure tube, **67-4** (440 mg, 1.0 mmol, 1.0 equiv.) was dissolved in dry DMF (5 mL) under argon atmosphere. *N*-Phenyl-bis(trifluoromethanesulfonimide) (537 mg, 1.51 mmol, 1.5 equiv.) and potassium carbonate (208 mg, 1.50 mmol, 1.5 equiv.) were added and the solution was 80 °C for 2 h. The solution was then cooled to rt, diluted with EtOAc, and washed three times with H_2O. The organic layer was dried over Na_2SO_4, and then concentrated. The crude mixture was purified by flash column chromatography (silica gel, 30% EtOAc:hexanes) to yield **S-5** as a yellow solid (451 mg, 77% yield). mp = 167-169 °C; ^1H NMR (400 MHz, $CDCl_3$) δ 9.04 (d, *J* = 4.2 Hz, 1H), 8.55 (d, *J* = 7.0 Hz, 1H), 8.11 – 7.99 (m, 3H), 7.78 (s, 1H), 7.62 (dd, *J* = 8.5, 4.3 Hz, 1H), 7.43 (d, *J* = 8.2 Hz, 3H), 6.66 (dd, *J* = 7.2, 5.0 Hz, 1H), 6.53 (d, *J* = 5.1 Hz, 1H), 6.41 (d, *J* = 8.3 Hz, 1H), 5.25 (d, *J* = 5.3 Hz, 1H), 4.36 (q, *J* = 7.1 Hz, 2H), 1.37 (t, *J* = 7.1 Hz, 3H); ^{13}C NMR (101 MHz, $CDCl_3$) δ 166.0, 156.8, 151.6, 148.3, 144.5, 137.9, 135.3, 133.0, 131.7, 130.4, 130.3, 127.4,

126.9, 125.2, 123.3, 114.7, 107.1, 61.1, 54.6, 14.3; IR neat film: 3379, 3066, 3001, 2980, 1725, 1603, 1601 cm^{-1}; HRMS (ESI) calculated for $[C_{25}H_{20}ClF_3N_3O_5S]^+$: 566.0759, found 566.0765.

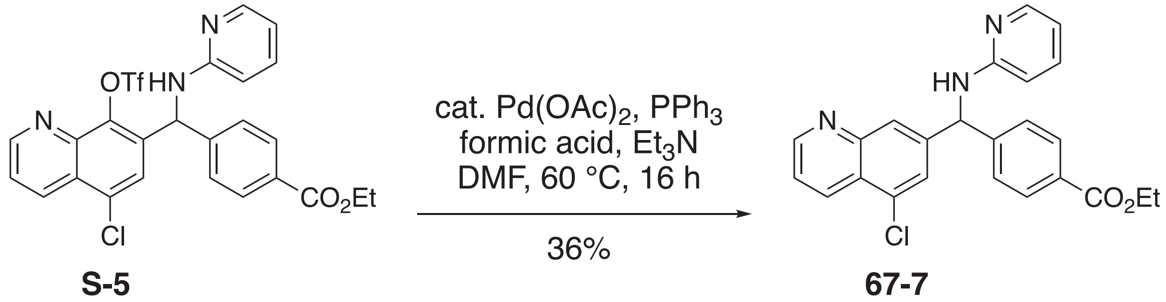

Ethyl 4-((5-chloroquinolin-7-yl)(pyridin-2-ylamino)methyl)benzoate (67-7)

In a 50 mL pressure tube, **S-5** (400 mg, 0.75 mmol, 1.0 equiv.), $Pd(OAc)_2$ (10 mg, 0.045 mmol, 0.06 equiv.), triphenylphosphine (23 mg, 0.088 mmol, 0.12 equiv.), and triethylamine (0.4 mL, 0.51 mmol, 3.8 equiv.) were combined in dry DMF (5 mL) under argon atmosphere.[9] Formic acid (70 µL, 1.86 mmol, 2.5 equiv.) was added, and the reaction was capped and stirred at 60 °C for 16 h. Upon cooling to rt, the reaction mixture was diluted with brine and extracted with EtOAc. The organic layer was then washed three times with brine. The organic layer was dried over Na_2SO_4 and then concentrated. The crude mixture was purified by flash column chromatography (silica gel, 30% EtOAc:hexanes gradient) to yield **67-7** as a yellow solid (106 mg, 36% yield). mp = 56-57 °C; ^{1}H NMR (300 MHz, $CDCl_3$) δ 8.91 (d, J = 4.3 Hz, 1H), 8.52 (d, J = 8.1 Hz, 1H), 8.07 (d, J = 4.4 Hz, 1H), 8.02 (d, J = 8.2 Hz, 3H), 7.60 (s, 1H), 7.51 – 7.43 (m, 3H), 7.39 (d, J = 6.8 Hz, 1H), 6.62 (d, J = 6.6 Hz, 1H), 6.39 (d, J = 8.3 Hz, 1H), 6.22 (d, J = 6.0 Hz, 1H), 5.25 (d, J = 6.1 Hz, 1H), 4.36 (q, J = 7.1 Hz, 2H), 1.37 (t, J = 7.1 Hz, 3H). ^{13}C NMR (75 MHz, $CDCl_3$) δ 166.1, 157.1, 151.4, 148.8, 148.2, 146.3, 143.7, 137.6, 132.7, 132.0, 130.2, 130.1, 127.6, 126.6, 126.3, 125.7, 122.0, 114.1, 107.7, 61.0, 59.7, 14.3; IR neat film: 3268, 2925, 2980, 1713, 1601, 1478 cm^{-1}; HRMS (ESI) calculated for $[C_{24}H_{20}ClN_3NaO_2]^+$: 440.1136, found 440.1143.

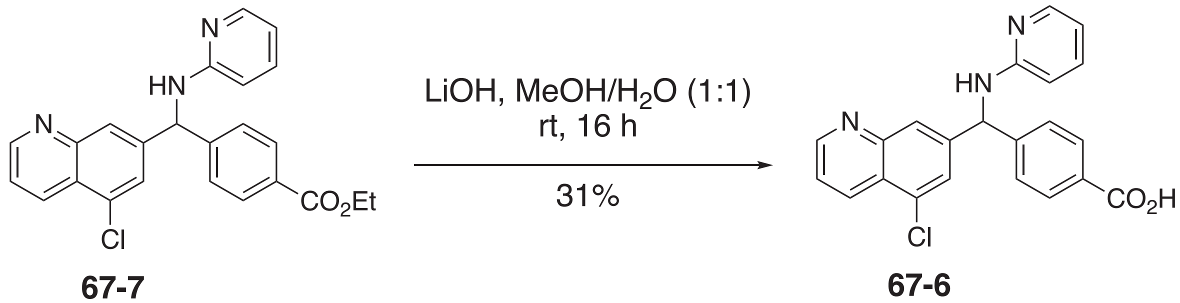

67-7 **67-6**

Ethyl 4-((5-chloroquinolin-7-yl)(pyridin-2-ylamino)methyl)benzoate (67-6)

To a 20 mL scintillation vial, lithium hydroxide monohydrate (30 mg, 0.71 mmol, 3.5 equiv) was dissolved in 2 mL H_2O and 1 mL MeOH. Ester **67-7** (86 mg, 0.21 mmol, 1.0 equiv) was dissolved in 1 mL MeOH and added to the vial. The vial was capped and stirred at rt for 16 h. Then 2M HCl was added dropwise to the solution until a precipitate persisted. The precipitate was filtered, washed with 5 mL cold water, 5 mL cold ether, and collected to give **67-6** as a white solid (24.5 mg, 31% yield). mp = 136-137 C; ^{1}H NMR (400 MHz, CD_3OD) δ 8.84 (d, J = 6.1 Hz, 1H), 8.59 (d, J = 8.5 Hz, 1H), 8.00 (d, J = 8.0 Hz, 2H), 7.97 – 7.87 (m, 2H), 7.70 (d, J = 1.7 Hz, 1H), 7.59 (dd, J = 8.5, 4.2 Hz, 1H), 7.47 (dd, J = 19.4, 7.4 Hz, 3H), 6.72 (d, J = 8.5 Hz, 1H), 6.59 (t, J = 6.2 Hz, 1H), 6.46 (s, 1H); ^{13}C NMR (101 MHz, CD_3OD) δ 169.8, 159.0, 152.5, 149.6, 148.1, 147.8, 146.5, 139.3, 134.6, 132.9, 131.7, 131.4, 129.2, 128.4, 127.0, 126.9, 123.6, 114.5, 111.1, 59.9; IR neat film: 3277, 2981, 1671, 1606, 1482 cm^{-1}; HRMS (ESI) calculated for [$C_{22}H_{17}ClN_3O_2$] 390.1004, found 390.1011.

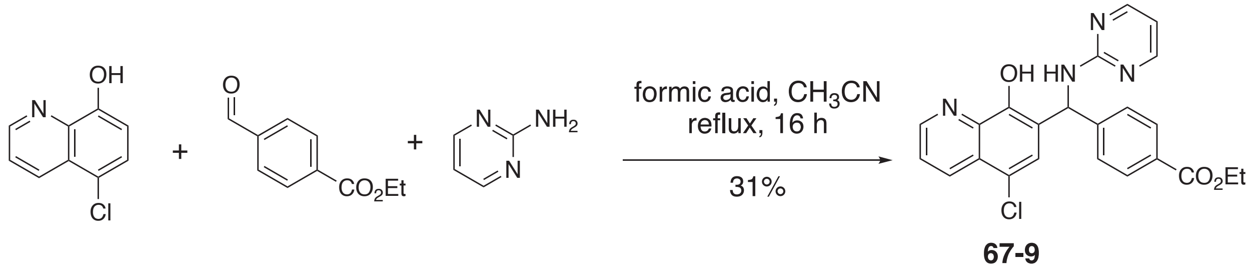

67-9

Ethyl 4-((5-chloro-8-hydroxyquinolin-7-yl)(pyrimidin-2-ylamino)methyl)benzoate (67-9)

To a 250 mL dry round bottomed flask equipped with a reflux condenser, 2-aminopyrimidine (4.3 g, 45.3 mmol, 1.2 equiv), ethyl 4-formylbenzoate (6.8 g, 38.2 mmol, 1.0 equiv.), and 5-chloro-8-hydroxyquinoline (8.2 g, 45.8 mmol, 1.2 equiv.) were dissolved in CH_3CN (100 mL). Following the addition of formic acid (1.4 mL, 37.1 mmol 1.0 equiv.), the solution was stirred at reflux for 16 h. The solution was allowed to cool to rt, concentrated, resuspended in acetone. The heterogenous mixture was filtered, and the precipitate was washed with cold acetone and hexanes to give **67-9** as a white solid (5.2 g, 31% yield). mp = 150-151 °C; ^{1}H NMR (300 MHz, $CDCl_3$) δ 8.80 (d, *J* = 4.2 Hz, 1H), 8.49 (d, *J* = 8.6 Hz, 1H), 8.30 (d, *J* = 4.8 Hz, 2H), 7.99 (d, *J* = 7.9 Hz, 2H), 7.55 (dd, *J* = 19.6, 10.2 Hz, 4H), 6.79 (d, *J* = 8.2 Hz, 1H), 6.58 (t, *J* = 4.9 Hz, 1H), 6.39 (d, *J* = 8.3 Hz, 1H), 4.34 (q, *J* = 7.2 Hz, 2H), 1.36 (t, *J* = 7.2 Hz, 3H); ^{13}C NMR (75 MHz, $CDCl_3$) δ 166.3, 161.5, 158.2, 148.7, 148.5, 146.6, 138.7, 133.3, 129.8, 129.4, 127.0, 126.8, 125.6, 123.6, 122.5, 120.7, 111.5, 60.9, 54.8, 14.3; IR neat film: 3293, 2978, 1716, 1583, 1496 cm^{-1}; HRMS (ESI) calculated for [$C_{23}H_{20}ClN_4O_3$] 390.1004, found 390.1011.

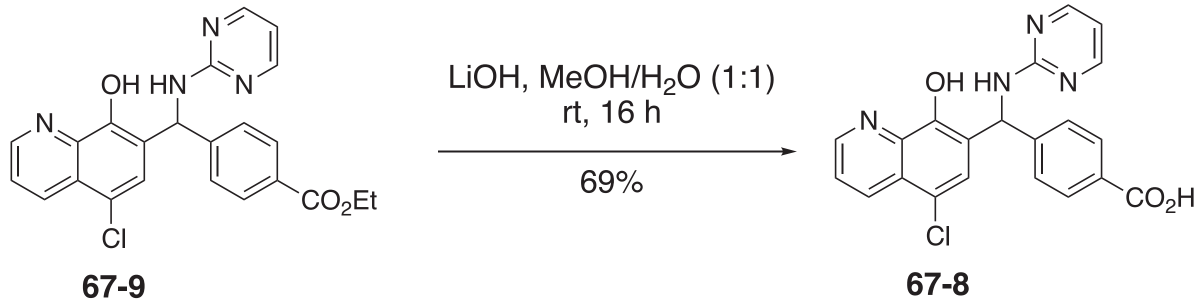

4-((5-Chloro-8-hydroxyquinolin-7-yl)(pyrimidin-2-ylamino)methyl)benzoic acid (67-8)

To a 20 mL scintillation vial, lithium hydroxide monohydrate (54 mg, 1.29 mmol, 5.6 equiv.) was dissolved in 2 mL H_2O and 1 mL MeOH. Ester **67-9** (100 mg, 0.21 mmol, 1.0 equiv.) was dissolved in 1 mL MeOH and added to the vial. The vial was capped and stirred at rt for 16 h. Then 2M HCl was added dropwise to the solution until a precipitate persisted. The precipitate was filtered, washed with 5 mL cold water, 5 mL cold ether, and collected to give **67-8** as a brown solid (65 mg, 31% yield). mp = 174-176 °C; ^{1}H NMR (300 MHz, DMSO-d_6) δ 10.47 (s, 1H), 8.95 (s, 1H), 8.46 (d, J = 8.5 Hz, 1H), 8.30 (t, J = 7.9 Hz, 3H), 8.03 – 7.79 (m, 3H), 7.79 – 7.62 (m, 1H), 7.46 (d, J = 7.9 Hz, 2H), 7.05 (d, J = 9.4 Hz, 1H), 6.63 (d, J = 4.9 Hz, 1H); ^{13}C NMR (75 MHz, DMSO-d_6) δ 167.4, 161.6, 158.2, 149.6, 149.1, 146.9, 138.7, 132.5, 129.4, 127.0, 126.6, 125.5, 124.9, 123.0, 111.0, 51.4; IR neat film: 3291, 1673, 1576, 1501 cm^{-1}; HRMS (ESI) calculated for [$C_{21}H_{16}ClN_4O_3$] 407.0905, found 407.0913.

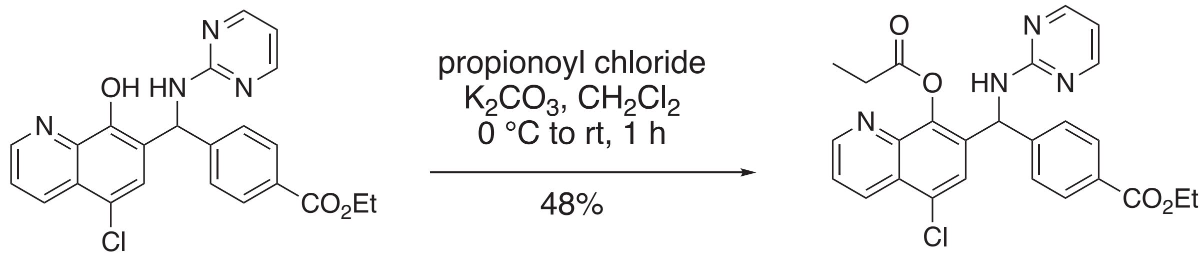

67-9 **MMRi71**

Ethyl 4-((8-(propionyloxy)quinolin-7-yl)(pyrimidin-2-ylamino)methyl)benzoate (MMRi71)

In a 5 mL dry round bottomed flask, **67-9** (5.0 g, 11.5 mmol, 1.0 equiv.) was dissolved in of dry CH_2Cl_2 (100 mL) under argon atmosphere. Potassium carbonate (3.18 g, 23.0 mmol, 2.0 equiv.) was added and the solution was cooled to 0 °C. Propionoyl chloride (1.0 mL, 11.5 mmol, 1.0 equiv.) was then added to the solution. The mixture was allowed to warm to rt and stirred for 1 h. The reaction mixture was then filtered through celite and washed with CH_2Cl_2. The supernatant was then treated with 1 g DMT-functionalized silica gel and stirred for 15 minutes. The mixture was filtered and concentrated. The resulting crude solid was resuspended in ether and washed with distilled water. The organic layer was dried over Na_2SO_4 and then concentrated. The crude mixture was purified by flash column chromatography (silica gel, 50% Et_2O : hexanes) to yield **MMRi71** as a greenish white solid (2.53 g, 48% yield). mp = 178-179 °C; ^{1}H NMR (400 MHz, $CDCl_3$) δ 8.91 (d, J = 2.8 Hz, 1H), 8.50 (d, J = 6.8 Hz, 1H), 8.23 (d, J = 4.8 Hz, 2H), 8.00 (d, J = 8.4 Hz, 2H), 7.54 (s, 1H), 7.50 (dd, J = 8.5, 4.2 Hz, 1H), 7.43 (d, J = 8.1 Hz, 2H), 6.83 (d, J = 7.8 Hz, 1H), 6.57 (t, J = 4.8 Hz, 1H), 6.10 (d, J = 7.8 Hz, 1H), 4.36 (q, J = 7.1 Hz, 2H), 2.68 (q, J = 7.6 Hz, 2H), 1.37 (t, J = 7.1 Hz, 3H), 1.20 (t, J = 7.5 Hz, 3H); ^{13}C NMR (101 MHz, $CDCl_3$) δ 172.2, 166.2, 161.3, 158.1, 151.2, 145.5, 144.6, 142.0, 134.3, 133.0, 130.0, 129.8, 129.0, 127.1, 126.7, 125.7, 122.4, 111.8, 61.0, 53.8, 27.3, 14.3, 9.0. IR neat film: 3270, 2982, 1773, 1716, 1578, 1491 cm^{-1}; HRMS (ESI) calculated for [$C_{26}H_{23}ClN_4NaO_4$] 513.1300, found 513.1307.

Data File C:\Chem32\1\Data\Luke\MMRi71_HPLCTrace 2022-10-21 14-43-04.D
Sample Name: MMRi71_HPLCTrace

```
=====================================================================
Acq. Operator   : SYSTEM
Sample Operator : SYSTEM
Acq. Instrument : Terry                          Location :   1
Injection Date  : 10/21/2022 2:43:04 PM
                                              Inj Volume : Manually
Acq. Method     : C:\CHEM32\1\METHODS\LD_P262.M
Last changed    : 10/21/2022 2:26:14 PM by SYSTEM
                  (modified after loading)
Analysis Method : C:\CHEM32\1\METHODS\AK_1_63_ester.M
Last changed    : 10/24/2022 12:20:41 PM by SYSTEM
                  (modified after loading)
Additional Info : Peak(s) manually integrated
```

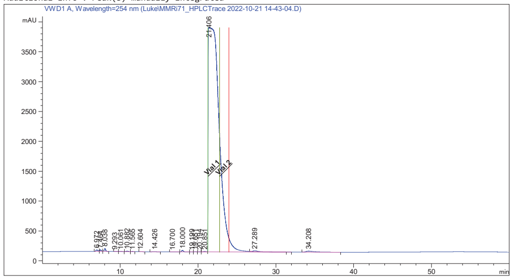


```
=================================================================================
                         Fraction Information
=================================================================================
Fraction List
=================================================================================
Frac  Well  Location   Volume    BeginTime  EndTime    Reason        Mass
  #     #              [µl]      [min]      [min]
-----|-----|----------|----------|----------|----------|------------|------------
  1     1    Vial 1     15000.00     21.264     22.764  VWD1
  1     1    Vial 2     12226.67     22.770     23.981  Overfill
=================================================================================
```

Terry 10/24/2022 12:20:46 PM SYSTEM   Page   1 of 2

MMRi71 HPLC trace, >95% pure

Data File C:\Chem32\1\Data\Luke\MMRi71_HPLCTrace 2022-10-21 14-43-04.D
Sample Name: MMRi71_HPLCTrace

```
=====================================================================
                         Area Percent Report
=====================================================================

Sorted By             :      Signal
Multiplier            :      1.0000
Dilution              :      1.0000
Use Multiplier & Dilution Factor with ISTDs


Signal 1: VWD1 A, Wavelength=254 nm
```

Peak #	RetTime [min]	Type	Width [min]	Area [mAU*s]	Height [mAU]	Area %
1	6.972	BV	0.2672	480.75177	26.06481	0.1291
2	7.464	VB	0.1571	294.47696	27.45437	0.0791
3	8.038	BB	0.1747	560.89160	48.56438	0.1506
4	9.293	VB	0.2081	36.80785	2.49552	9.881e-3
5	10.061	BB	0.1931	42.67840	3.43127	0.0115
6	10.882	BV	0.2126	148.04672	10.49339	0.0397
7	11.565	VB	0.2439	40.99703	2.39274	0.0110
8	12.604	BB	0.2147	36.51181	2.55504	9.802e-3
9	14.426	BB	0.3958	127.56663	4.27884	0.0342
10	16.700	BB	0.3476	47.97471	1.97325	0.0129
11	18.000	BB	0.2769	485.77301	26.61434	0.1304
12	19.199	BV	0.2564	41.51525	2.51803	0.0111
13	19.651	VB	0.2566	86.28642	5.22560	0.0232
14	20.194	BB	0.2398	28.79559	1.91104	7.730e-3
15	20.851	BV E	0.3977	173.21483	6.12889	0.0465
16	21.406	VV R	1.1841	3.67756e5	3750.14941	98.7276
17	27.289	VB E	0.7139	794.78540	15.61395	0.2134
18	34.208	BB	1.0974	1312.61426	17.21818	0.3524
Totals :				3.72496e5	3955.08305	

```
=====================================================================
                         *** End of Report ***
```

References:

1. McLean, L. R.; Zhang, Y.; Li, H.; Li, Z.; Lukasczyk, U.; Choi, Y. M.; Han, Z.; Prisco, J.; Fordham, J.; Tsay, J. T.; Reiling, S.; Vaz, R. J.; Li, Y., Discovery of covalent inhibitors for MIF tautomerase via cocrystal structures with phantom hits from virtual screening. *Bioorg. Med. Chem. Lett.* **2009,** *19*, 6717-6720.
2. Shergalis, A.; Xue, D.; Gharbia, F. Z.; Driks, H.; Shrestha, B.; Tanweer, A.; Cromer, K.; Ljungman, M.; Neamati, N., Characterization of Aminobenzylphenols as Protein Disulfide Isomerase Inhibitors in Glioblastoma Cell Lines. *J. Med. Chem.* **2020,** *63*, 10263-10286.
3. Weinert, E. E.; Dondi, R.; Colloredo-Melz, S.; Frankenfield, K. N.; Mitchell, C. H.; Freccero, M.; Roika, S. E., Substituents on Quinone Methides Strongly Modulate Formation and Stability of Their Nucleophilic Adducts. *J. Am. Chem. Soc.* **2006,** *128*, 11940-11947.
4. Kanizsai, I.; Madacsi, R.; Hackler Jr., L.; Gyuris, M.; Szebeni, G. J.; Huzian, O.; Puskas, L. G., Synthesis and Cytoprotective Characterization of 8-Hydroxyquinoline Betti Products. *Molecules* **2018,** *23*, 1934.
5. Pobbati, A. V.; Mejuch, T.; Chakraborty, S.; Karatas, H.; Bharath, S. R.; Gueret, S. M.; Goy, P. A.; Hahne, G.; Pahl, A.; Sievers, S.; Guccione, E.; Song, H.; Waldmann, H.; Hong, W., Identification of Quinolinols as Activators of TEAD-Dependent Transcription. *ACS Chemical Biology* **2019,** *14*, 2909-2921.
6. Olyaei, A.; Sadeghpour, M., Recent advances in the synthesis and applications of Betti base (aminoalkylnaphthol) and bis-Betti base derivatives. *RSC Adv.* **2019,** *9*, 18467-18497.
7. Lim, C. K.; Popov, A. A.; Tselikov, G.; Heo, J.; Pliss, A.; Kim, S.; Kebashin, A. V.; Prasad, P. N., Organic Solvent and Surfactant Free Fluorescent Organic Nanoparticles by Laser Ablation of Aggregation-Induced Enhanced Emission Dyes. *Adv. Opt. Mat.* **2018,** *6*, 1800164.
8. Pauli, G. F.; Chen, S. N.; Simmler, C.; Lankin, D. C.; Godecke, T.; Jaki, B. U.; Friesen, J. B.; McAlpine, J. B.; Napolitano, J. G., Importance of Purity Evaluation and the Potential of Quantitative 1H NMR as a Purity Assay. *J. Med. Chem.* **2014,** *57*, 9220-9231.
9. Chen, Q. Y.; He, Y. B.; Yang, Z. Y., A new method for reduction of phenyl fluoroalkanesulfonates to arenes catalyzed by palladium. *J. Chem. Soc. Chem. Commun.* **1986,** *18*, 1452-1453.
10. Andreu, R.; Ronda, J. C., Synthesis of 3,4-dihydro-2H-1,3-benzoxazines by condensation fo 2-hydroxyaldehydes and primary amines: application to the synthesis of hydroxy-substituted and deuterium-labeled compounds. *Synth. Comm.* **2008,** *38*, 2316-2329.

Supporting Information:
Spectral Data of New Compounds

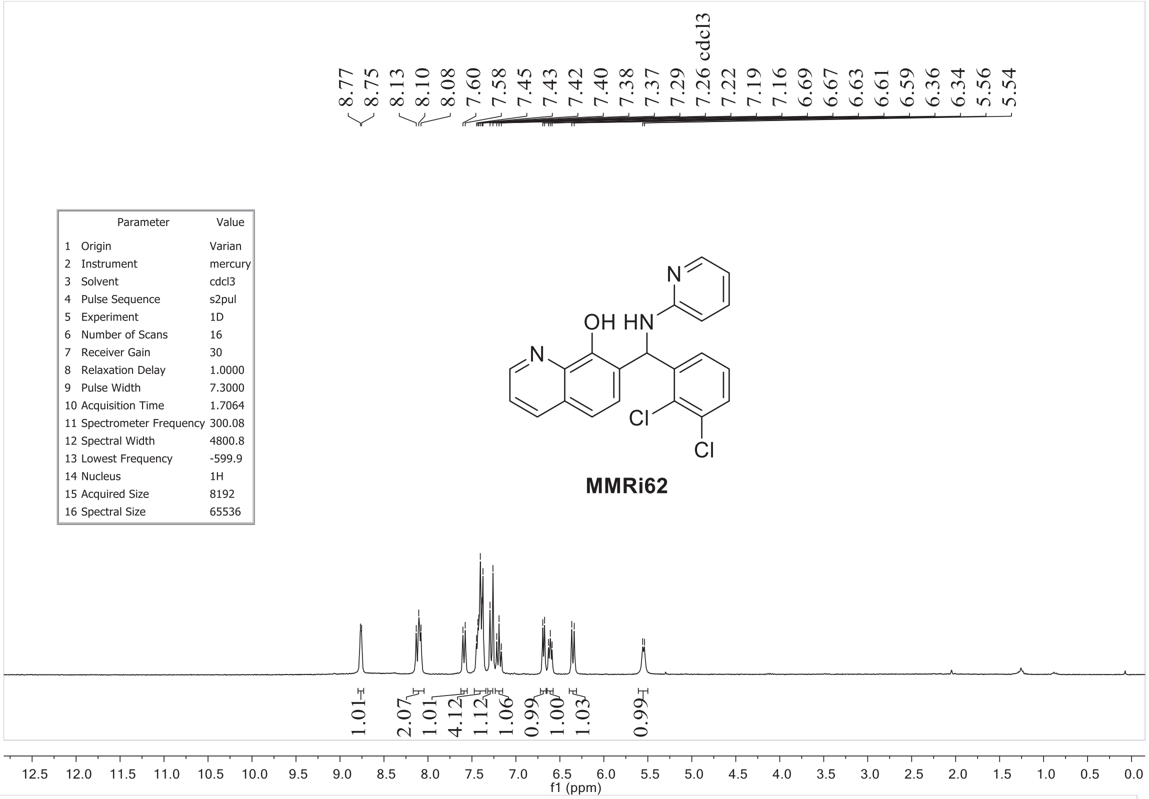

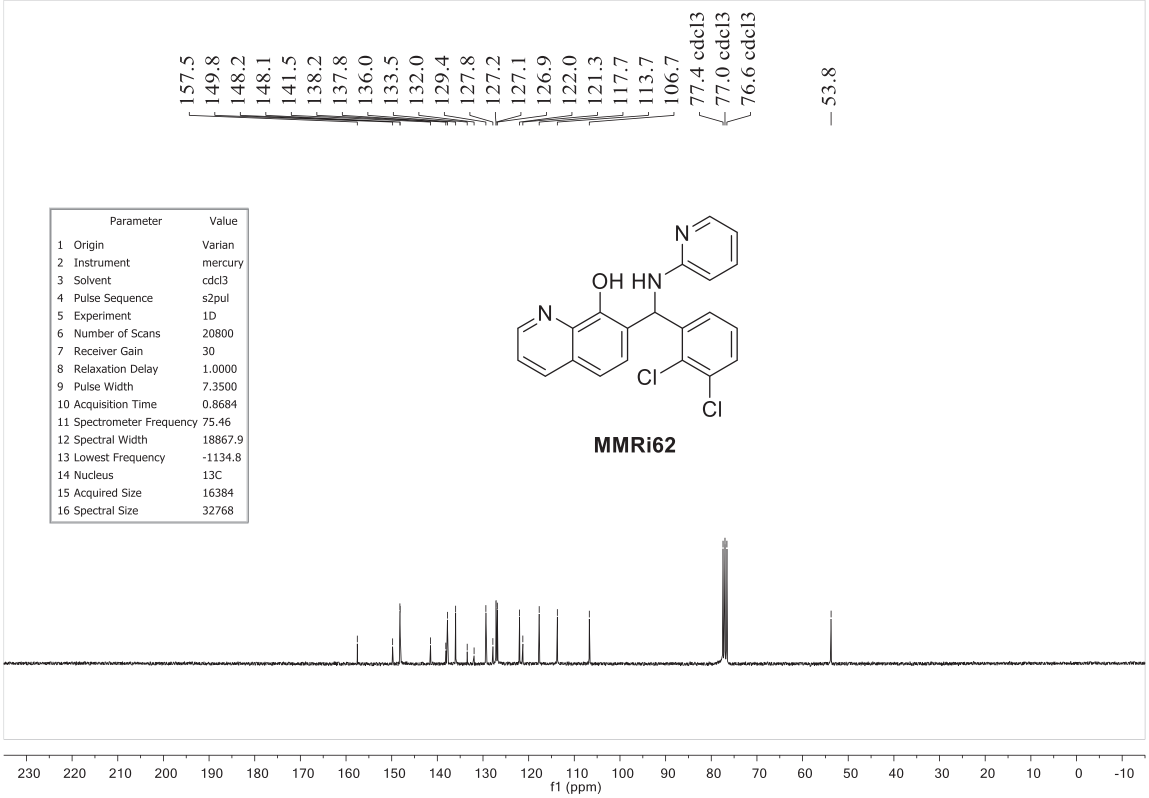

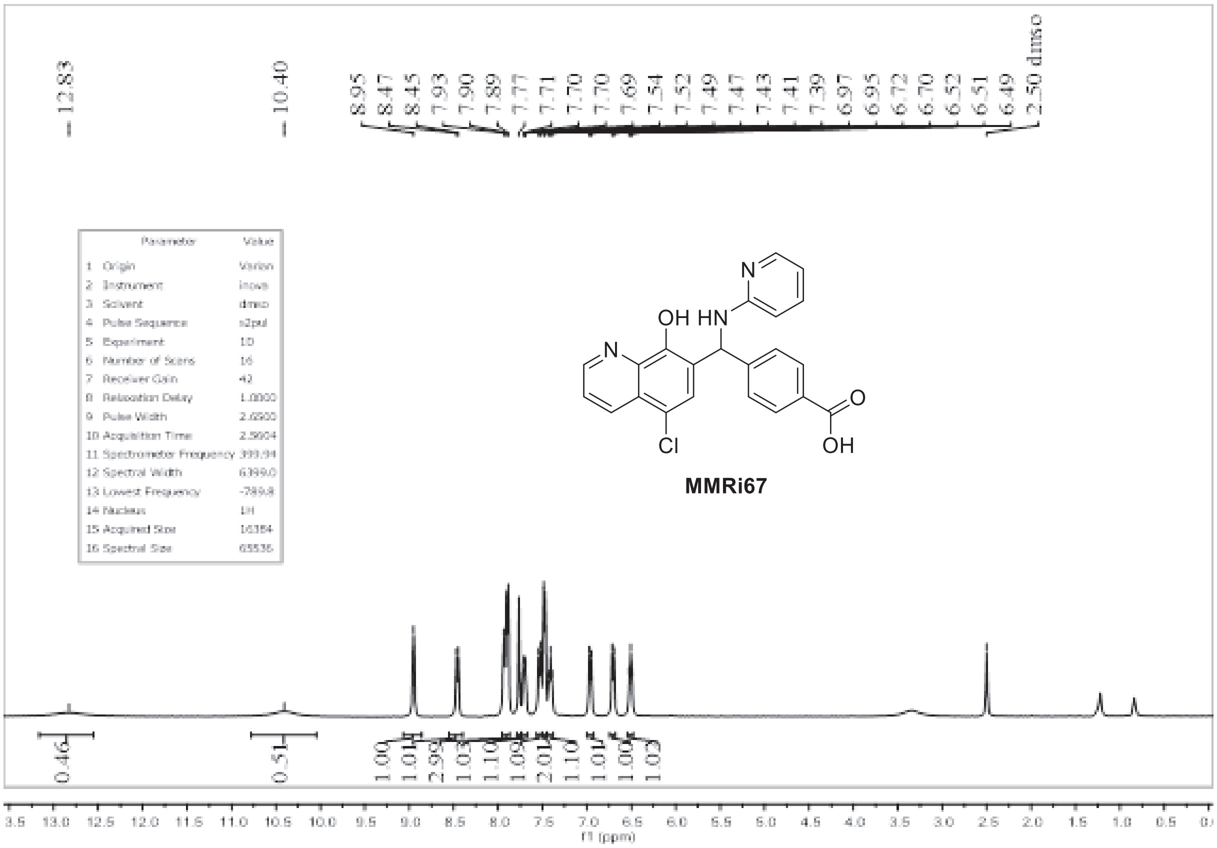
12.83
10.40
8.95 8.47 8.45 7.93 7.90 7.89 7.77 7.71 7.70 7.70 7.69 7.54 7.52 7.49 7.47 7.43 7.41 7.39 6.97 6.95 6.72 6.70 6.52 6.51 6.49
2.50 dmso
Parameter Value
1 Origin Varian
2 Instrument inova
3 Solvent dmso
4 Pulse Sequence s2pul
5 Experiment 1D
6 Number of Scans 16
7 Receiver Gain 42
8 Relaxation Delay 1.0000
9 Pulse Width 2.6900
10 Acquisition Time 2.5604
11 Spectrometer Frequency 399.94
12 Spectral Width 6399.0
13 Lowest Frequency -789.8
14 Nucleus 1H
15 Acquired Size 16384
16 Spectral Size 65536
OH HN
N
Cl
O
OH
MMRi67
0.46
0.51
1.00 1.01 2.98 1.03 1.10 1.09 2.01 1.10 1.01 1.00 1.03
f1 (ppm)

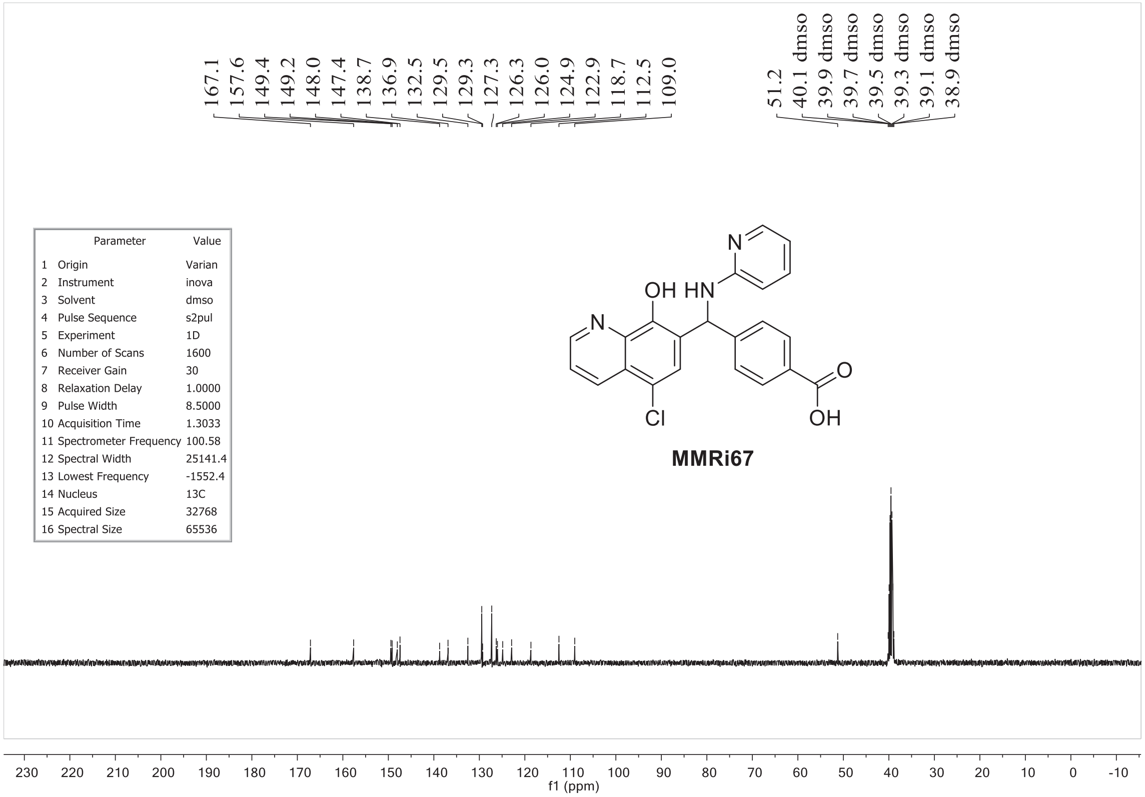
167.1 157.6 149.4 149.2 148.0 147.4 138.7 136.9 132.5 129.5 129.3 127.3 126.3 126.0 124.9 122.9 118.7 112.5 109.0
51.2 40.1 dmso 39.9 dmso 39.7 dmso 39.5 dmso 39.3 dmso 39.1 dmso 38.9 dmso
Parameter Value
1 Origin Varian
2 Instrument inova
3 Solvent dmso
4 Pulse Sequence s2pul
5 Experiment 1D
6 Number of Scans 1600
7 Receiver Gain 30
8 Relaxation Delay 1.0000
9 Pulse Width 8.5000
10 Acquisition Time 1.3033
11 Spectrometer Frequency 100.58
12 Spectral Width 25141.4
13 Lowest Frequency -1552.4
14 Nucleus 13C
15 Acquired Size 32768
16 Spectral Size 65536
OH HN
N
Cl
O
OH
MMRi67
f1 (ppm)

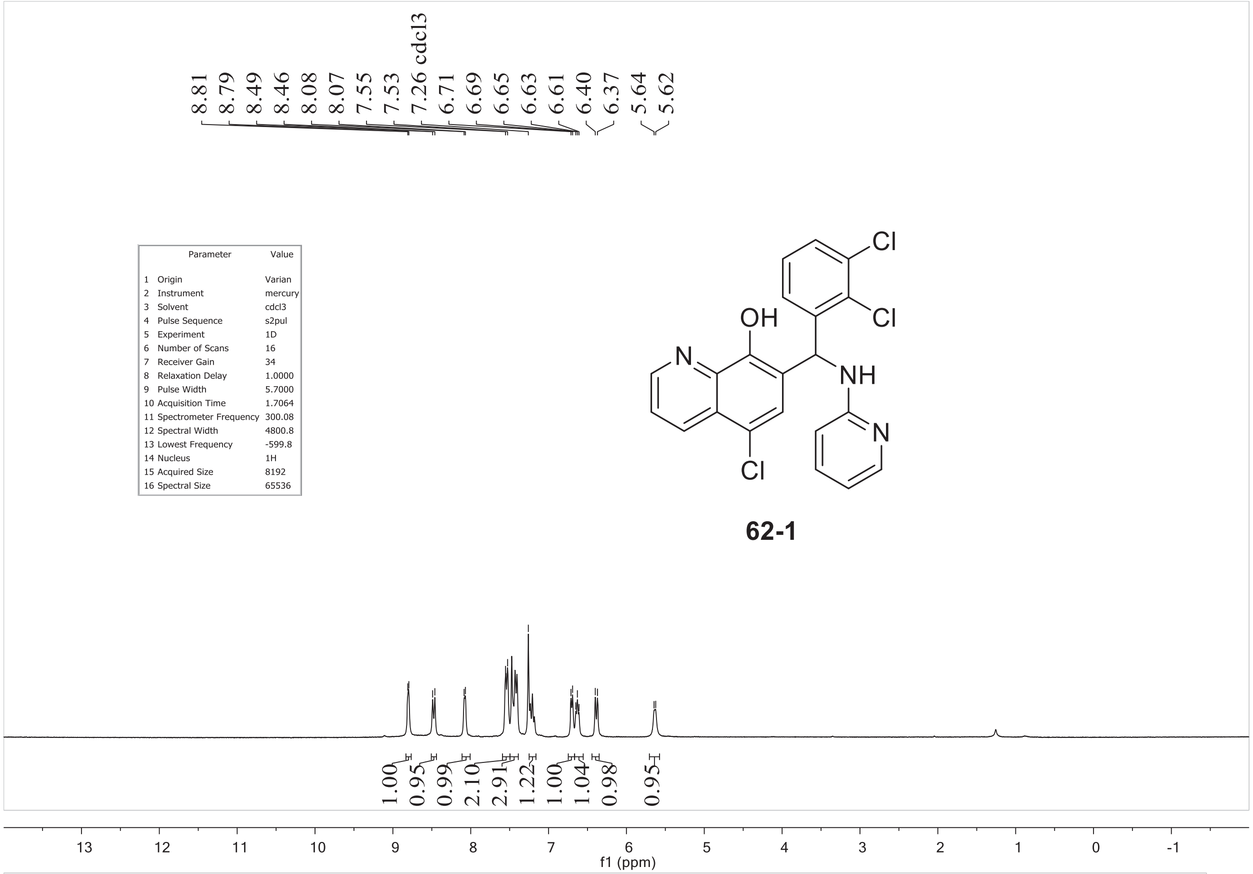
8.81
8.79
8.49
8.46
8.08
8.07
7.55
7.53
7.26 cdcl3
6.71
6.69
6.65
6.63
6.61
6.40
6.37
5.64
5.62
Parameter Value
1 Origin Varian
2 Instrument mercury
3 Solvent cdcl3
4 Pulse Sequence s2pul
5 Experiment 1D
6 Number of Scans 16
7 Receiver Gain 34
8 Relaxation Delay 1.0000
9 Pulse Width 5.7000
10 Acquisition Time 1.7064
11 Spectrometer Frequency 300.08
12 Spectral Width 4800.8
13 Lowest Frequency -599.8
14 Nucleus 1H
15 Acquired Size 8192
16 Spectral Size 65536
OH
Cl
Cl
N
NH
N
Cl
62-1
1.00
0.95
0.99
2.10
2.91
1.22
1.00
1.04
0.98
0.95
13 12 11 10 9 8 7 6 5 4 3 2 1 0 -1
f1 (ppm)

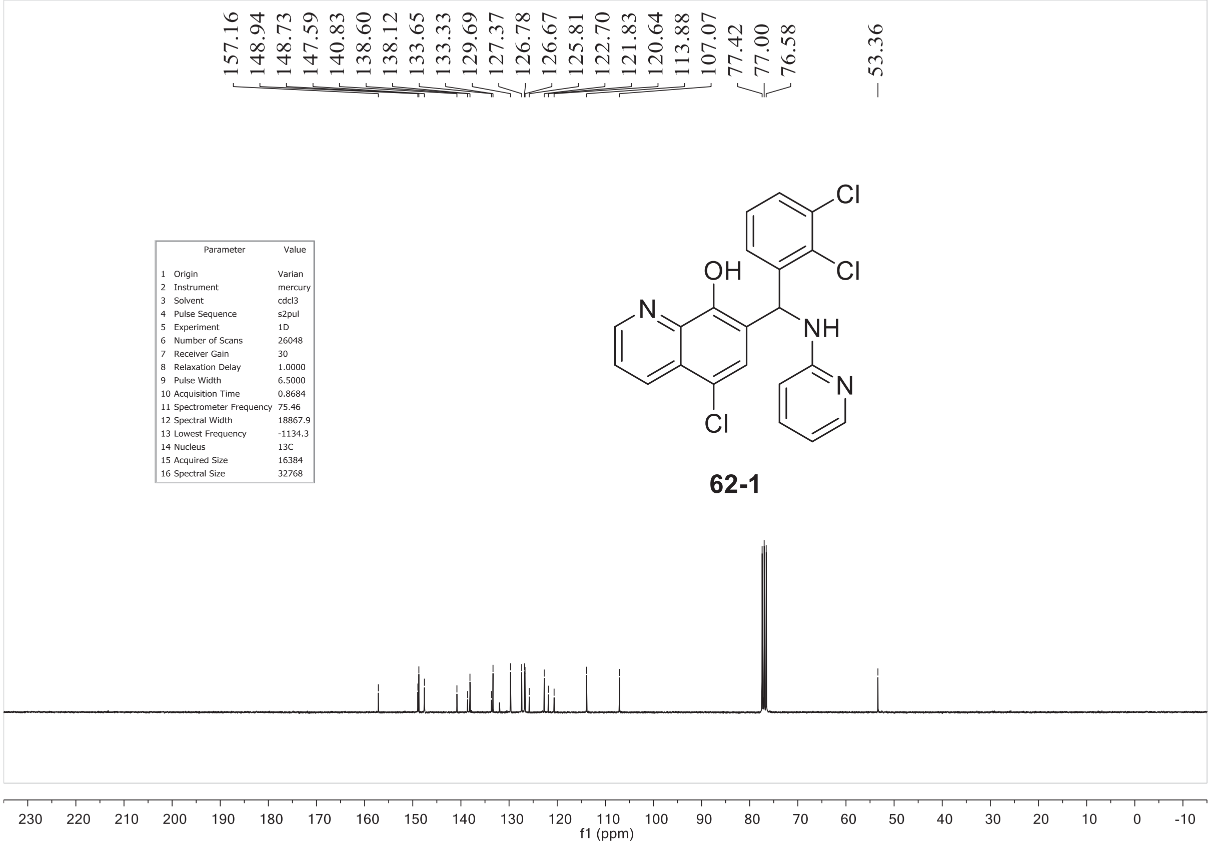
157.16
148.94
148.73
147.59
140.83
138.60
138.12
133.65
133.33
129.69
127.37
126.78
126.67
125.81
122.70
121.83
120.64
113.88
107.07
77.42
77.00
76.58
53.36
Parameter Value
1 Origin Varian
2 Instrument mercury
3 Solvent cdcl3
4 Pulse Sequence s2pul
5 Experiment 1D
6 Number of Scans 26048
7 Receiver Gain 30
8 Relaxation Delay 1.0000
9 Pulse Width 6.5000
10 Acquisition Time 0.8684
11 Spectrometer Frequency 75.46
12 Spectral Width 18867.9
13 Lowest Frequency -1134.3
14 Nucleus 13C
15 Acquired Size 16384
16 Spectral Size 32768
OH
Cl
Cl
N
NH
N
Cl
62-1
230 220 210 200 190 180 170 160 150 140 130 120 110 100 90 80 70 60 50 40 30 20 10 0 -10
f1 (ppm)

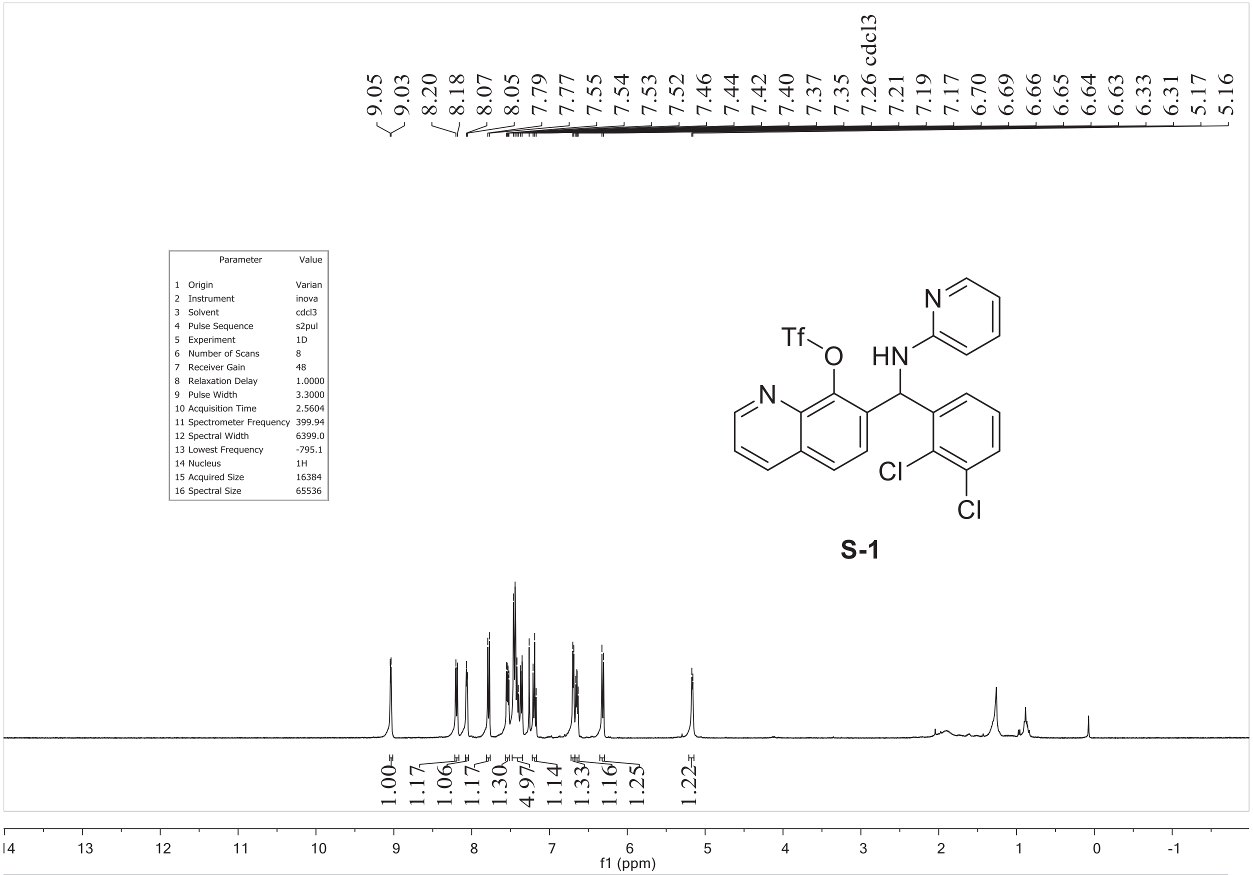
9.05
9.03
8.20
8.18
8.07
8.05
7.79
7.77
7.55
7.54
7.53
7.52
7.46
7.44
7.42
7.40
7.37
7.35
7.26 cdcl3
7.21
7.19
7.17
6.70
6.69
6.66
6.65
6.64
6.63
6.33
6.31
5.17
5.16
Parameter Value
1 Origin Varian
2 Instrument inova
3 Solvent cdcl3
4 Pulse Sequence s2pul
5 Experiment 1D
6 Number of Scans 8
7 Receiver Gain 48
8 Relaxation Delay 1.0000
9 Pulse Width 3.3000
10 Acquisition Time 2.5604
11 Spectrometer Frequency 399.94
12 Spectral Width 6399.0
13 Lowest Frequency -795.1
14 Nucleus 1H
15 Acquired Size 16384
16 Spectral Size 65536
Tf
O
HN
N
N
Cl
Cl
S-1
1.00
1.17
1.06
1.17
1.30
4.97
1.14
1.33
1.16
1.25
1.22
14 13 12 11 10 9 8 7 6 5 4 3 2 1 0 -1
f1 (ppm)

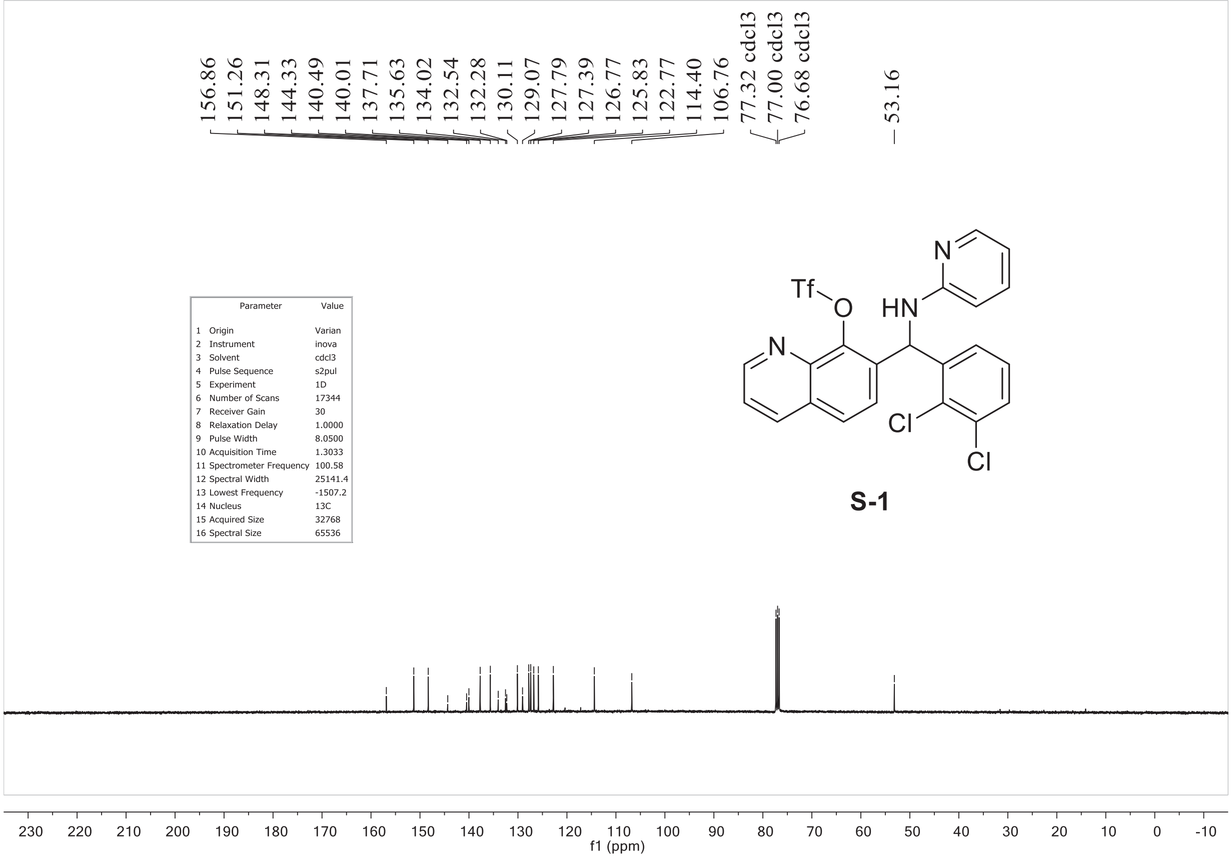
156.86
151.26
148.31
144.33
140.49
140.01
137.71
135.63
134.02
132.54
132.28
130.11
129.07
127.79
127.39
126.77
125.83
122.77
114.40
106.76
77.32 cdcl3
77.00 cdcl3
76.68 cdcl3
53.16
Parameter Value
1 Origin Varian
2 Instrument inova
3 Solvent cdcl3
4 Pulse Sequence s2pul
5 Experiment 1D
6 Number of Scans 17344
7 Receiver Gain 30
8 Relaxation Delay 1.0000
9 Pulse Width 8.0500
10 Acquisition Time 1.3033
11 Spectrometer Frequency 100.58
12 Spectral Width 25141.4
13 Lowest Frequency -1507.2
14 Nucleus 13C
15 Acquired Size 32768
16 Spectral Size 65536
Tf
O
HN
N
N
Cl
Cl
S-1
230 220 210 200 190 180 170 160 150 140 130 120 110 100 90 80 70 60 50 40 30 20 10 0 -10
f1 (ppm)

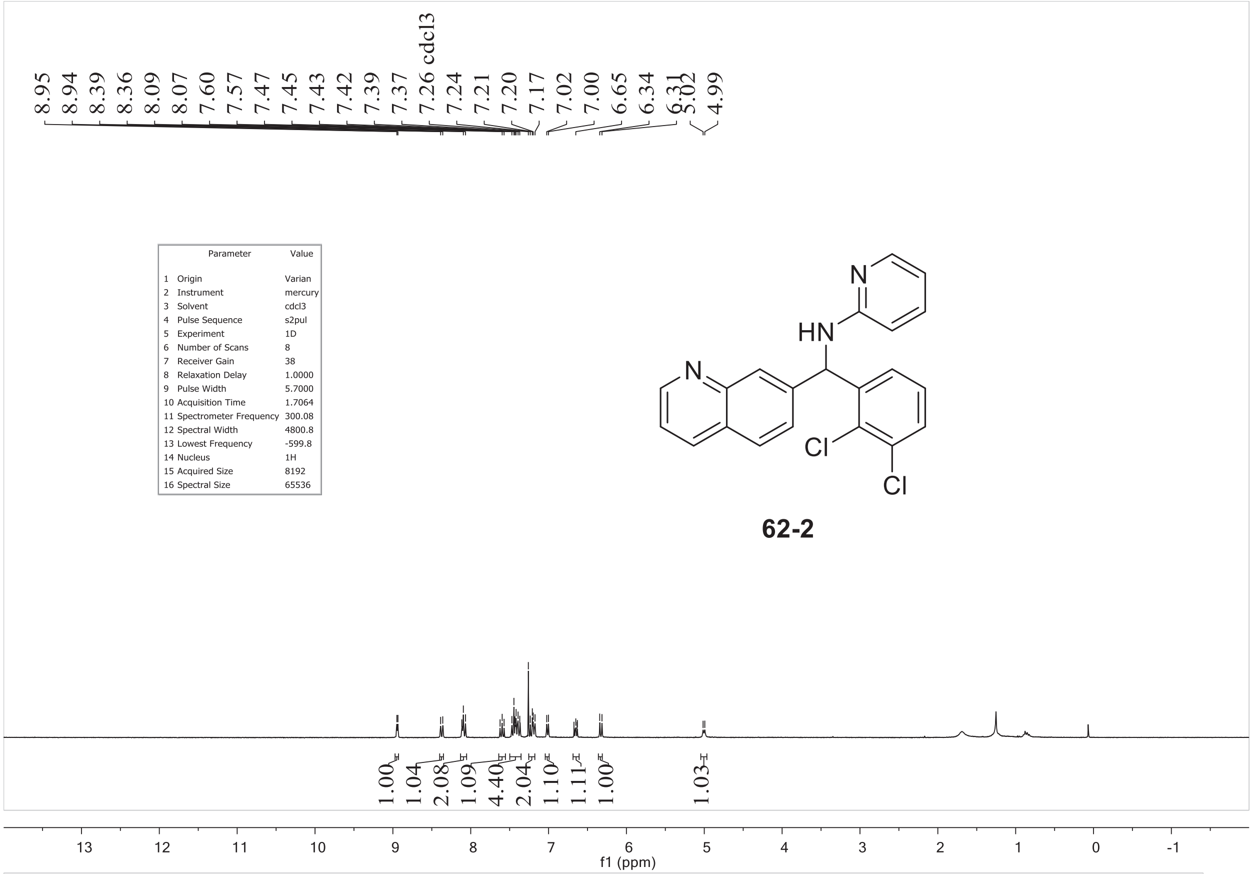
8.95 8.94 8.39 8.36 8.09 8.07 7.60 7.57 7.47 7.45 7.43 7.42 7.39 7.37 7.26 cdcl3 7.24 7.21 7.20 7.17 7.02 7.00 6.65 6.34 6.31 5.02 4.99
Parameter Value
1 Origin Varian
2 Instrument mercury
3 Solvent cdcl3
4 Pulse Sequence s2pul
5 Experiment 1D
6 Number of Scans 8
7 Receiver Gain 38
8 Relaxation Delay 1.0000
9 Pulse Width 5.7000
10 Acquisition Time 1.7064
11 Spectrometer Frequency 300.08
12 Spectral Width 4800.8
13 Lowest Frequency -599.8
14 Nucleus 1H
15 Acquired Size 8192
16 Spectral Size 65536
N
HN
N
Cl
Cl
62-2
1.00 1.04 2.08 1.09 4.40 2.04 1.10 1.11 1.00 1.03
13 12 11 10 9 8 7 6 5 4 3 2 1 0 -1
f1 (ppm)

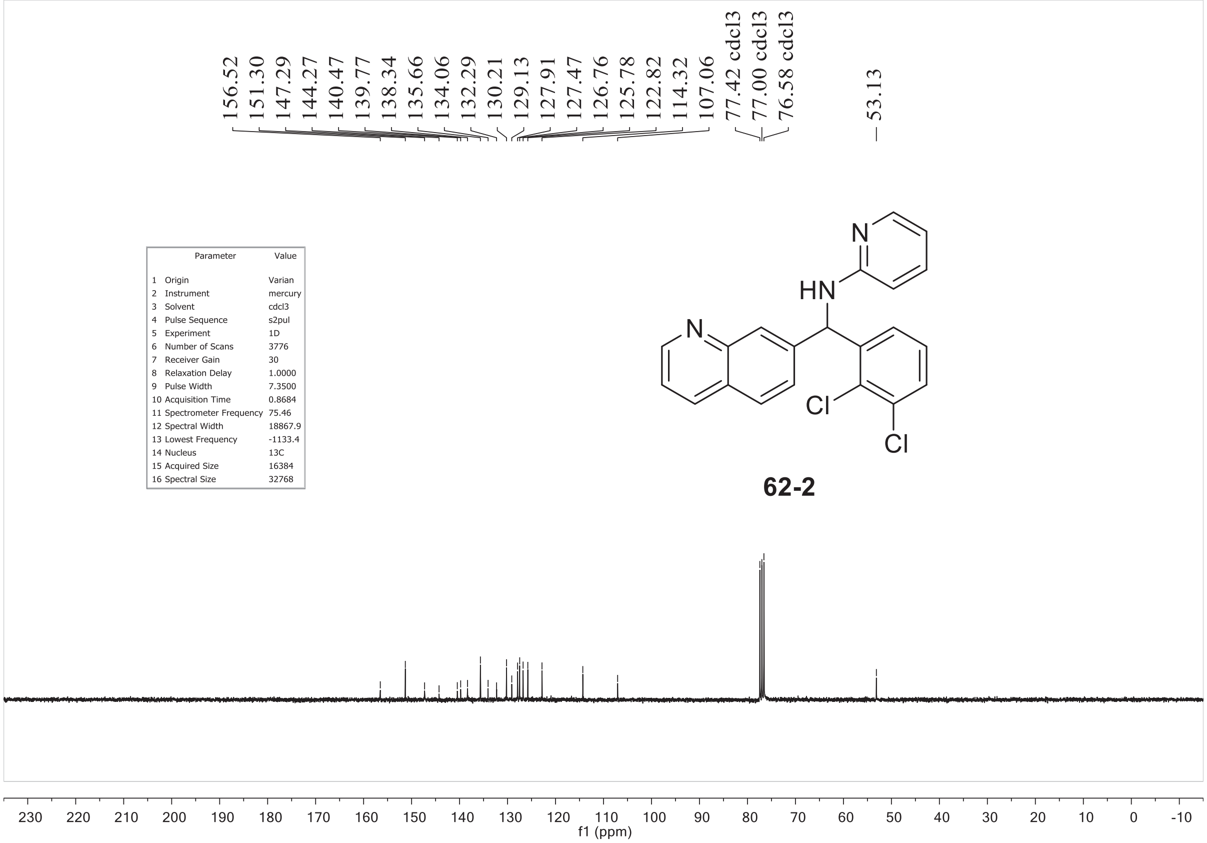
156.52 151.30 147.29 144.27 140.47 139.77 138.34 135.66 134.06 132.29 130.21 129.13 127.91 127.47 126.76 125.78 122.82 114.32 107.06 77.42 cdcl3 77.00 cdcl3 76.58 cdcl3 53.13
Parameter Value
1 Origin Varian
2 Instrument mercury
3 Solvent cdcl3
4 Pulse Sequence s2pul
5 Experiment 1D
6 Number of Scans 3776
7 Receiver Gain 30
8 Relaxation Delay 1.0000
9 Pulse Width 7.3500
10 Acquisition Time 0.8684
11 Spectrometer Frequency 75.46
12 Spectral Width 18867.9
13 Lowest Frequency -1133.4
14 Nucleus 13C
15 Acquired Size 16384
16 Spectral Size 32768
N
HN
N
Cl
Cl
62-2
230 220 210 200 190 180 170 160 150 140 130 120 110 100 90 80 70 60 50 40 30 20 10 0 -10
f1 (ppm)

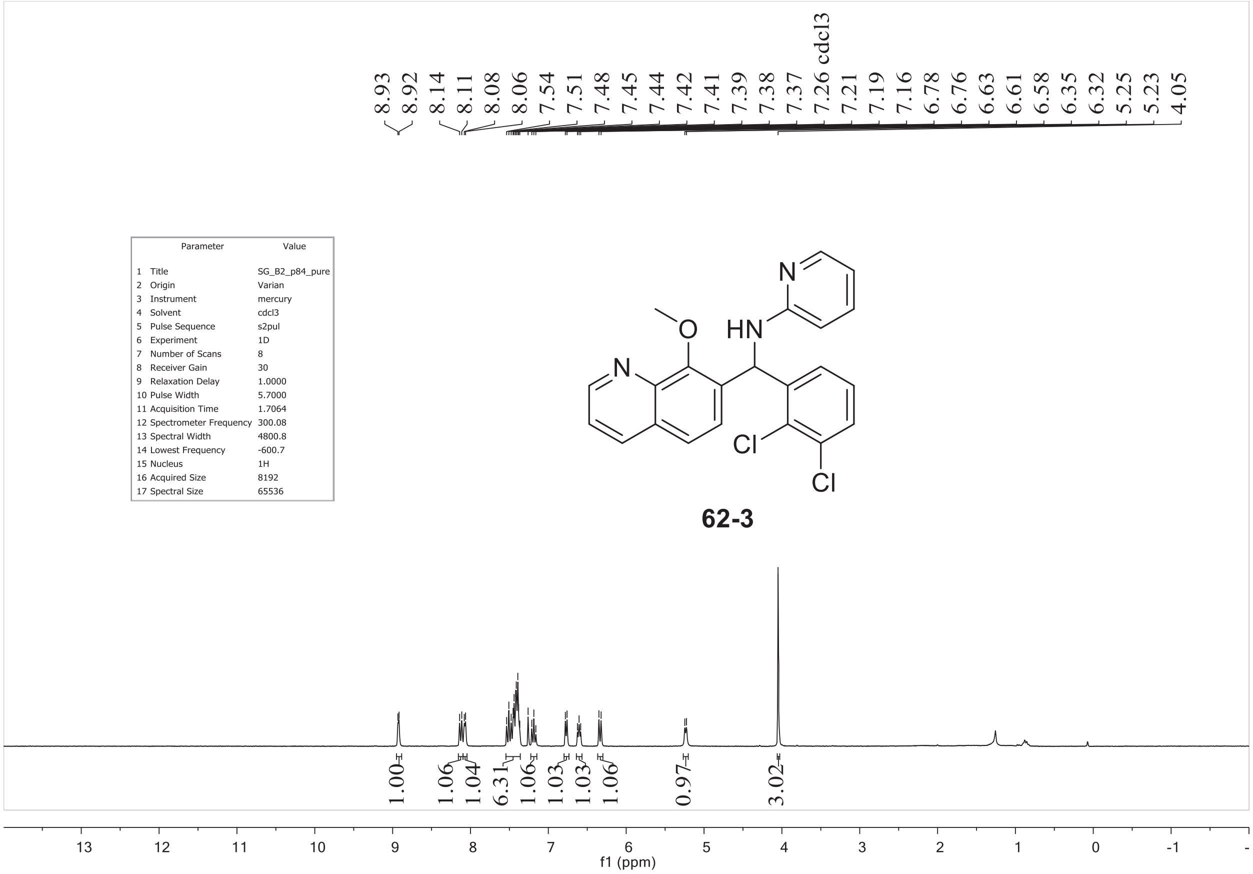
8.93
8.92
8.14
8.11
8.08
8.06
7.54
7.51
7.48
7.45
7.44
7.42
7.41
7.39
7.38
7.37
7.26 cdcl3
7.21
7.19
7.16
6.78
6.76
6.63
6.61
6.58
6.35
6.32
5.25
5.23
4.05
Parameter Value
1 Title SG_B2_p84_pure
2 Origin Varian
3 Instrument mercury
4 Solvent cdcl3
5 Pulse Sequence s2pul
6 Experiment 1D
7 Number of Scans 8
8 Receiver Gain 30
9 Relaxation Delay 1.0000
10 Pulse Width 5.7000
11 Acquisition Time 1.7064
12 Spectrometer Frequency 300.08
13 Spectral Width 4800.8
14 Lowest Frequency -600.7
15 Nucleus 1H
16 Acquired Size 8192
17 Spectral Size 65536
N
O
HN
N
Cl
Cl
62-3
1.00
1.06
1.04
6.31
1.06
1.03
1.03
1.06
0.97
3.02
13 12 11 10 9 8 7 6 5 4 3 2 1 0 -1
f1 (ppm)

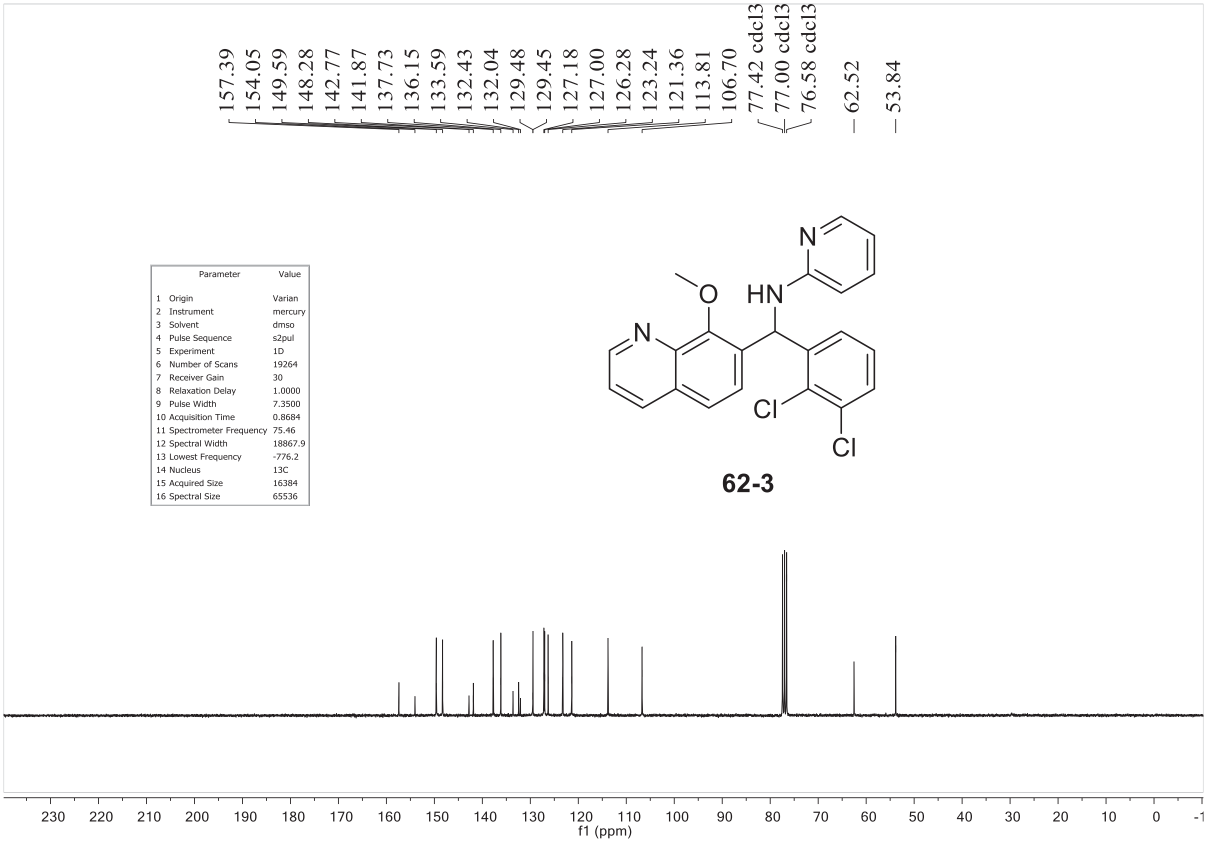
157.39
154.05
149.59
148.28
142.77
141.87
137.73
136.15
133.59
132.43
132.04
129.48
129.45
127.18
127.00
126.28
123.24
121.36
113.81
106.70
77.42 cdcl3
77.00 cdcl3
76.58 cdcl3
62.52
53.84
Parameter Value
1 Origin Varian
2 Instrument mercury
3 Solvent dmso
4 Pulse Sequence s2pul
5 Experiment 1D
6 Number of Scans 19264
7 Receiver Gain 30
8 Relaxation Delay 1.0000
9 Pulse Width 7.3500
10 Acquisition Time 0.8684
11 Spectrometer Frequency 75.46
12 Spectral Width 18867.9
13 Lowest Frequency -776.2
14 Nucleus 13C
15 Acquired Size 16384
16 Spectral Size 65536
N
O
HN
N
Cl
Cl
62-3
230 220 210 200 190 180 170 160 150 140 130 120 110 100 90 80 70 60 50 40 30 20 10 0
f1 (ppm)

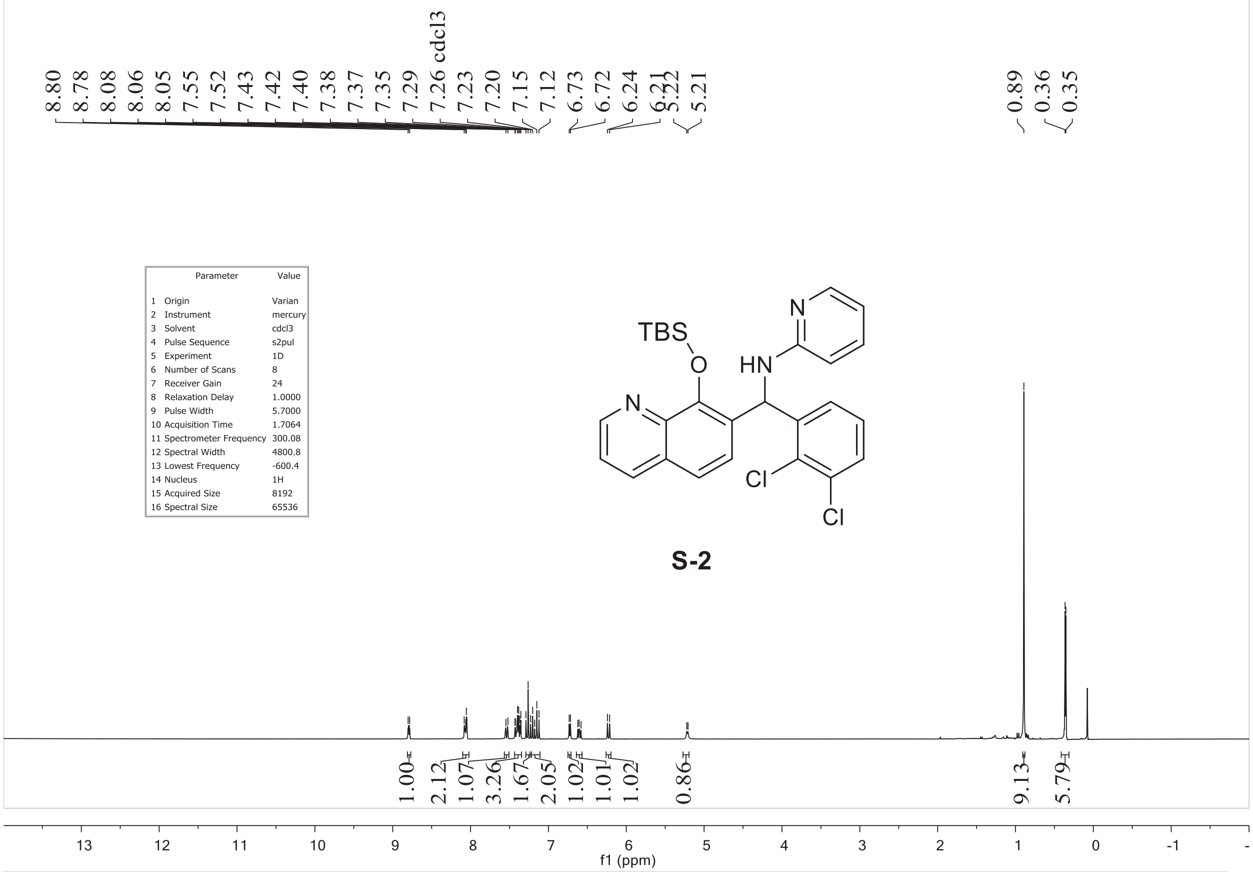
8.80 8.78 8.08 8.06 8.05 7.55 7.52 7.43 7.42 7.40 7.38 7.37 7.35 7.29 7.26 cdcl3 7.23 7.20 7.15 7.12 6.73 6.72 6.24 6.21 5.22 5.21
0.89 0.36 0.35
Parameter Value
1 Origin Varian
2 Instrument mercury
3 Solvent cdcl3
4 Pulse Sequence s2pul
5 Experiment 1D
6 Number of Scans 8
7 Receiver Gain 24
8 Relaxation Delay 1.0000
9 Pulse Width 5.7000
10 Acquisition Time 1.7064
11 Spectrometer Frequency 300.08
12 Spectral Width 4800.8
13 Lowest Frequency -600.4
14 Nucleus 1H
15 Acquired Size 8192
16 Spectral Size 65536
TBS
O
HN
N
N
Cl
Cl
S-2
1.00 2.12 1.07 3.26 1.67 2.05 1.02 1.01 1.02 0.86 9.13 5.79
13 12 11 10 9 8 7 6 5 4 3 2 1 0 -1
f1 (ppm)

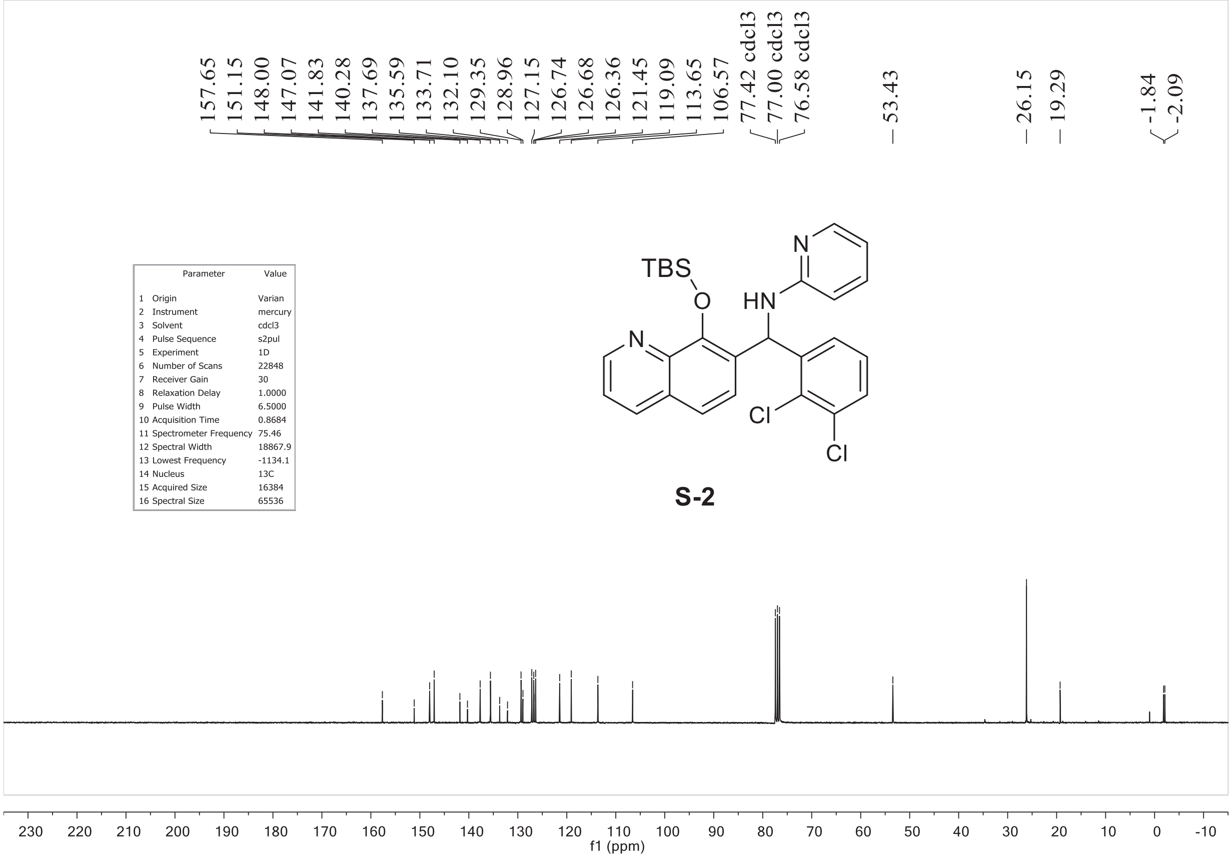
157.65 151.15 148.00 147.07 141.83 140.28 137.69 135.59 133.71 132.10 129.35 128.96 127.15 126.74 126.68 126.36 121.45 119.09 113.65 106.57
77.42 cdcl3 77.00 cdcl3 76.58 cdcl3
53.43
26.15 19.29
-1.84 -2.09
Parameter Value
1 Origin Varian
2 Instrument mercury
3 Solvent cdcl3
4 Pulse Sequence s2pul
5 Experiment 1D
6 Number of Scans 22848
7 Receiver Gain 30
8 Relaxation Delay 1.0000
9 Pulse Width 6.5000
10 Acquisition Time 0.8684
11 Spectrometer Frequency 75.46
12 Spectral Width 18867.9
13 Lowest Frequency -1134.1
14 Nucleus 13C
15 Acquired Size 16384
16 Spectral Size 65536
TBS
O
HN
N
N
Cl
Cl
S-2
230 220 210 200 190 180 170 160 150 140 130 120 110 100 90 80 70 60 50 40 30 20 10 0 -10
f1 (ppm)

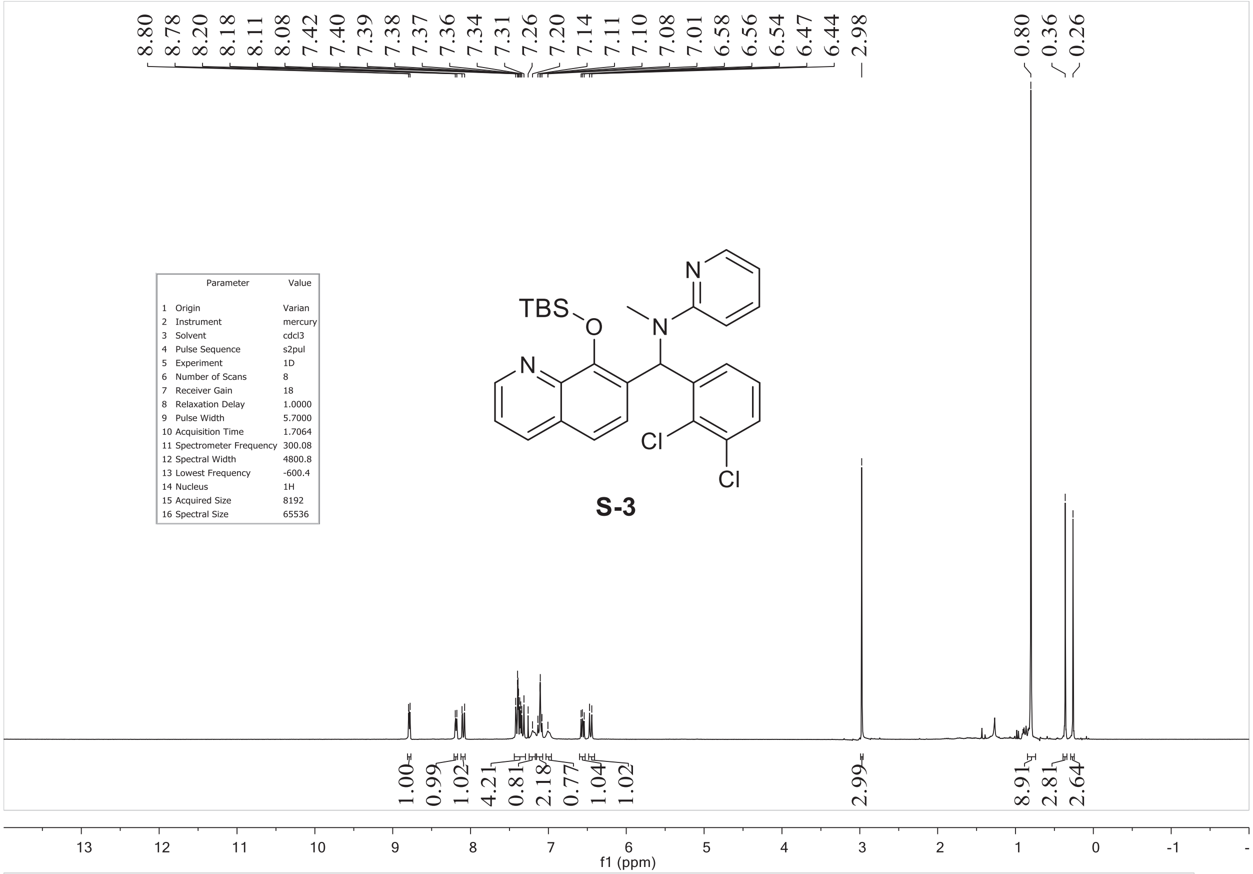
8.80 8.78 8.20 8.18 8.11 8.08 7.42 7.40 7.39 7.38 7.37 7.36 7.34 7.31 7.26 7.20 7.14 7.11 7.10 7.08 7.01 6.58 6.56 6.54 6.47 6.44 2.98 0.80 0.36 0.26
1.00 0.99 1.02 4.21 0.81 2.18 0.77 1.04 1.02 2.99 8.91 2.81 2.64
Parameter Value
1 Origin Varian
2 Instrument mercury
3 Solvent cdcl3
4 Pulse Sequence s2pul
5 Experiment 1D
6 Number of Scans 8
7 Receiver Gain 18
8 Relaxation Delay 1.0000
9 Pulse Width 5.7000
10 Acquisition Time 1.7064
11 Spectrometer Frequency 300.08
12 Spectral Width 4800.8
13 Lowest Frequency -600.4
14 Nucleus 1H
15 Acquired Size 8192
16 Spectral Size 65536
TBS
O
N
N
N
Cl
Cl
S-3
f1 (ppm)

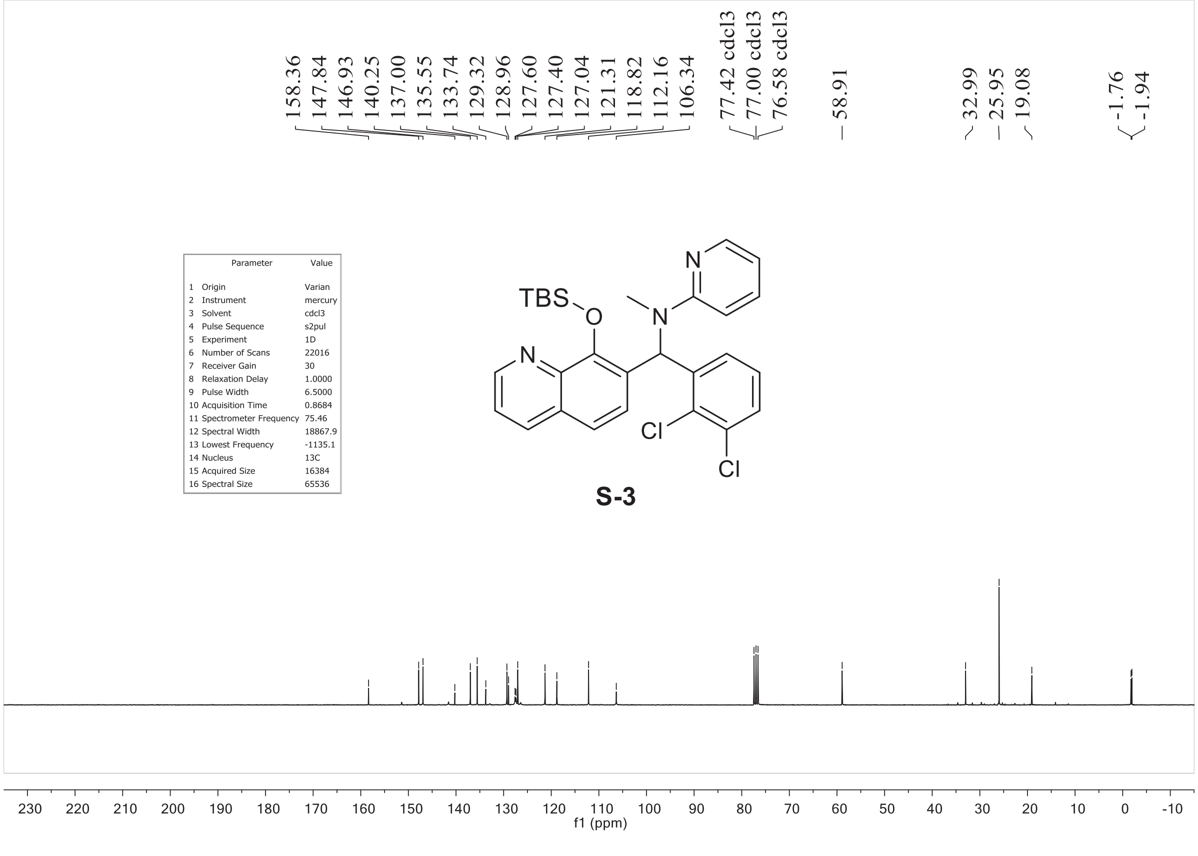
158.36 147.84 146.93 140.25 137.00 135.55 133.74 129.32 128.96 127.60 127.40 127.04 121.31 118.82 112.16 106.34
77.42 cdcl3 77.00 cdcl3 76.58 cdcl3
58.91 32.99 25.95 19.08 -1.76 -1.94
Parameter Value
1 Origin Varian
2 Instrument mercury
3 Solvent cdcl3
4 Pulse Sequence s2pul
5 Experiment 1D
6 Number of Scans 22016
7 Receiver Gain 30
8 Relaxation Delay 1.0000
9 Pulse Width 6.5000
10 Acquisition Time 0.8684
11 Spectrometer Frequency 75.46
12 Spectral Width 18867.9
13 Lowest Frequency -1135.1
14 Nucleus 13C
15 Acquired Size 16384
16 Spectral Size 65536
TBS
O
N
N
N
Cl
Cl
S-3
f1 (ppm)

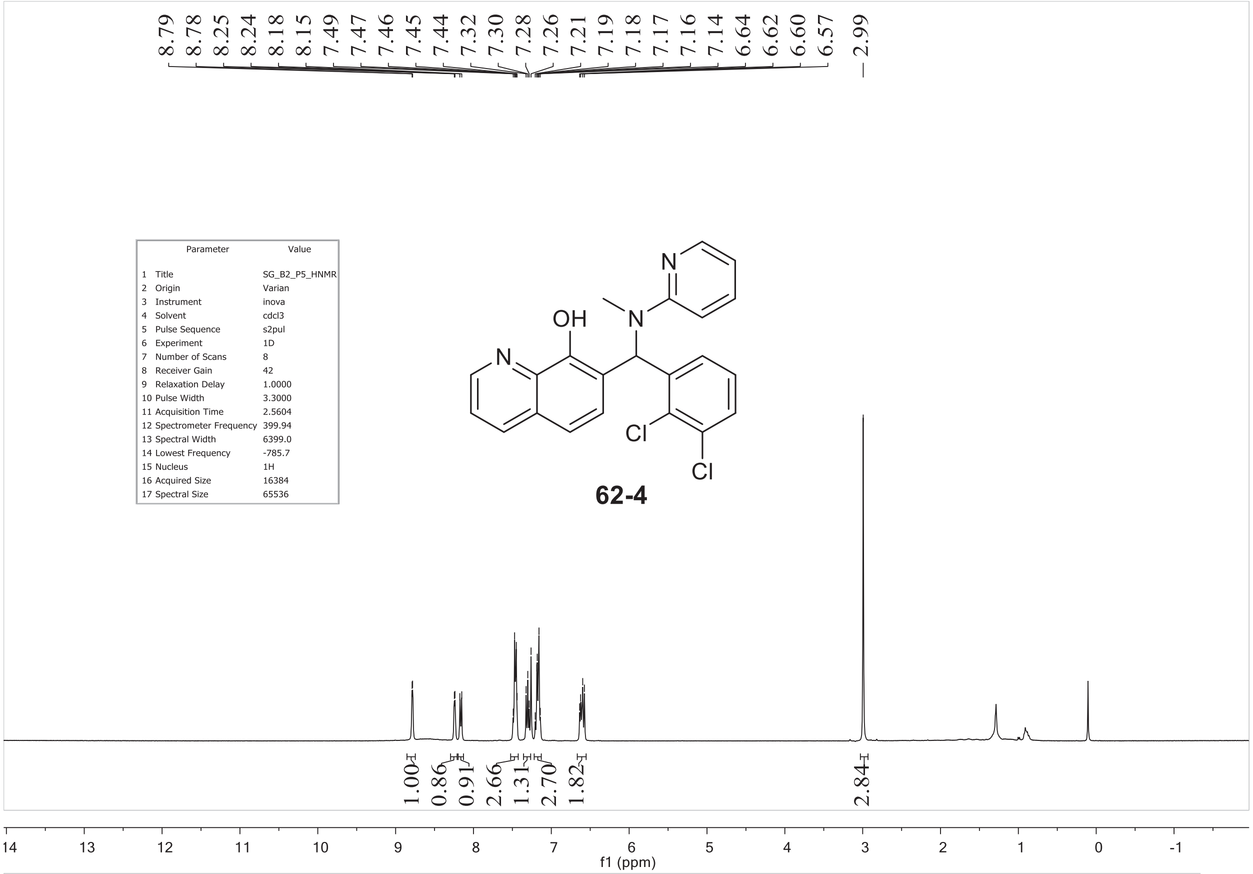
8.79 8.78 8.25 8.24 8.18 8.15 7.49 7.47 7.46 7.45 7.44 7.32 7.30 7.28 7.26 7.21 7.19 7.18 7.17 7.16 7.14 6.64 6.62 6.60 6.57
2.99
Parameter Value
1 Title SG_B2_P5_HNMR
2 Origin Varian
3 Instrument inova
4 Solvent cdcl3
5 Pulse Sequence s2pul
6 Experiment 1D
7 Number of Scans 8
8 Receiver Gain 42
9 Relaxation Delay 1.0000
10 Pulse Width 3.3000
11 Acquisition Time 2.5604
12 Spectrometer Frequency 399.94
13 Spectral Width 6399.0
14 Lowest Frequency -785.7
15 Nucleus 1H
16 Acquired Size 16384
17 Spectral Size 65536
OH
N
N
N
Cl
Cl
62-4
1.00 0.86 0.91 2.66 1.31 2.70 1.82
2.84
f1 (ppm)

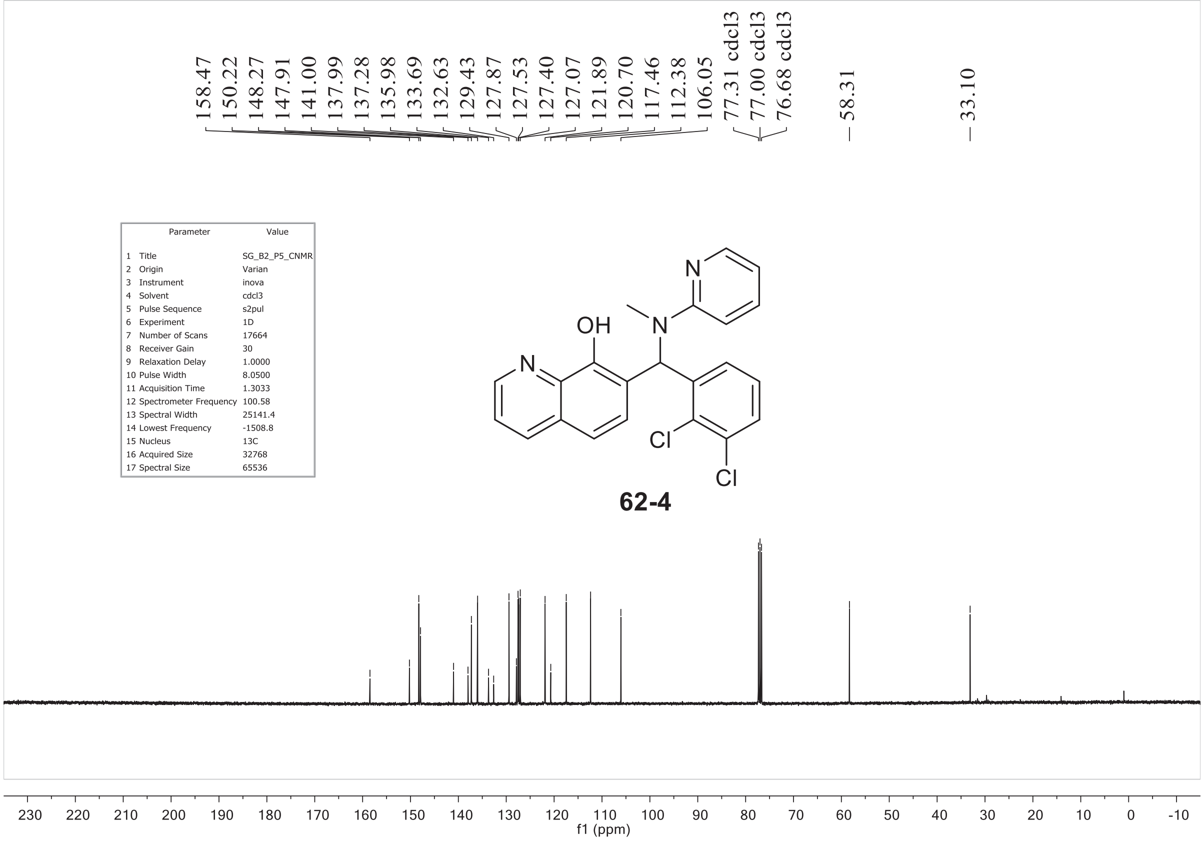
158.47 150.22 148.27 147.91 141.00 137.99 137.28 135.98 133.69 132.63 129.43 127.87 127.53 127.40 127.07 121.89 120.70 117.46 112.38 106.05
77.31 cdcl3
77.00 cdcl3
76.68 cdcl3
58.31
33.10
Parameter Value
1 Title SG_B2_P5_CNMR
2 Origin Varian
3 Instrument inova
4 Solvent cdcl3
5 Pulse Sequence s2pul
6 Experiment 1D
7 Number of Scans 17664
8 Receiver Gain 30
9 Relaxation Delay 1.0000
10 Pulse Width 8.0500
11 Acquisition Time 1.3033
12 Spectrometer Frequency 100.58
13 Spectral Width 25141.4
14 Lowest Frequency -1508.8
15 Nucleus 13C
16 Acquired Size 32768
17 Spectral Size 65536
OH
N
N
N
Cl
Cl
62-4
f1 (ppm)

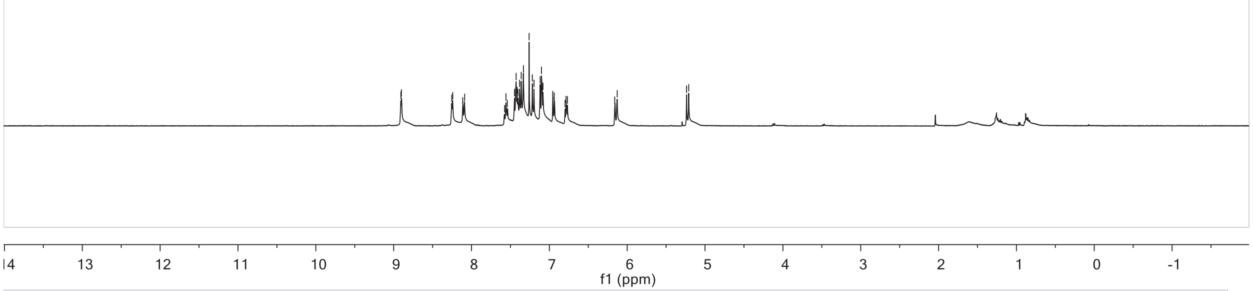

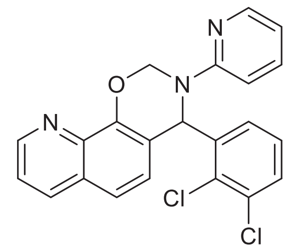

62-5

Parameter	Value
1 Origin	Varian
2 Instrument	inova
3 Solvent	cdcl3
4 Pulse Sequence	s2pul
5 Experiment	1D
6 Number of Scans	8
7 Receiver Gain	48
8 Relaxation Delay	1.0000
9 Pulse Width	3.3000
10 Acquisition Time	2.5604
11 Spectrometer Frequency	399.94
12 Spectral Width	6399.0
13 Lowest Frequency	-795.7
14 Nucleus	1H
15 Acquired Size	16384
16 Spectral Size	65536

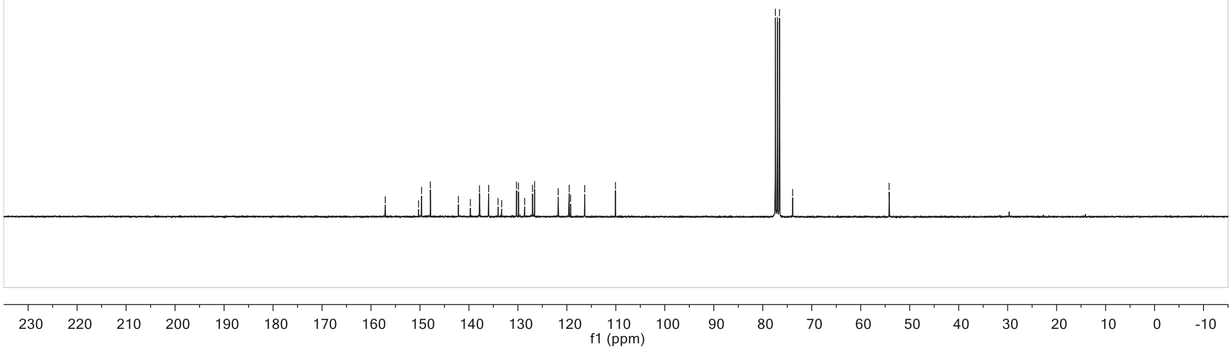

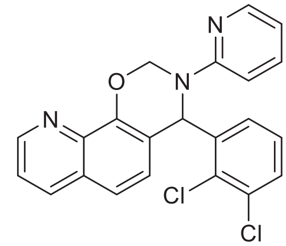

62-5

Parameter	Value
1 Origin	Varian
2 Instrument	mercury
3 Solvent	cdcl3
4 Pulse Sequence	s2pul
5 Experiment	1D
6 Number of Scans	23040
7 Receiver Gain	30
8 Relaxation Delay	1.0000
9 Pulse Width	6.5000
10 Acquisition Time	0.8684
11 Spectrometer Frequency	75.46
12 Spectral Width	18867.9
13 Lowest Frequency	-1134.4
14 Nucleus	13C
15 Acquired Size	16384
16 Spectral Size	32768

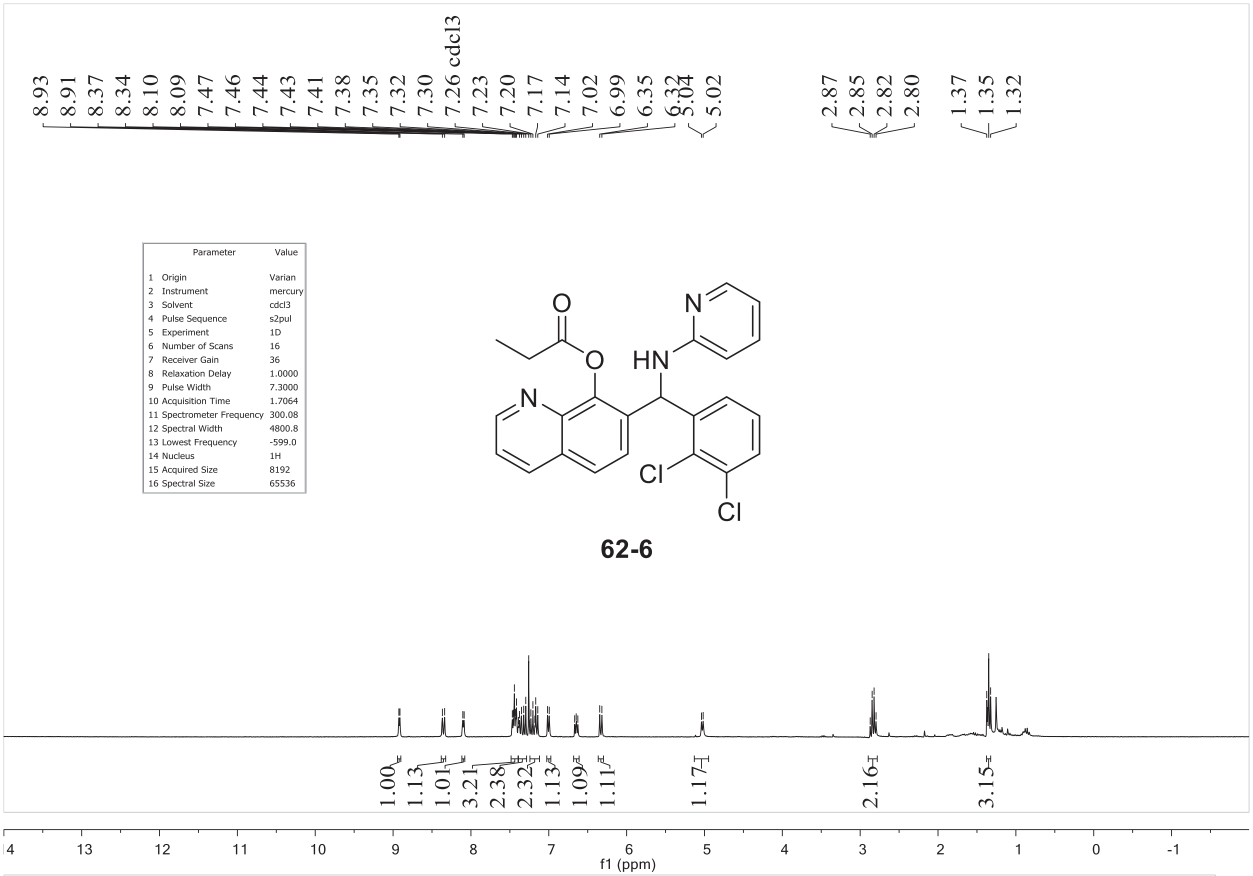
8.93 8.91 8.37 8.34 8.10 8.09 7.47 7.46 7.44 7.43 7.41 7.38 7.35 7.32 7.30 7.26 cdcl3 7.23 7.20 7.17 7.14 7.02 6.99 6.35 6.32 5.04 5.02
2.87 2.85 2.82 2.80
1.37 1.35 1.32
Parameter Value
1 Origin Varian
2 Instrument mercury
3 Solvent cdcl3
4 Pulse Sequence s2pul
5 Experiment 1D
6 Number of Scans 16
7 Receiver Gain 36
8 Relaxation Delay 1.0000
9 Pulse Width 7.3000
10 Acquisition Time 1.7064
11 Spectrometer Frequency 300.08
12 Spectral Width 4800.8
13 Lowest Frequency -599.0
14 Nucleus 1H
15 Acquired Size 8192
16 Spectral Size 65536
O
N
HN
O
N
Cl
Cl
62-6
1.00 1.13 1.01 3.21 2.38 2.32 1.13 1.09 1.11 1.17 2.16 3.15
f1 (ppm)

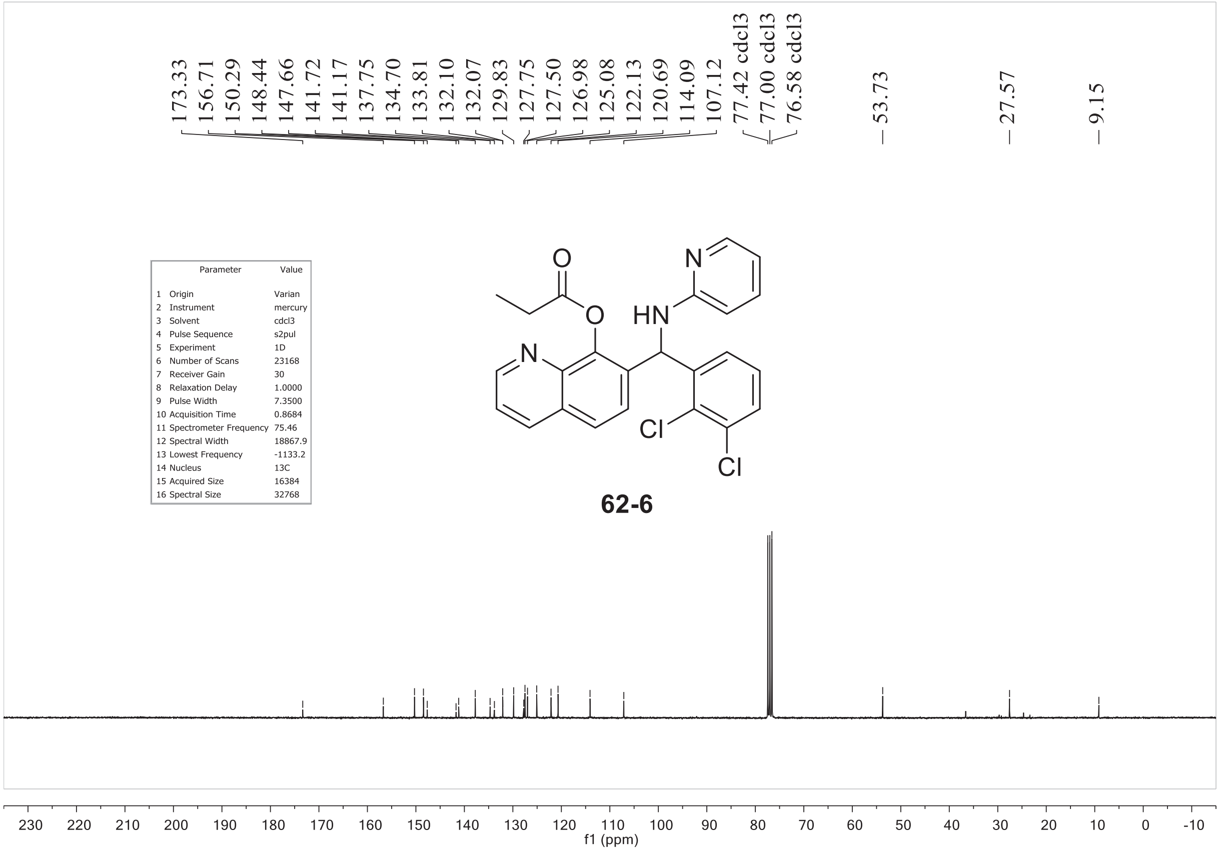
173.33 156.71 150.29 148.44 147.66 141.72 141.17 137.75 134.70 133.81 132.10 132.07 129.83 127.75 127.50 126.98 125.08 122.13 120.69 114.09 107.12 77.42 cdcl3 77.00 cdcl3 76.58 cdcl3
53.73
27.57
9.15
Parameter Value
1 Origin Varian
2 Instrument mercury
3 Solvent cdcl3
4 Pulse Sequence s2pul
5 Experiment 1D
6 Number of Scans 23168
7 Receiver Gain 30
8 Relaxation Delay 1.0000
9 Pulse Width 7.3500
10 Acquisition Time 0.8684
11 Spectrometer Frequency 75.46
12 Spectral Width 18867.9
13 Lowest Frequency -1133.2
14 Nucleus 13C
15 Acquired Size 16384
16 Spectral Size 32768
O
N
HN
O
N
Cl
Cl
62-6
f1 (ppm)

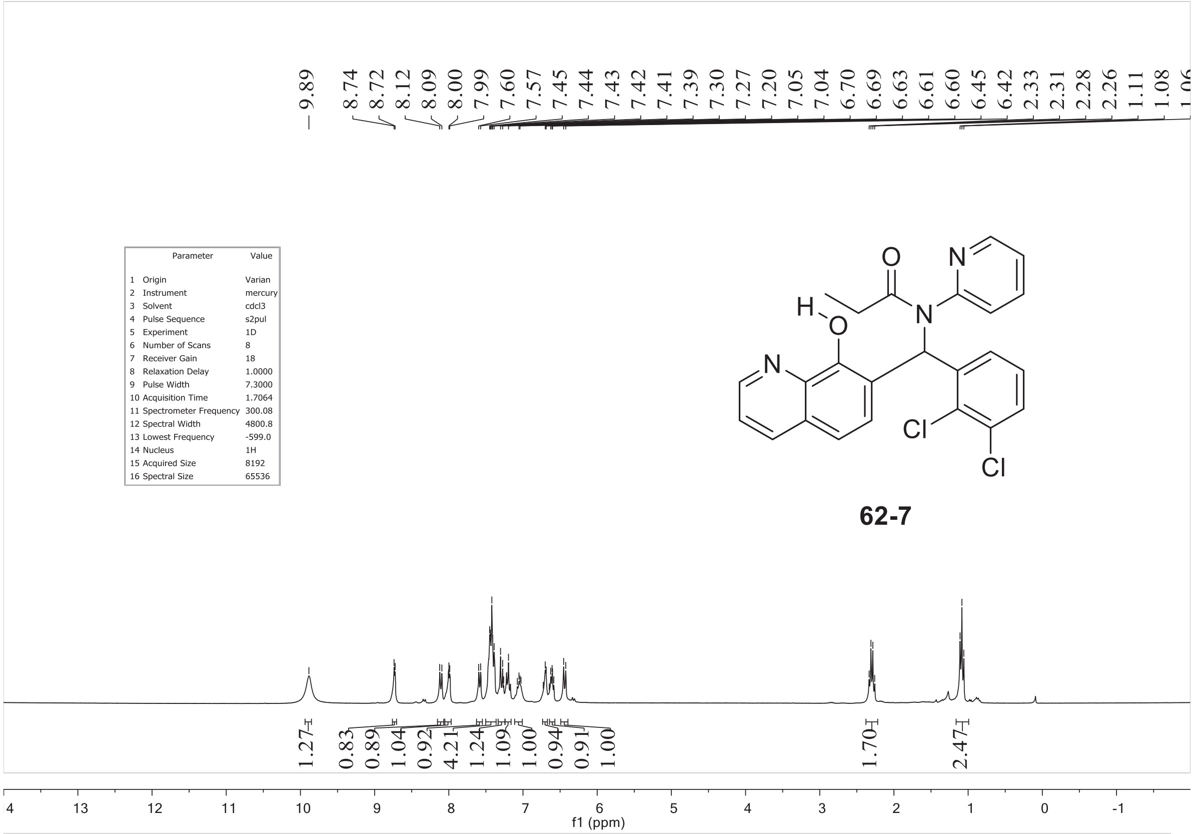
9.89
8.74 8.72 8.12 8.09 8.00 7.99 7.60 7.57 7.45 7.44 7.43 7.42 7.41 7.39 7.30 7.27 7.20 7.05 7.04 6.70 6.69 6.63 6.61 6.60 6.45 6.42 2.33 2.31 2.28 2.26 1.11 1.08 1.06
Parameter Value
1 Origin Varian
2 Instrument mercury
3 Solvent cdcl3
4 Pulse Sequence s2pul
5 Experiment 1D
6 Number of Scans 8
7 Receiver Gain 18
8 Relaxation Delay 1.0000
9 Pulse Width 7.3000
10 Acquisition Time 1.7064
11 Spectrometer Frequency 300.08
12 Spectral Width 4800.8
13 Lowest Frequency -599.0
14 Nucleus 1H
15 Acquired Size 8192
16 Spectral Size 65536
O
N
H
O
N
N
Cl
Cl
62-7
1.27 0.83 0.89 1.04 0.92 4.21 1.24 1.09 1.00 0.94 0.91 1.00 1.70 2.47
4 13 12 11 10 9 8 7 6 5 4 3 2 1 0 -1
f1 (ppm)

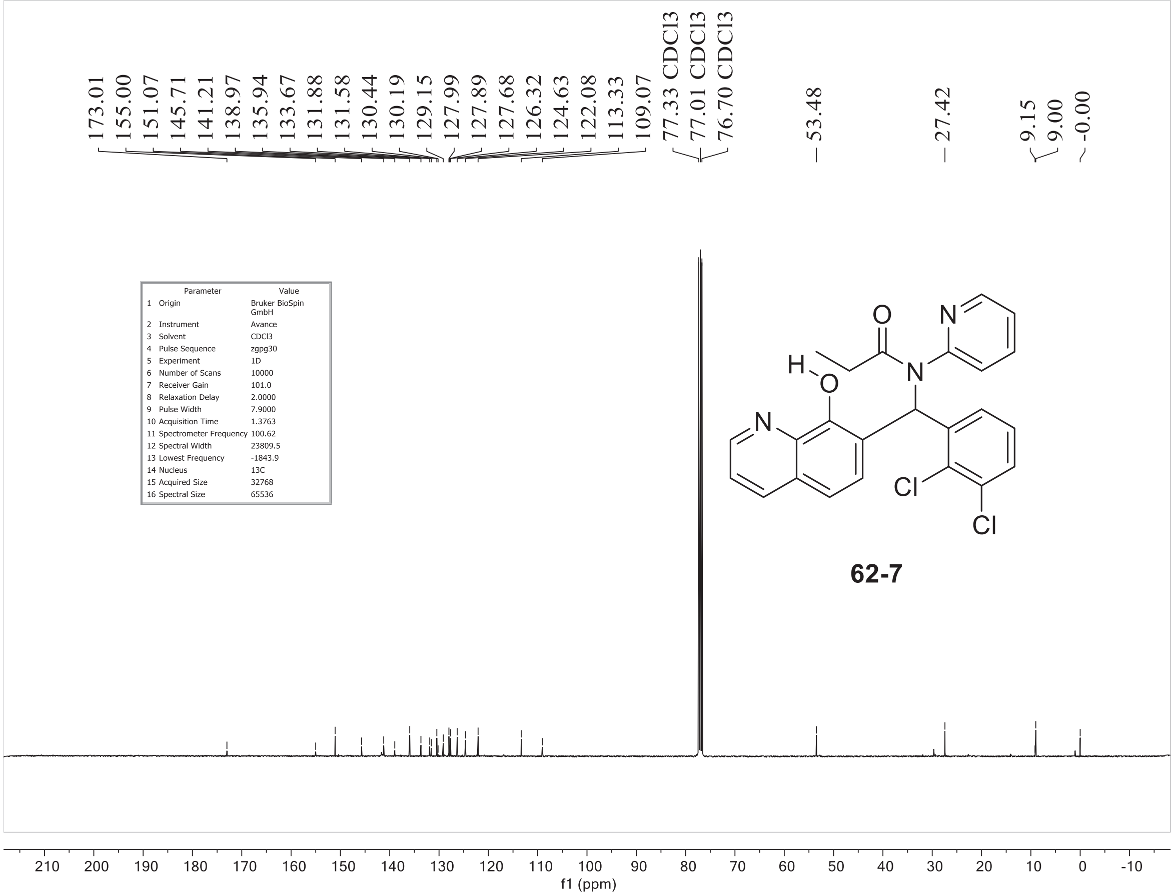
173.01 155.00 151.07 145.71 141.21 138.97 135.94 133.67 131.88 131.58 130.44 130.19 129.15 127.99 127.89 127.68 126.32 124.63 122.08 113.33 109.07
77.33 CDCl3 77.01 CDCl3 76.70 CDCl3
53.48
27.42
9.15 9.00 -0.00
Parameter Value
1 Origin Bruker BioSpin GmbH
2 Instrument Avance
3 Solvent CDCl3
4 Pulse Sequence zgpg30
5 Experiment 1D
6 Number of Scans 10000
7 Receiver Gain 101.0
8 Relaxation Delay 2.0000
9 Pulse Width 7.9000
10 Acquisition Time 1.3763
11 Spectrometer Frequency 100.62
12 Spectral Width 23809.5
13 Lowest Frequency -1843.9
14 Nucleus 13C
15 Acquired Size 32768
16 Spectral Size 65536
O
N
H
O
N
N
Cl
Cl
62-7
210 200 190 180 170 160 150 140 130 120 110 100 90 80 70 60 50 40 30 20 10 0 -10
f1 (ppm)

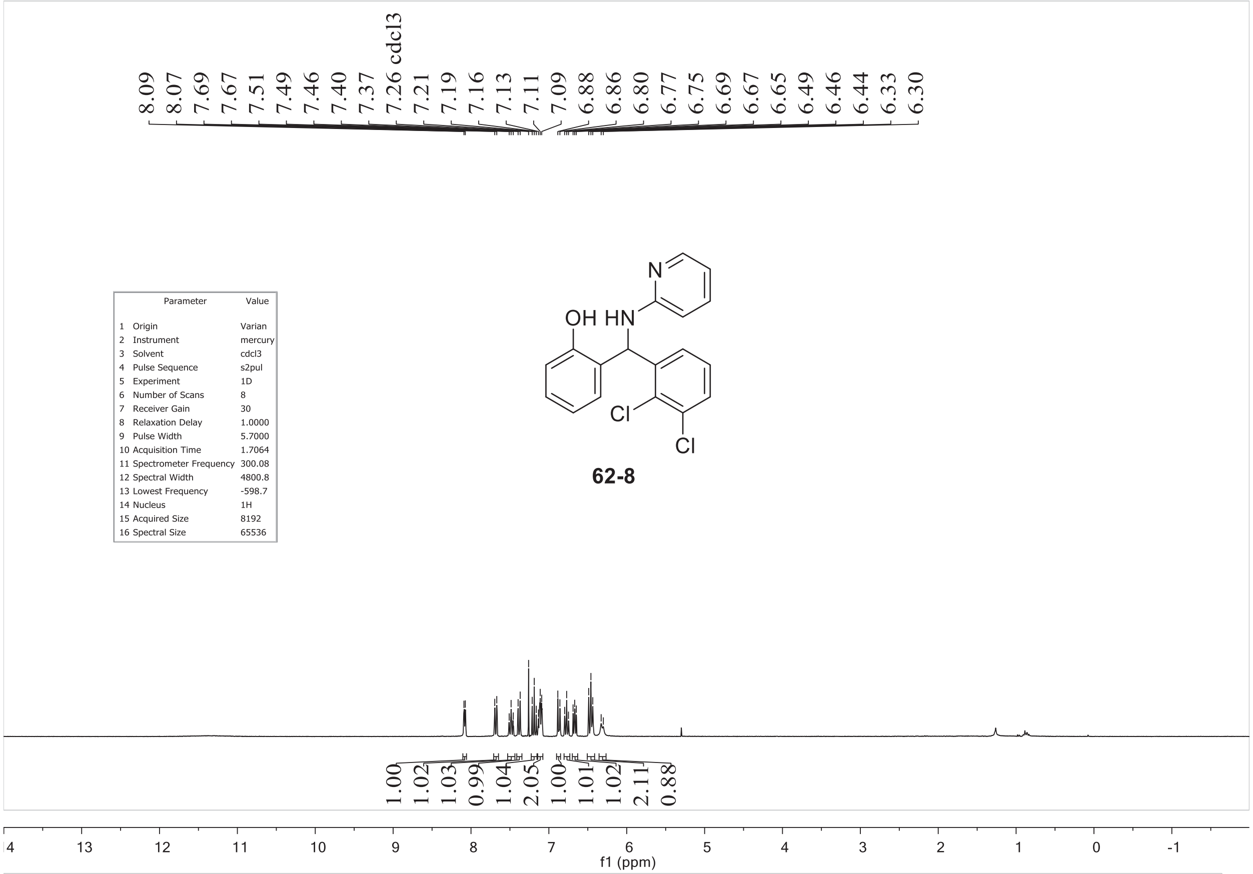

Parameter	Value
1 Origin	Varian
2 Instrument	mercury
3 Solvent	cdcl3
4 Pulse Sequence	s2pul
5 Experiment	1D
6 Number of Scans	8
7 Receiver Gain	30
8 Relaxation Delay	1.0000
9 Pulse Width	5.7000
10 Acquisition Time	1.7064
11 Spectrometer Frequency	300.08
12 Spectral Width	4800.8
13 Lowest Frequency	-598.7
14 Nucleus	1H
15 Acquired Size	8192
16 Spectral Size	65536

62-8

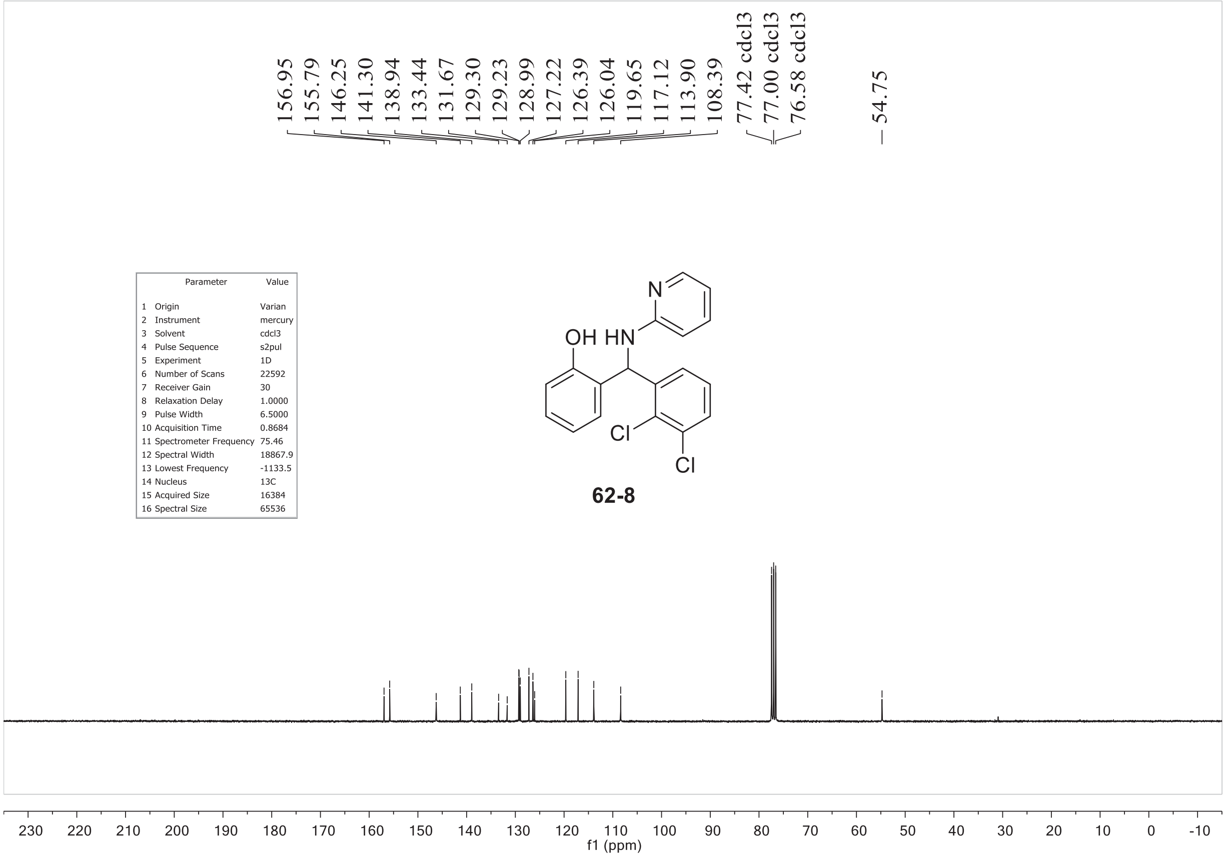

Parameter	Value
1 Origin	Varian
2 Instrument	mercury
3 Solvent	cdcl3
4 Pulse Sequence	s2pul
5 Experiment	1D
6 Number of Scans	22592
7 Receiver Gain	30
8 Relaxation Delay	1.0000
9 Pulse Width	6.5000
10 Acquisition Time	0.8684
11 Spectrometer Frequency	75.46
12 Spectral Width	18867.9
13 Lowest Frequency	-1133.5
14 Nucleus	13C
15 Acquired Size	16384
16 Spectral Size	65536

62-8

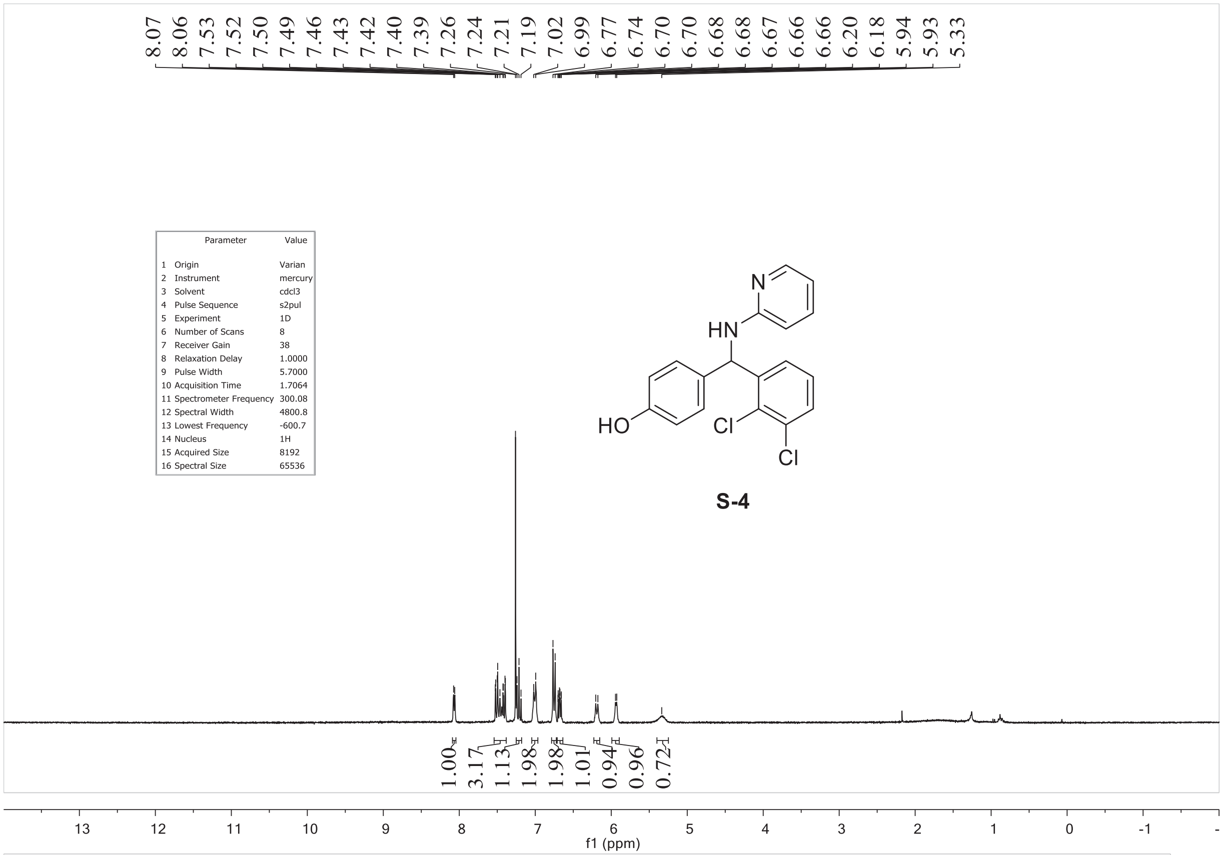

8.07 8.06 7.53 7.52 7.50 7.49 7.46 7.43 7.42 7.40 7.39 7.26 7.24 7.21 7.19 7.02 6.99 6.77 6.74 6.70 6.70 6.68 6.68 6.67 6.66 6.66 6.20 6.18 5.94 5.93 5.33
Parameter Value
1 Origin Varian
2 Instrument mercury
3 Solvent cdcl3
4 Pulse Sequence s2pul
5 Experiment 1D
6 Number of Scans 8
7 Receiver Gain 38
8 Relaxation Delay 1.0000
9 Pulse Width 5.7000
10 Acquisition Time 1.7064
11 Spectrometer Frequency 300.08
12 Spectral Width 4800.8
13 Lowest Frequency -600.7
14 Nucleus 1H
15 Acquired Size 8192
16 Spectral Size 65536
N
HN
HO
Cl
Cl
S-4
1.00 3.17 1.13 1.98 1.98 1.01 0.94 0.96 0.72
f1 (ppm)

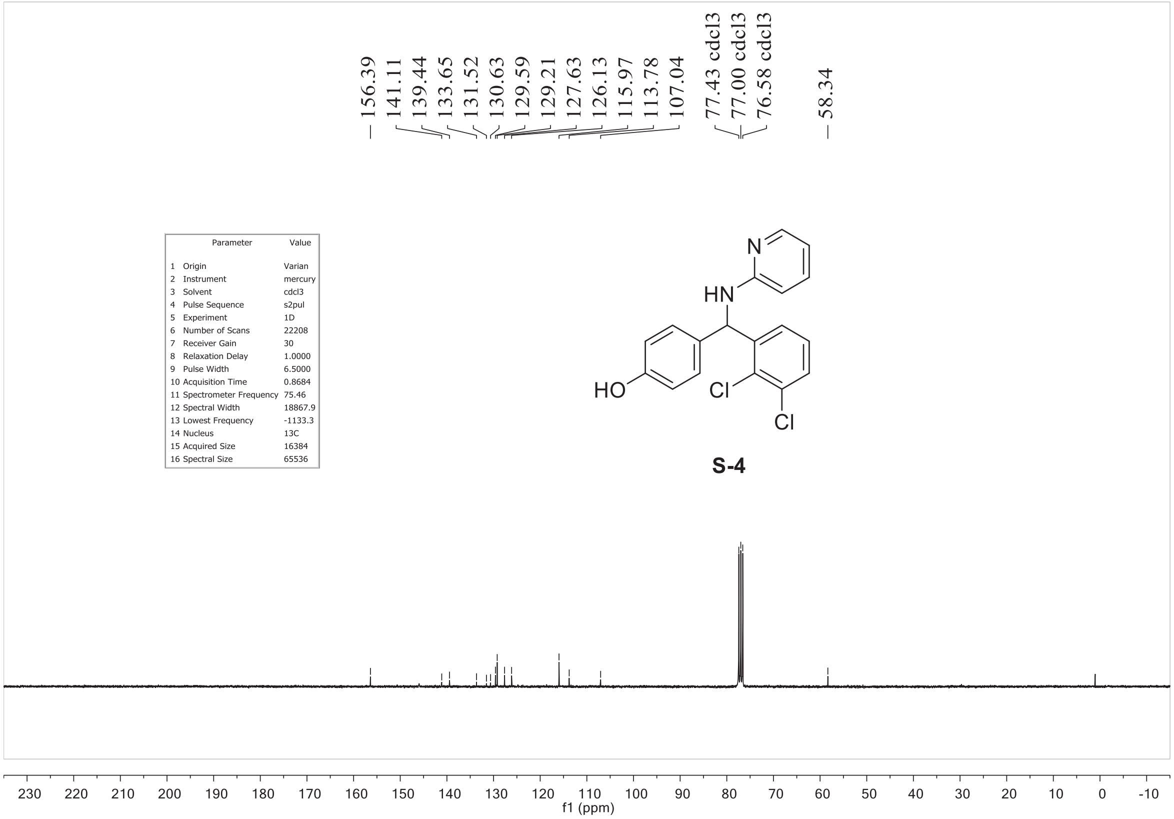

156.39 141.11 139.44 133.65 131.52 130.63 129.59 129.21 127.63 126.13 115.97 113.78 107.04
77.43 cdcl3 77.00 cdcl3 76.58 cdcl3
58.34
Parameter Value
1 Origin Varian
2 Instrument mercury
3 Solvent cdcl3
4 Pulse Sequence s2pul
5 Experiment 1D
6 Number of Scans 22208
7 Receiver Gain 30
8 Relaxation Delay 1.0000
9 Pulse Width 6.5000
10 Acquisition Time 0.8684
11 Spectrometer Frequency 75.46
12 Spectral Width 18867.9
13 Lowest Frequency -1133.3
14 Nucleus 13C
15 Acquired Size 16384
16 Spectral Size 65536
N
HN
HO
Cl
Cl
S-4
f1 (ppm)

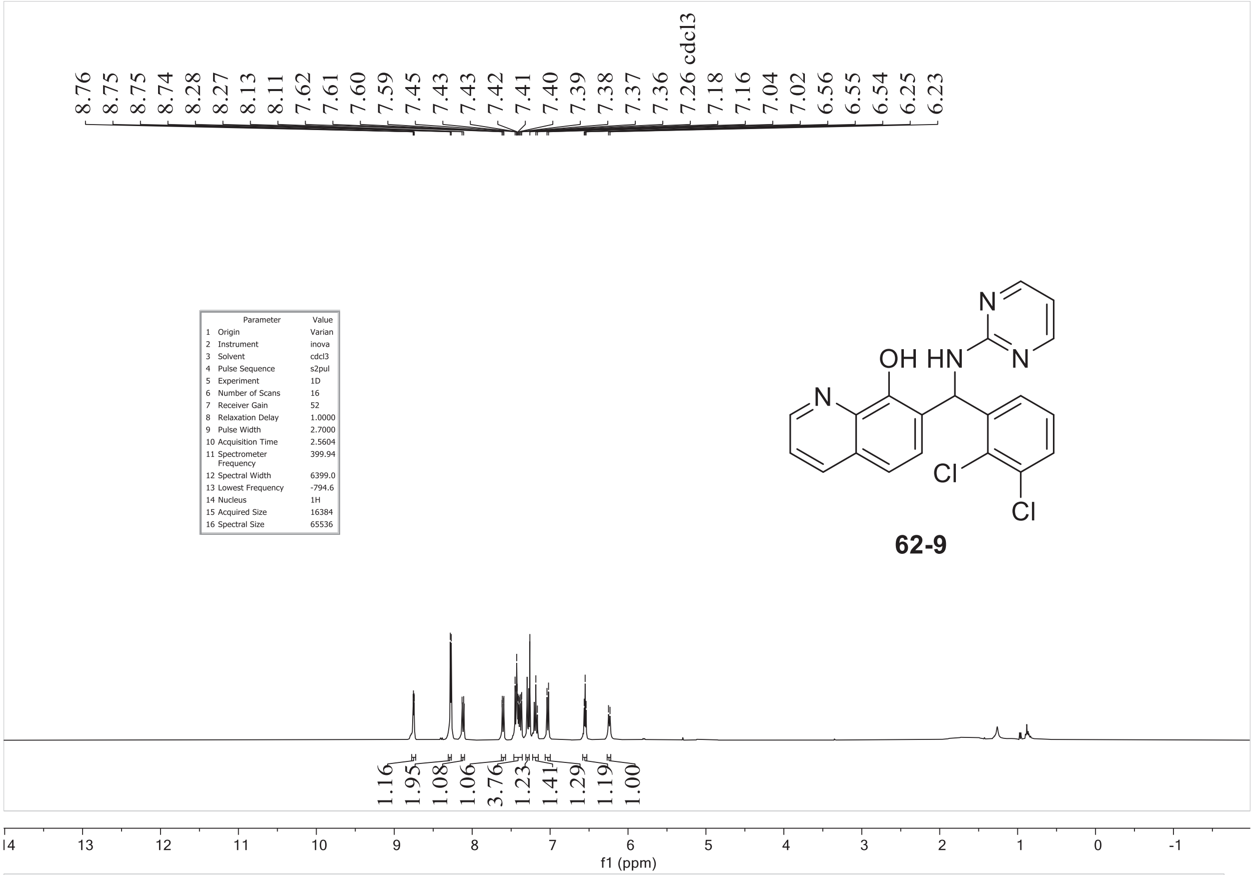
8.76 8.75 8.75 8.74 8.28 8.27 8.13 8.11 7.62 7.61 7.60 7.59 7.45 7.43 7.43 7.42 7.41 7.40 7.39 7.38 7.37 7.36 7.26 cdcl3 7.18 7.16 7.04 7.02 6.56 6.55 6.54 6.25 6.23
Parameter Value
1 Origin Varian
2 Instrument inova
3 Solvent cdcl3
4 Pulse Sequence s2pul
5 Experiment 1D
6 Number of Scans 16
7 Receiver Gain 52
8 Relaxation Delay 1.0000
9 Pulse Width 2.7000
10 Acquisition Time 2.5604
11 Spectrometer Frequency 399.94
12 Spectral Width 6399.0
13 Lowest Frequency -794.6
14 Nucleus 1H
15 Acquired Size 16384
16 Spectral Size 65536
OH HN N N N Cl Cl
62-9
1.16 1.95 1.08 1.06 3.76 1.23 1.41 1.29 1.19 1.00
14 13 12 11 10 9 8 7 6 5 4 3 2 1 0 -1
f1 (ppm)

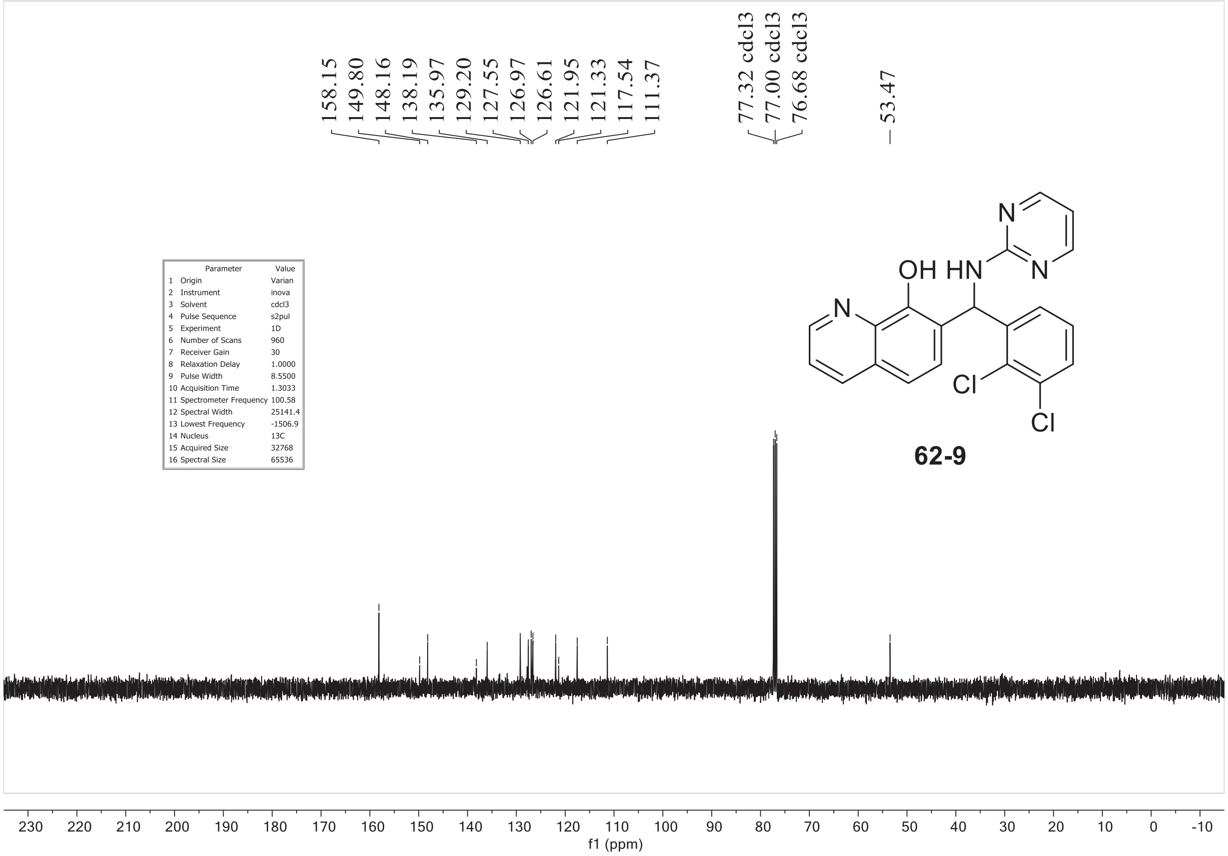
158.15 149.80 148.16 138.19 135.97 129.20 127.55 126.97 126.61 121.95 121.33 117.54 111.37
77.32 cdcl3 77.00 cdcl3 76.68 cdcl3
53.47
Parameter Value
1 Origin Varian
2 Instrument inova
3 Solvent cdcl3
4 Pulse Sequence s2pul
5 Experiment 1D
6 Number of Scans 960
7 Receiver Gain 30
8 Relaxation Delay 1.0000
9 Pulse Width 8.5500
10 Acquisition Time 1.3033
11 Spectrometer Frequency 100.58
12 Spectral Width 25141.4
13 Lowest Frequency -1506.9
14 Nucleus 13C
15 Acquired Size 32768
16 Spectral Size 65536
OH HN N N N Cl Cl
62-9
230 220 210 200 190 180 170 160 150 140 130 120 110 100 90 80 70 60 50 40 30 20 10 0 -10
f1 (ppm)

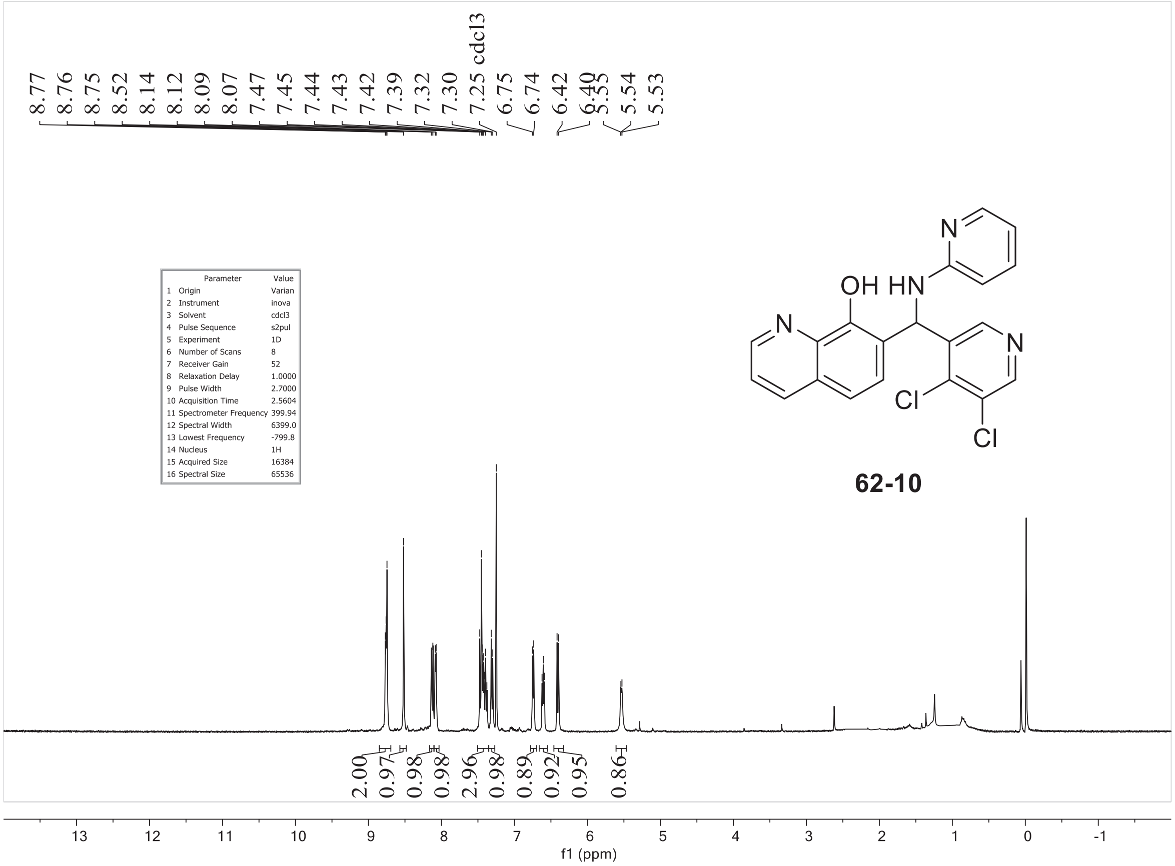
8.77
8.76
8.75
8.52
8.14
8.12
8.09
8.07
7.47
7.45
7.44
7.43
7.42
7.39
7.32
7.30
7.25 cdcl3
6.75
6.74
6.42
6.40
5.55
5.54
5.53
Parameter Value
1 Origin Varian
2 Instrument inova
3 Solvent cdcl3
4 Pulse Sequence s2pul
5 Experiment 1D
6 Number of Scans 8
7 Receiver Gain 52
8 Relaxation Delay 1.0000
9 Pulse Width 2.7000
10 Acquisition Time 2.5604
11 Spectrometer Frequency 399.94
12 Spectral Width 6399.0
13 Lowest Frequency -799.8
14 Nucleus 1H
15 Acquired Size 16384
16 Spectral Size 65536
OH
HN
N
N
N
Cl
Cl
62-10
2.00
0.97
0.98
0.98
2.96
0.98
0.89
0.92
0.95
0.86
13 12 11 10 9 8 7 6 5 4 3 2 1 0 -1
f1 (ppm)

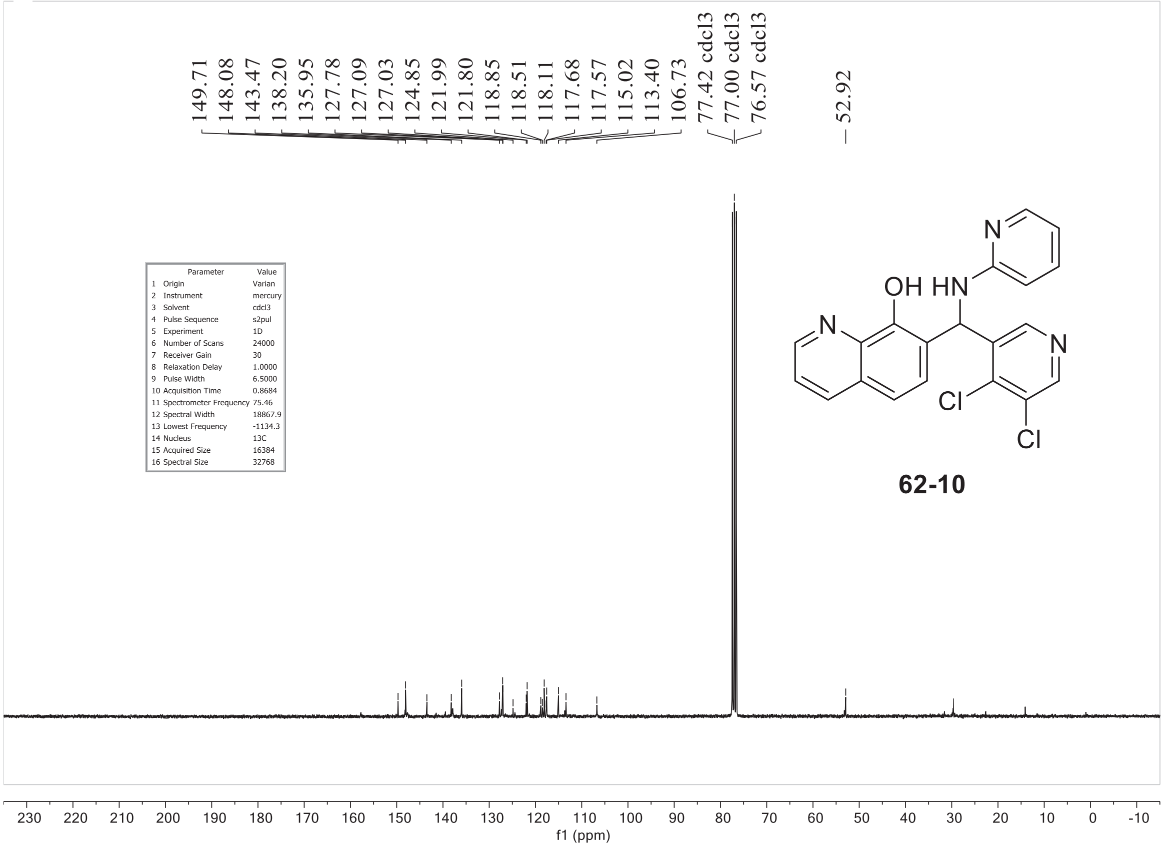
149.71
148.08
143.47
138.20
135.95
127.78
127.09
127.03
124.85
121.99
121.80
118.85
118.51
118.11
117.68
117.57
115.02
113.40
106.73
77.42 cdcl3
77.00 cdcl3
76.57 cdcl3
52.92
Parameter Value
1 Origin Varian
2 Instrument mercury
3 Solvent cdcl3
4 Pulse Sequence s2pul
5 Experiment 1D
6 Number of Scans 24000
7 Receiver Gain 30
8 Relaxation Delay 1.0000
9 Pulse Width 6.5000
10 Acquisition Time 0.8684
11 Spectrometer Frequency 75.46
12 Spectral Width 18867.9
13 Lowest Frequency -1134.3
14 Nucleus 13C
15 Acquired Size 16384
16 Spectral Size 32768
OH
HN
N
N
N
Cl
Cl
62-10
230 220 210 200 190 180 170 160 150 140 130 120 110 100 90 80 70 60 50 40 30 20 10 0 -10
f1 (ppm)

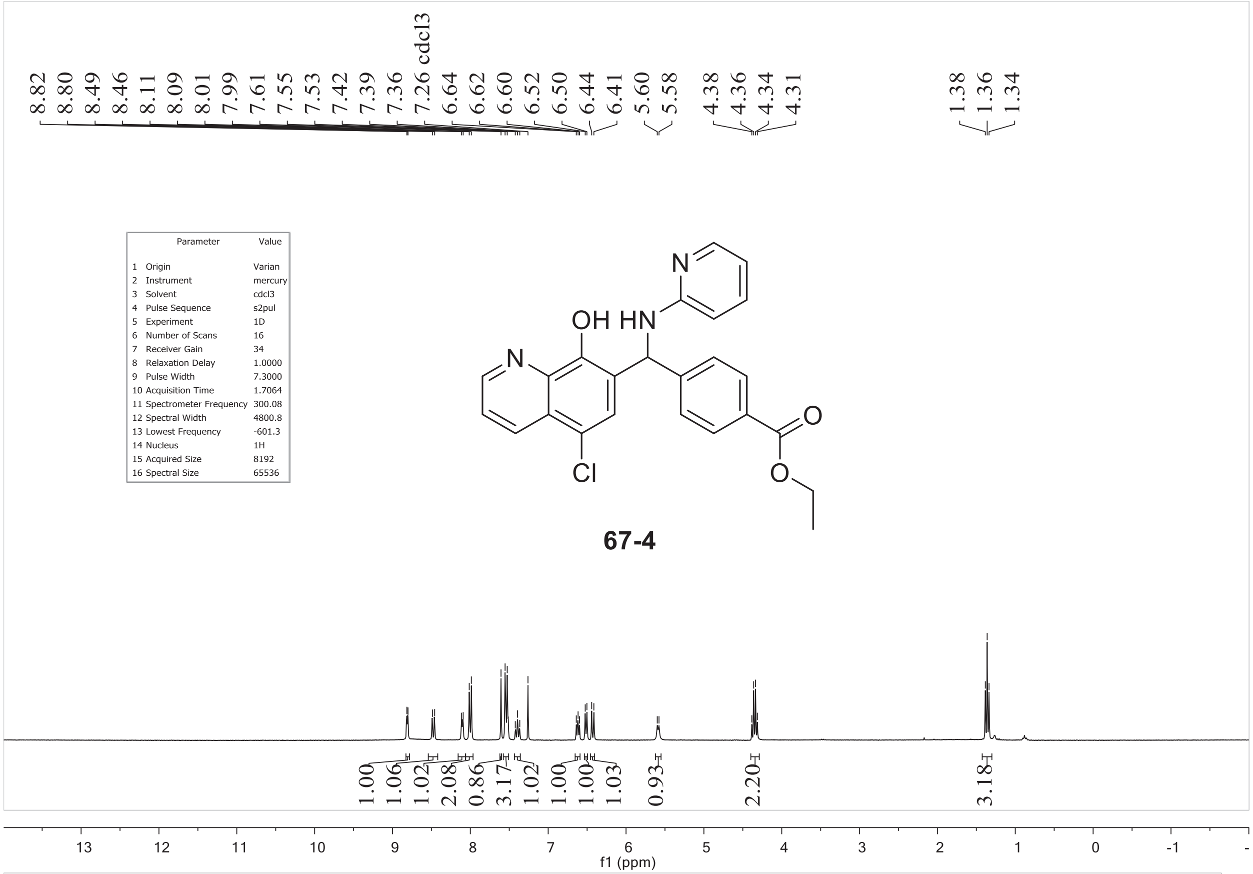
8.82 8.80 8.49 8.46 8.11 8.09 8.01 7.99 7.61 7.55 7.53 7.42 7.39 7.36 7.26 cdcl3 6.64 6.62 6.60 6.52 6.50 6.44 6.41 5.60 5.58 4.38 4.36 4.34 4.31 1.38 1.36 1.34
Parameter Value
1 Origin Varian
2 Instrument mercury
3 Solvent cdcl3
4 Pulse Sequence s2pul
5 Experiment 1D
6 Number of Scans 16
7 Receiver Gain 34
8 Relaxation Delay 1.0000
9 Pulse Width 7.3000
10 Acquisition Time 1.7064
11 Spectrometer Frequency 300.08
12 Spectral Width 4800.8
13 Lowest Frequency -601.3
14 Nucleus 1H
15 Acquired Size 8192
16 Spectral Size 65536
OH HN N N Cl O O
67-4
1.00 1.06 1.02 2.08 0.86 3.17 1.02 1.00 1.00 1.03 0.93 2.20 3.18
13 12 11 10 9 8 7 6 5 4 3 2 1 0 -1
f1 (ppm)

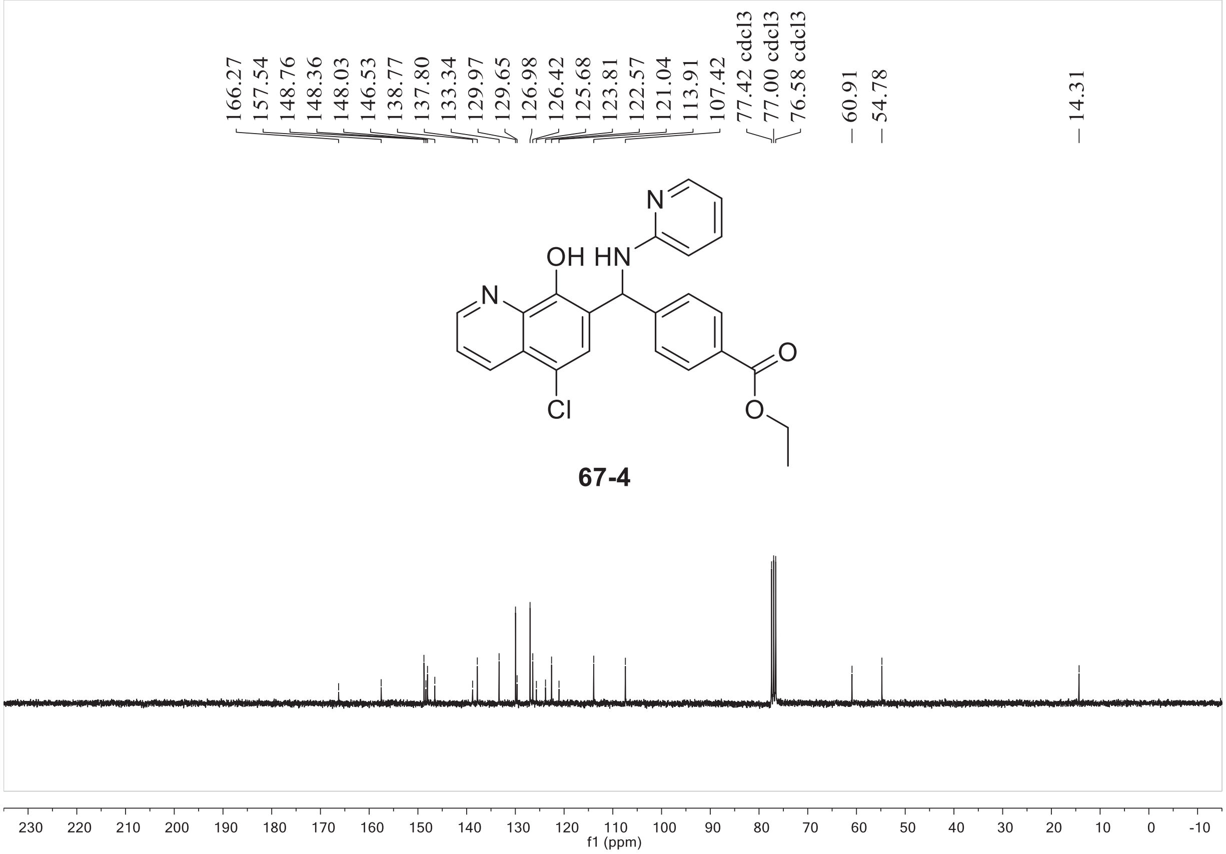
166.27 157.54 148.76 148.36 148.03 146.53 138.77 137.80 133.34 129.97 129.65 126.98 126.42 125.68 123.81 122.57 121.04 113.91 107.42 77.42 cdcl3 77.00 cdcl3 76.58 cdcl3 60.91 54.78 14.31
OH HN N N Cl O O
67-4
230 220 210 200 190 180 170 160 150 140 130 120 110 100 90 80 70 60 50 40 30 20 10 0 -10
f1 (ppm)

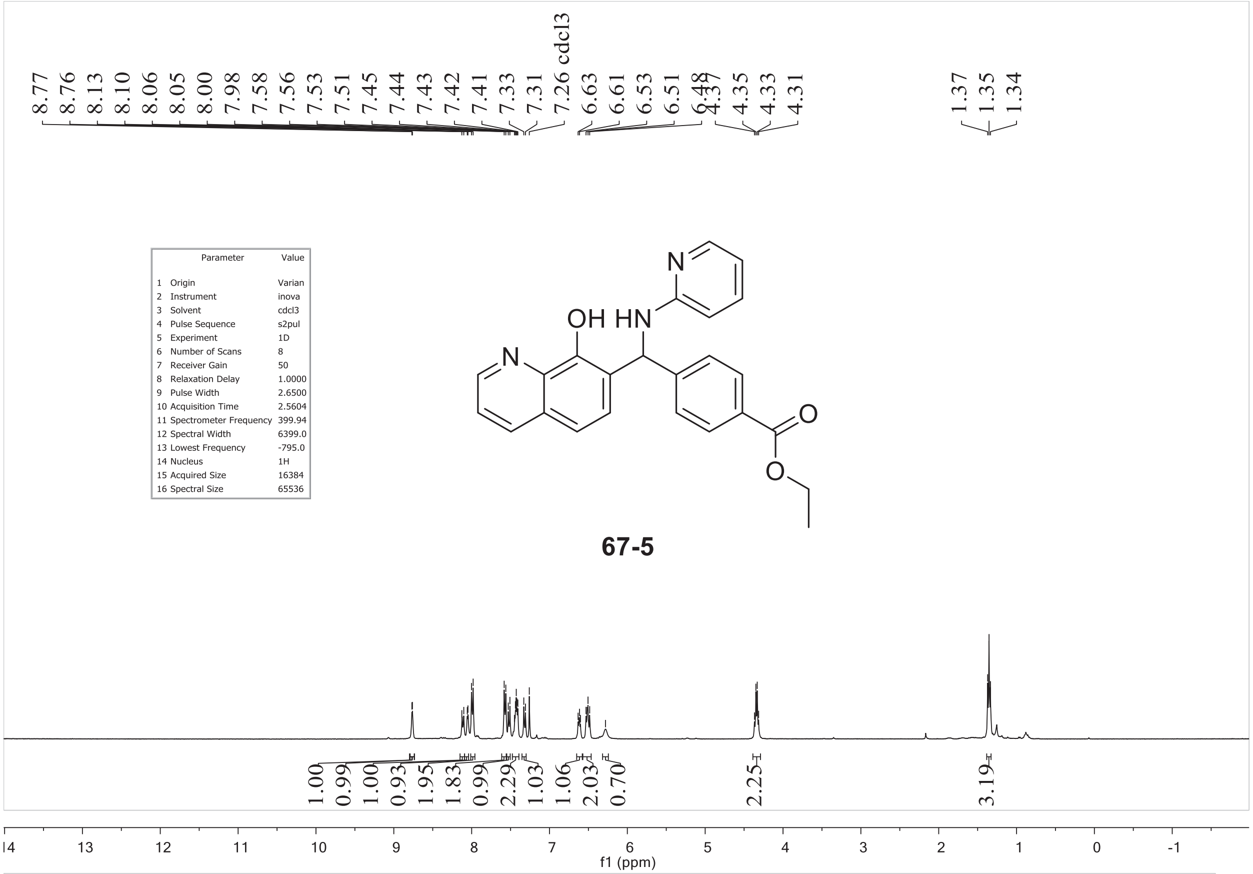
8.77 8.76 8.13 8.10 8.06 8.05 8.00 7.98 7.58 7.56 7.53 7.51 7.45 7.44 7.43 7.42 7.41 7.33 7.31 7.26 cdcl3 6.63 6.61 6.53 6.51 6.48 4.37 4.35 4.33 4.31 1.37 1.35 1.34
Parameter Value
1 Origin Varian
2 Instrument inova
3 Solvent cdcl3
4 Pulse Sequence s2pul
5 Experiment 1D
6 Number of Scans 8
7 Receiver Gain 50
8 Relaxation Delay 1.0000
9 Pulse Width 2.6500
10 Acquisition Time 2.5604
11 Spectrometer Frequency 399.94
12 Spectral Width 6399.0
13 Lowest Frequency -795.0
14 Nucleus 1H
15 Acquired Size 16384
16 Spectral Size 65536
OH HN
N
N
O
O
67-5
1.00 0.99 1.00 0.93 1.95 1.83 0.99 2.29 1.03 1.06 2.03 0.70 2.25 3.19
14 13 12 11 10 9 8 7 6 5 4 3 2 1 0 -1
f1 (ppm)

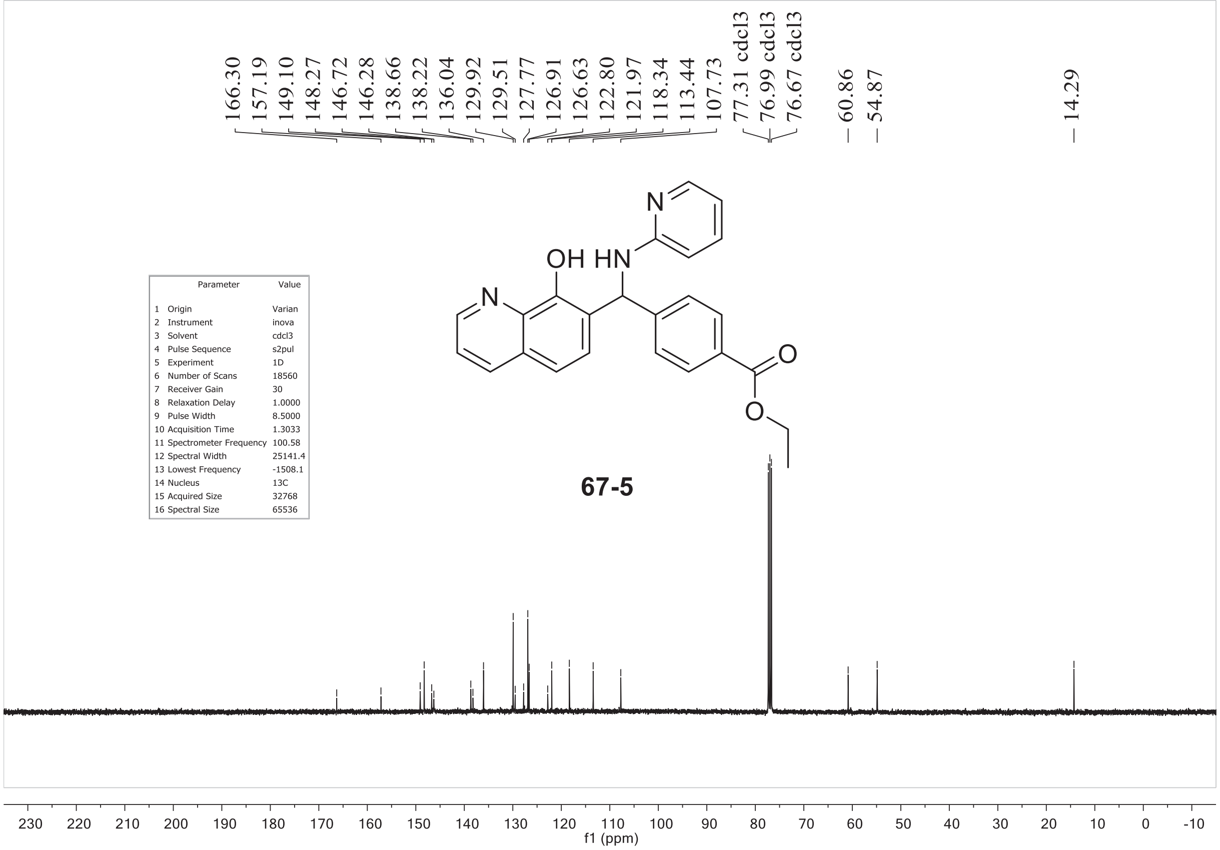
166.30 157.19 149.10 148.27 146.72 146.28 138.66 138.22 136.04 129.92 129.51 127.77 126.91 126.63 122.80 121.97 118.34 113.44 107.73 77.31 cdcl3 76.99 cdcl3 76.67 cdcl3 60.86 54.87 14.29
Parameter Value
1 Origin Varian
2 Instrument inova
3 Solvent cdcl3
4 Pulse Sequence s2pul
5 Experiment 1D
6 Number of Scans 18560
7 Receiver Gain 30
8 Relaxation Delay 1.0000
9 Pulse Width 8.5000
10 Acquisition Time 1.3033
11 Spectrometer Frequency 100.58
12 Spectral Width 25141.4
13 Lowest Frequency -1508.1
14 Nucleus 13C
15 Acquired Size 32768
16 Spectral Size 65536
OH HN
N
N
O
O
67-5
230 220 210 200 190 180 170 160 150 140 130 120 110 100 90 80 70 60 50 40 30 20 10 0 -10
f1 (ppm)

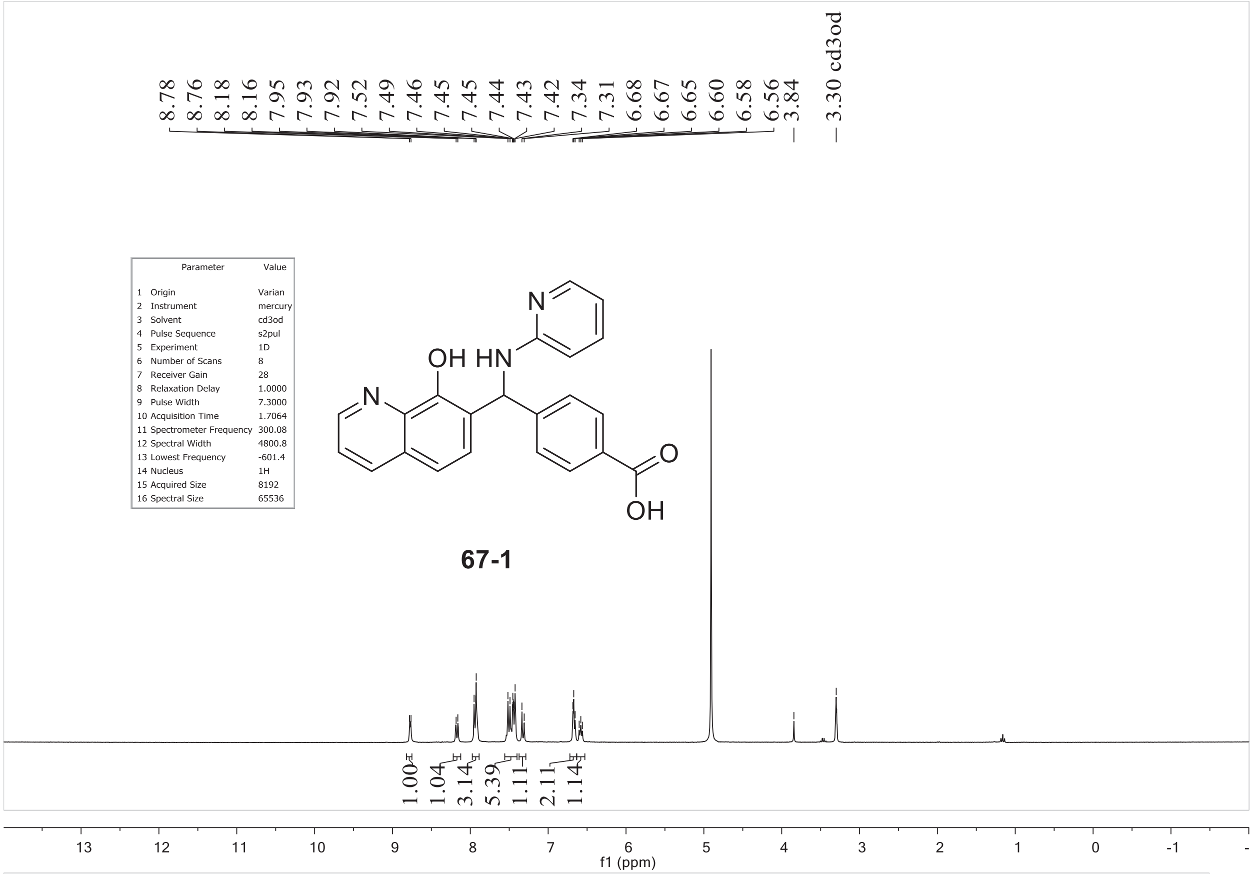
8.78 8.76 8.18 8.16 7.95 7.93 7.92 7.52 7.49 7.46 7.45 7.45 7.44 7.43 7.42 7.34 7.31 6.68 6.67 6.65 6.60 6.58 6.56 3.84
3.30 cd3od
Parameter Value
1 Origin Varian
2 Instrument mercury
3 Solvent cd3od
4 Pulse Sequence s2pul
5 Experiment 1D
6 Number of Scans 8
7 Receiver Gain 28
8 Relaxation Delay 1.0000
9 Pulse Width 7.3000
10 Acquisition Time 1.7064
11 Spectrometer Frequency 300.08
12 Spectral Width 4800.8
13 Lowest Frequency -601.4
14 Nucleus 1H
15 Acquired Size 8192
16 Spectral Size 65536
OH HN
N
N
O
OH
67-1
1.00 1.04 3.14 5.39 1.11 2.11 1.14
13 12 11 10 9 8 7 6 5 4 3 2 1 0 -1
f1 (ppm)

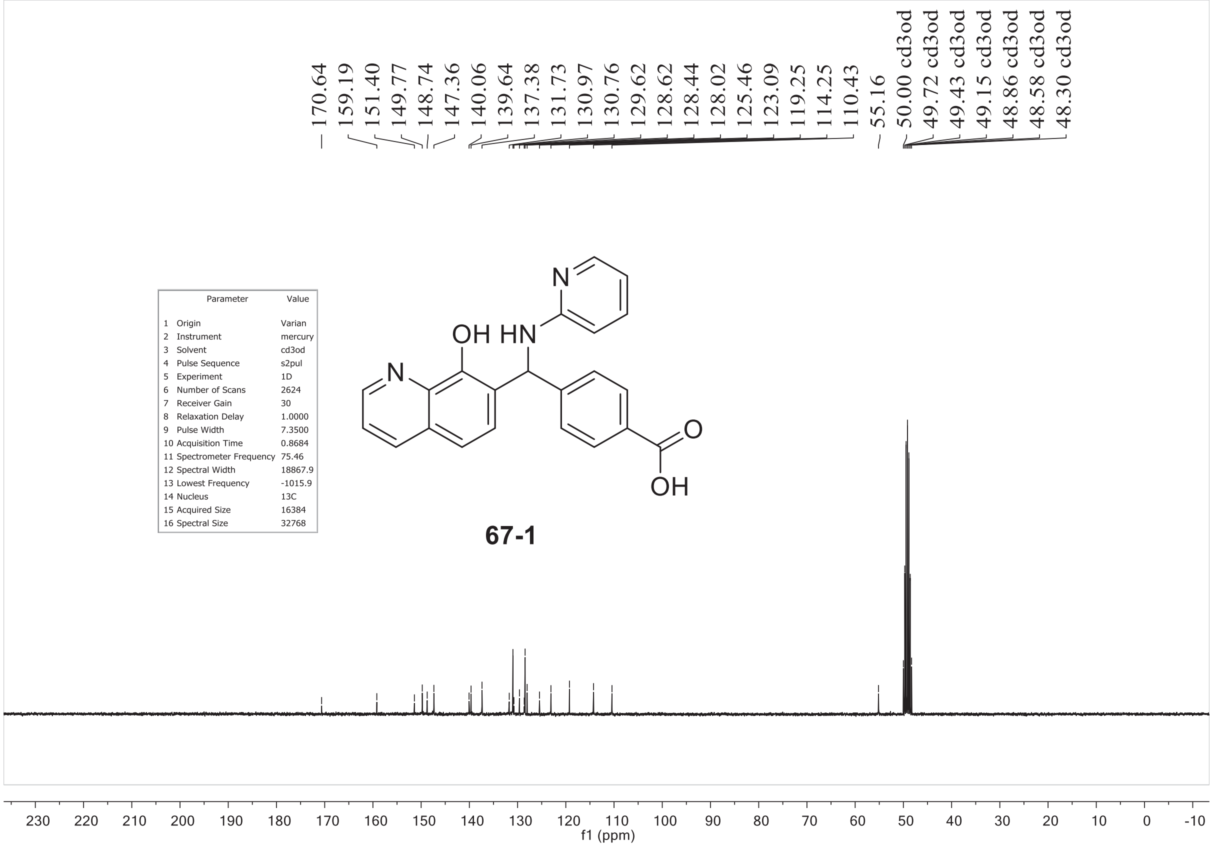
170.64 159.19 151.40 149.77 148.74 147.36 140.06 139.64 137.38 131.73 130.97 130.76 129.62 128.62 128.44 128.02 125.46 123.09 119.25 114.25 110.43 55.16
50.00 cd3od 49.72 cd3od 49.43 cd3od 49.15 cd3od 48.86 cd3od 48.58 cd3od 48.30 cd3od
Parameter Value
1 Origin Varian
2 Instrument mercury
3 Solvent cd3od
4 Pulse Sequence s2pul
5 Experiment 1D
6 Number of Scans 2624
7 Receiver Gain 30
8 Relaxation Delay 1.0000
9 Pulse Width 7.3500
10 Acquisition Time 0.8684
11 Spectrometer Frequency 75.46
12 Spectral Width 18867.9
13 Lowest Frequency -1015.9
14 Nucleus 13C
15 Acquired Size 16384
16 Spectral Size 32768
OH HN
N
N
O
OH
67-1
230 220 210 200 190 180 170 160 150 140 130 120 110 100 90 80 70 60 50 40 30 20 10 0 -10
f1 (ppm)

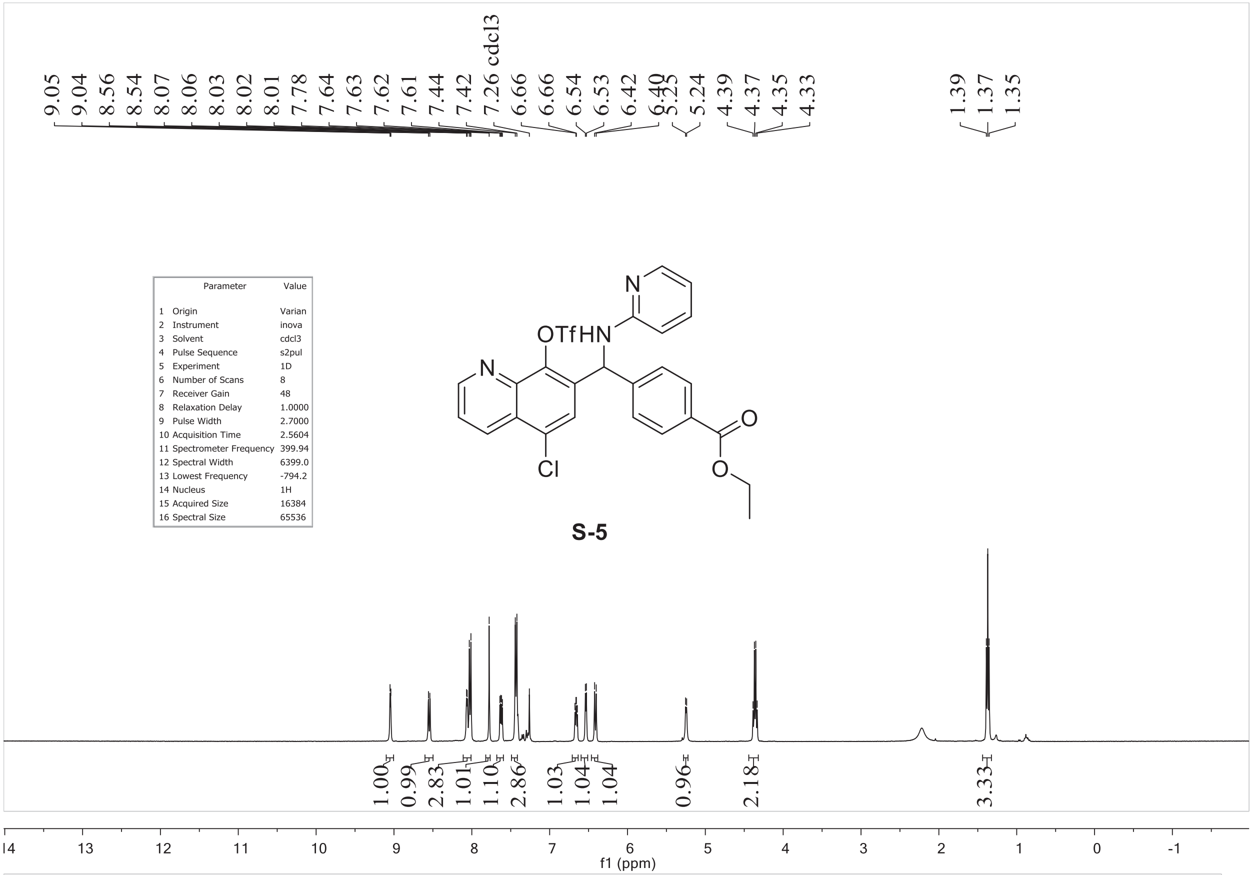
9.05 9.04 8.56 8.54 8.07 8.06 8.03 8.02 8.01 7.78 7.64 7.63 7.62 7.61 7.44 7.42 7.26 cdcl3 6.66 6.66 6.54 6.53 6.42 6.40 5.25 5.24 4.39 4.37 4.35 4.33 1.39 1.37 1.35
Parameter Value
1 Origin Varian
2 Instrument inova
3 Solvent cdcl3
4 Pulse Sequence s2pul
5 Experiment 1D
6 Number of Scans 8
7 Receiver Gain 48
8 Relaxation Delay 1.0000
9 Pulse Width 2.7000
10 Acquisition Time 2.5604
11 Spectrometer Frequency 399.94
12 Spectral Width 6399.0
13 Lowest Frequency -794.2
14 Nucleus 1H
15 Acquired Size 16384
16 Spectral Size 65536
OTf HN
N
N
Cl
O
O
S-5
1.00 0.99 2.83 1.01 1.10 2.86 1.03 1.04 1.04 0.96 2.18 3.33
14 13 12 11 10 9 8 7 6 5 4 3 2 1 0 -1
f1 (ppm)

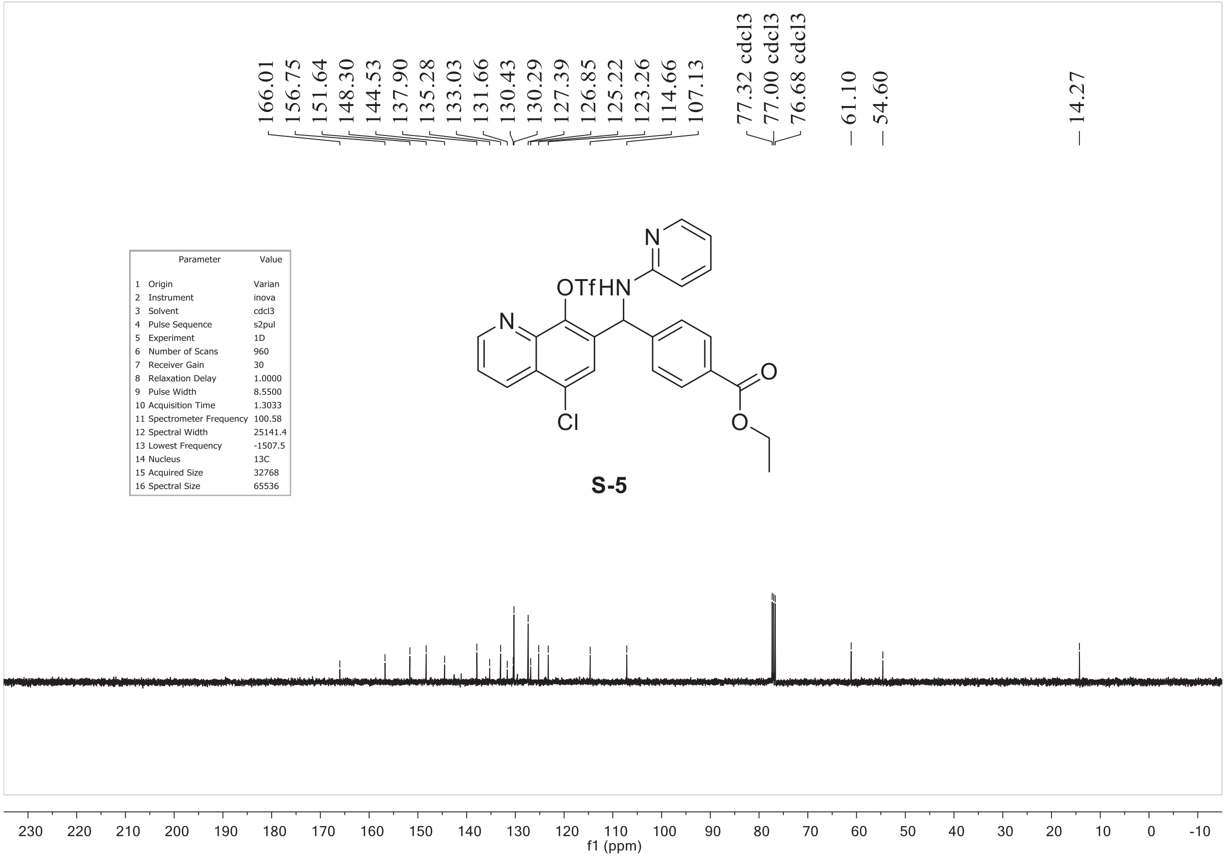
166.01 156.75 151.64 148.30 144.53 137.90 135.28 133.03 131.66 130.43 130.29 127.39 126.85 125.22 123.26 114.66 107.13 77.32 cdcl3 77.00 cdcl3 76.68 cdcl3 61.10 54.60 14.27
Parameter Value
1 Origin Varian
2 Instrument inova
3 Solvent cdcl3
4 Pulse Sequence s2pul
5 Experiment 1D
6 Number of Scans 960
7 Receiver Gain 30
8 Relaxation Delay 1.0000
9 Pulse Width 8.5500
10 Acquisition Time 1.3033
11 Spectrometer Frequency 100.58
12 Spectral Width 25141.4
13 Lowest Frequency -1507.5
14 Nucleus 13C
15 Acquired Size 32768
16 Spectral Size 65536
OTf HN
N
N
Cl
O
O
S-5
230 220 210 200 190 180 170 160 150 140 130 120 110 100 90 80 70 60 50 40 30 20 10 0 -10
f1 (ppm)

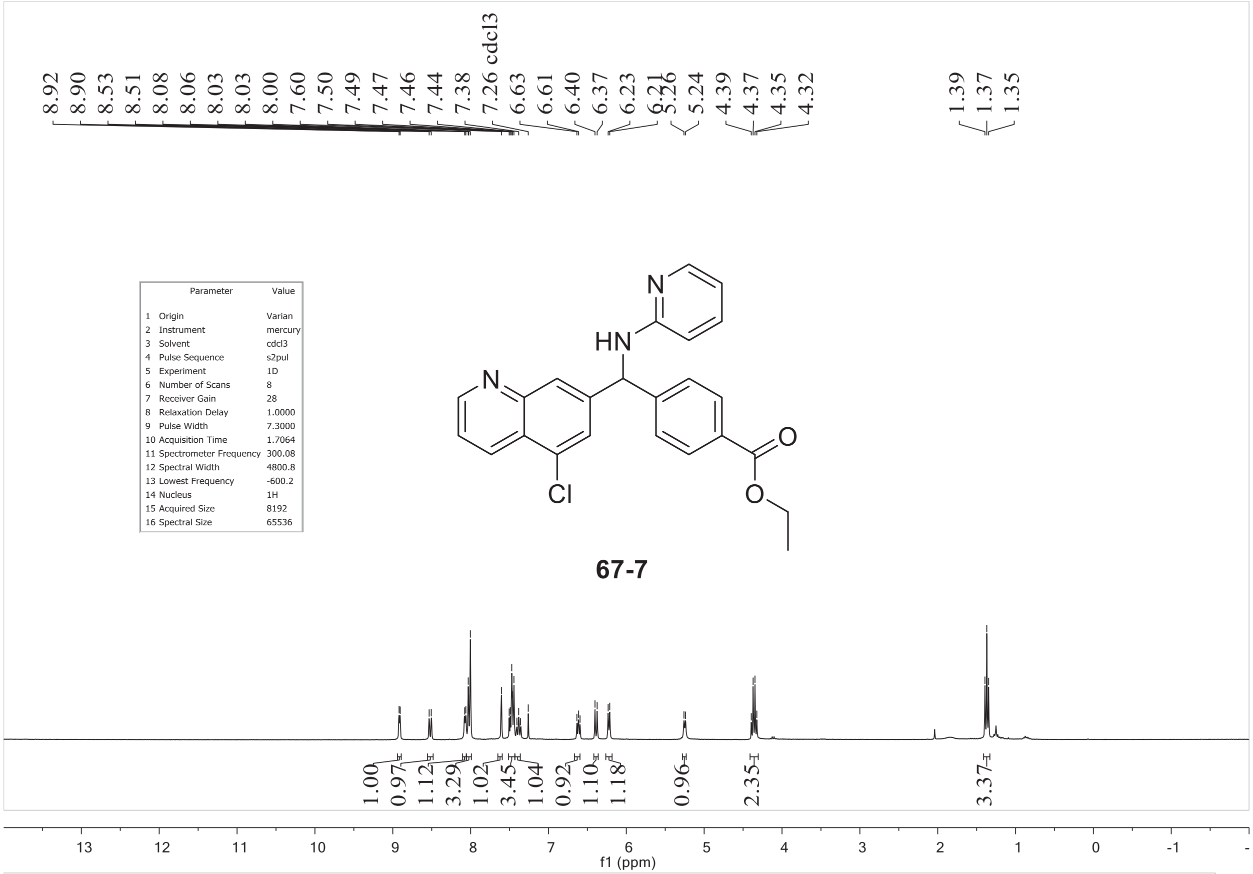
8.92
8.90
8.53
8.51
8.08
8.06
8.03
8.03
8.00
7.60
7.50
7.49
7.47
7.46
7.44
7.38
7.26 cdcl3
6.63
6.61
6.40
6.37
6.23
6.21
5.26
5.24
4.39
4.37
4.35
4.32
1.39
1.37
1.35
Parameter Value
1 Origin Varian
2 Instrument mercury
3 Solvent cdcl3
4 Pulse Sequence s2pul
5 Experiment 1D
6 Number of Scans 8
7 Receiver Gain 28
8 Relaxation Delay 1.0000
9 Pulse Width 7.3000
10 Acquisition Time 1.7064
11 Spectrometer Frequency 300.08
12 Spectral Width 4800.8
13 Lowest Frequency -600.2
14 Nucleus 1H
15 Acquired Size 8192
16 Spectral Size 65536
N
HN
N
O
O
Cl
67-7
1.00
0.97
1.12
3.29
1.02
3.45
1.04
0.92
1.10
1.18
0.96
2.35
3.37
13 12 11 10 9 8 7 6 5 4 3 2 1 0 -1
f1 (ppm)

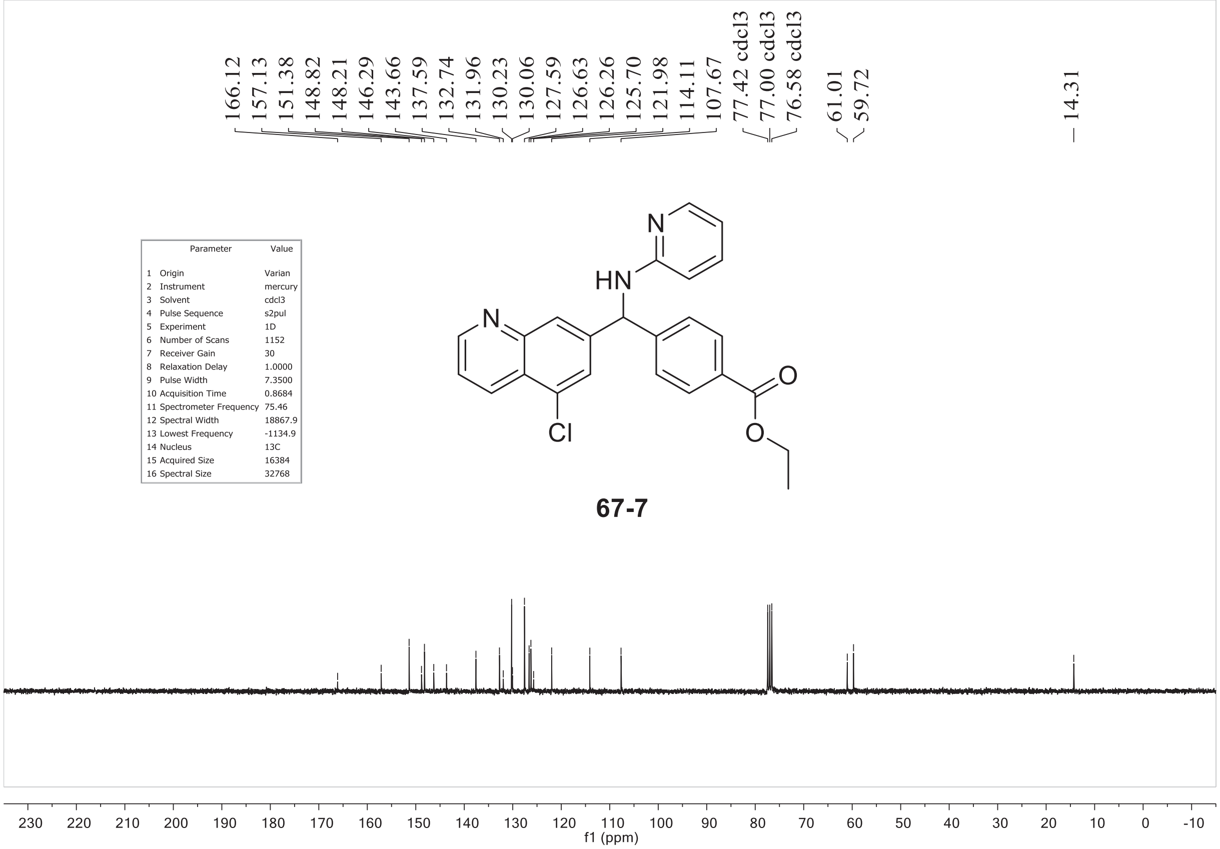
166.12
157.13
151.38
148.82
148.21
146.29
143.66
137.59
132.74
131.96
130.23
130.06
127.59
126.63
126.26
125.70
121.98
114.11
107.67
77.42 cdcl3
77.00 cdcl3
76.58 cdcl3
61.01
59.72
14.31
Parameter Value
1 Origin Varian
2 Instrument mercury
3 Solvent cdcl3
4 Pulse Sequence s2pul
5 Experiment 1D
6 Number of Scans 1152
7 Receiver Gain 30
8 Relaxation Delay 1.0000
9 Pulse Width 7.3500
10 Acquisition Time 0.8684
11 Spectrometer Frequency 75.46
12 Spectral Width 18867.9
13 Lowest Frequency -1134.9
14 Nucleus 13C
15 Acquired Size 16384
16 Spectral Size 32768
N
HN
N
O
O
Cl
67-7
230 220 210 200 190 180 170 160 150 140 130 120 110 100 90 80 70 60 50 40 30 20 10 0 -10
f1 (ppm)

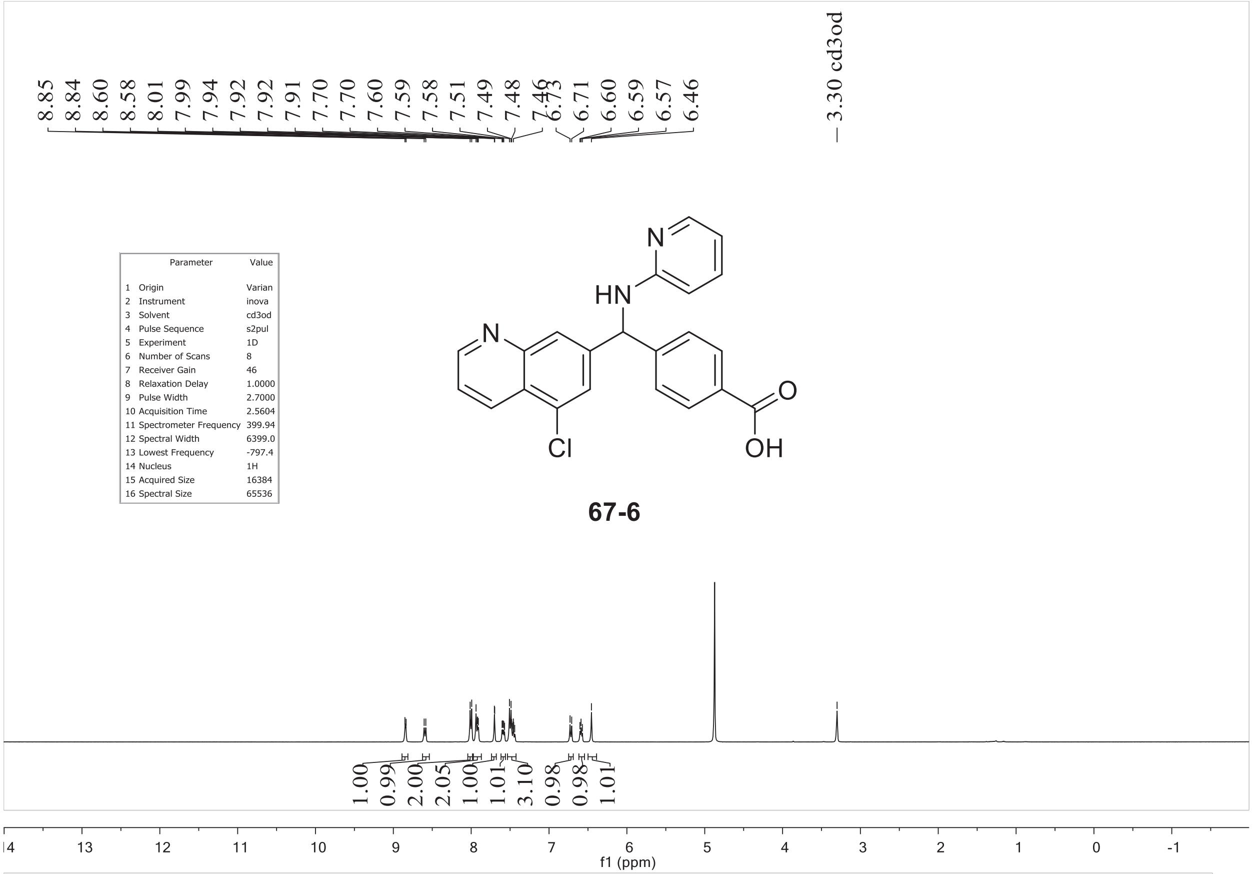
8.85 8.84 8.60 8.58 8.01 7.99 7.94 7.92 7.92 7.91 7.70 7.70 7.60 7.59 7.58 7.51 7.49 7.48 7.46 6.73 6.71 6.60 6.59 6.57 6.46
3.30 cd3od
Parameter Value
1 Origin Varian
2 Instrument inova
3 Solvent cd3od
4 Pulse Sequence s2pul
5 Experiment 1D
6 Number of Scans 8
7 Receiver Gain 46
8 Relaxation Delay 1.0000
9 Pulse Width 2.7000
10 Acquisition Time 2.5604
11 Spectrometer Frequency 399.94
12 Spectral Width 6399.0
13 Lowest Frequency -797.4
14 Nucleus 1H
15 Acquired Size 16384
16 Spectral Size 65536
N
HN
N
Cl
O
OH
67-6
1.00 0.99 2.00 2.05 1.00 1.01 3.10 0.98 0.98 1.01
14 13 12 11 10 9 8 7 6 5 4 3 2 1 0 -1
f1 (ppm)

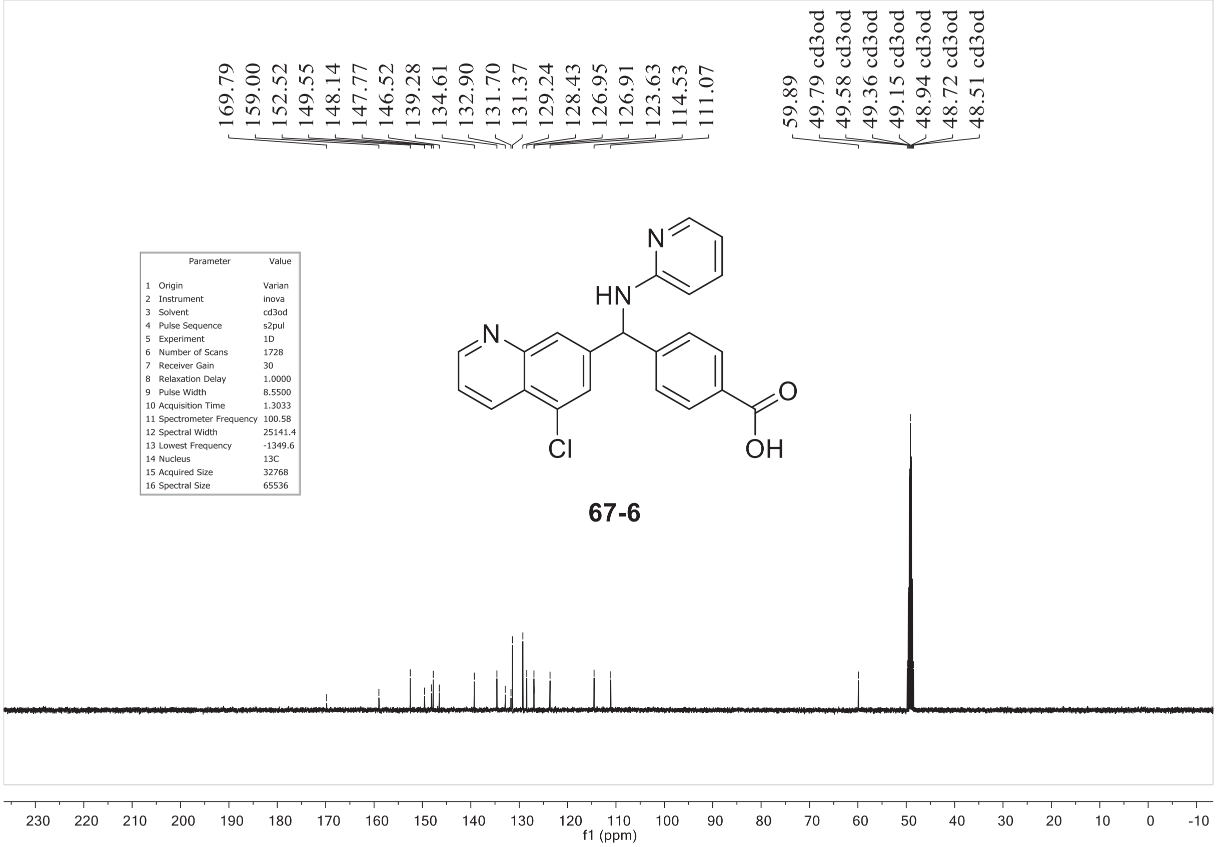
169.79 159.00 152.52 149.55 148.14 147.77 146.52 139.28 134.61 132.90 131.70 131.37 129.24 128.43 126.95 126.91 123.63 114.53 111.07
59.89 49.79 cd3od 49.58 cd3od 49.36 cd3od 49.15 cd3od 48.94 cd3od 48.72 cd3od 48.51 cd3od
Parameter Value
1 Origin Varian
2 Instrument inova
3 Solvent cd3od
4 Pulse Sequence s2pul
5 Experiment 1D
6 Number of Scans 1728
7 Receiver Gain 30
8 Relaxation Delay 1.0000
9 Pulse Width 8.5500
10 Acquisition Time 1.3033
11 Spectrometer Frequency 100.58
12 Spectral Width 25141.4
13 Lowest Frequency -1349.6
14 Nucleus 13C
15 Acquired Size 32768
16 Spectral Size 65536
N
HN
N
Cl
O
OH
67-6
230 220 210 200 190 180 170 160 150 140 130 120 110 100 90 80 70 60 50 40 30 20 10 0 -10
f1 (ppm)

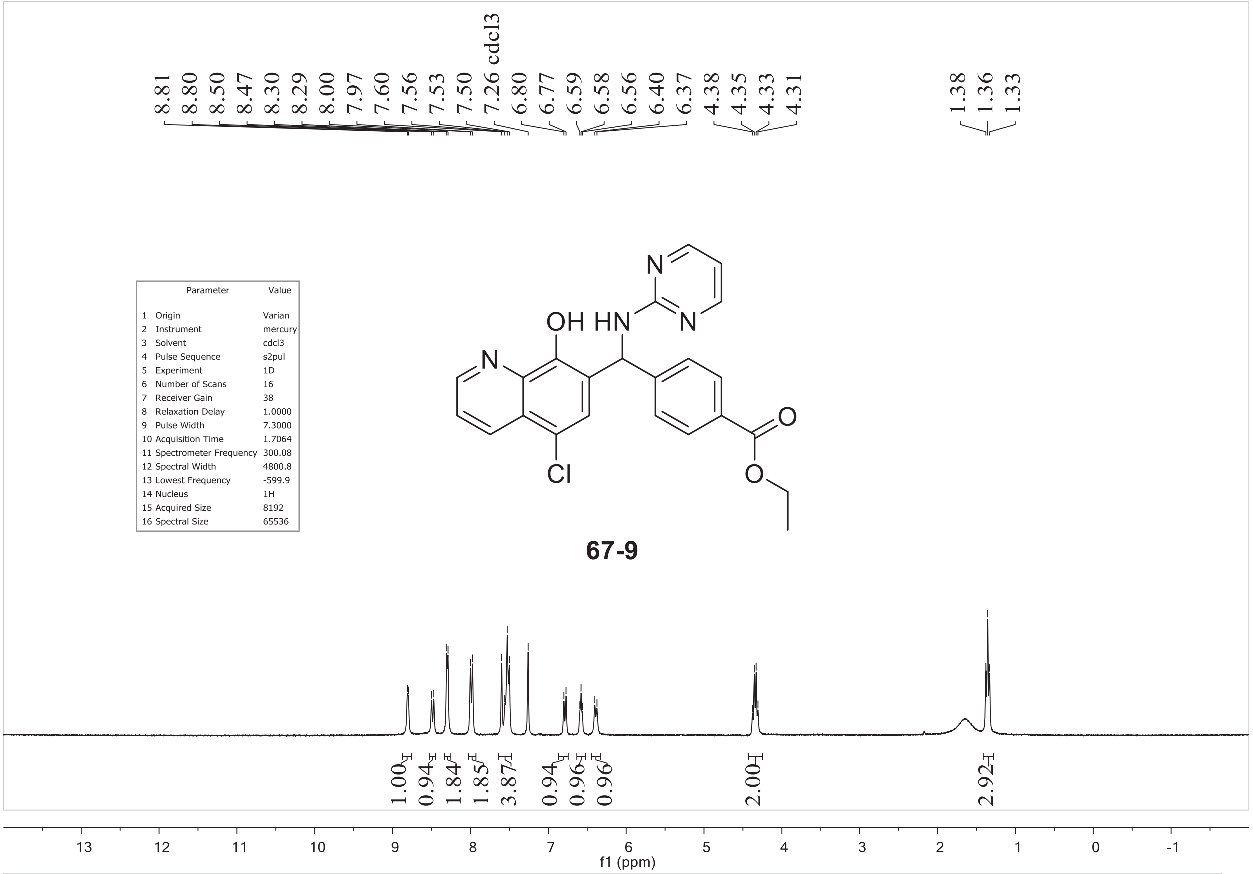

8.81 8.80 8.50 8.47 8.30 8.29 8.00 7.97 7.60 7.56 7.53 7.50 7.26 cdcl3 6.80 6.77 6.59 6.58 6.56 6.40 6.37 4.38 4.35 4.33 4.31 1.38 1.36 1.33
Parameter Value
1 Origin Varian
2 Instrument mercury
3 Solvent cdcl3
4 Pulse Sequence s2pul
5 Experiment 1D
6 Number of Scans 16
7 Receiver Gain 38
8 Relaxation Delay 1.0000
9 Pulse Width 7.3000
10 Acquisition Time 1.7064
11 Spectrometer Frequency 300.08
12 Spectral Width 4800.8
13 Lowest Frequency -599.9
14 Nucleus 1H
15 Acquired Size 8192
16 Spectral Size 65536
OH HN N N N Cl O O
67-9
1.00 0.94 1.84 1.85 3.87 0.94 0.96 0.96 2.00 2.92
13 12 11 10 9 8 7 6 5 4 3 2 1 0 -1
f1 (ppm)

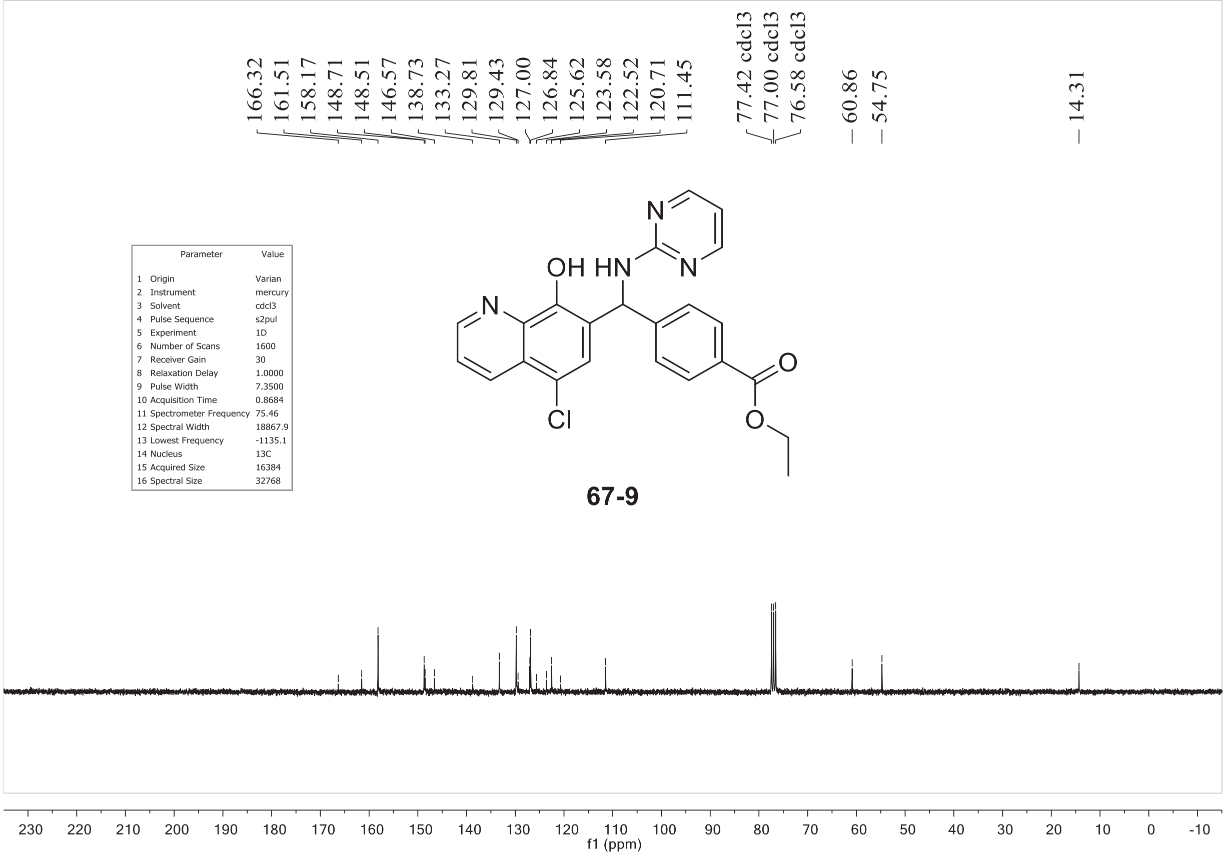

166.32 161.51 158.17 148.71 148.51 146.57 138.73 133.27 129.81 129.43 127.00 126.84 125.62 123.58 122.52 120.71 111.45 77.42 cdcl3 77.00 cdcl3 76.58 cdcl3 60.86 54.75 14.31
Parameter Value
1 Origin Varian
2 Instrument mercury
3 Solvent cdcl3
4 Pulse Sequence s2pul
5 Experiment 1D
6 Number of Scans 1600
7 Receiver Gain 30
8 Relaxation Delay 1.0000
9 Pulse Width 7.3500
10 Acquisition Time 0.8684
11 Spectrometer Frequency 75.46
12 Spectral Width 18867.9
13 Lowest Frequency -1135.1
14 Nucleus 13C
15 Acquired Size 16384
16 Spectral Size 32768
OH HN N N N Cl O O
67-9
230 220 210 200 190 180 170 160 150 140 130 120 110 100 90 80 70 60 50 40 30 20 10 0 -10
f1 (ppm)

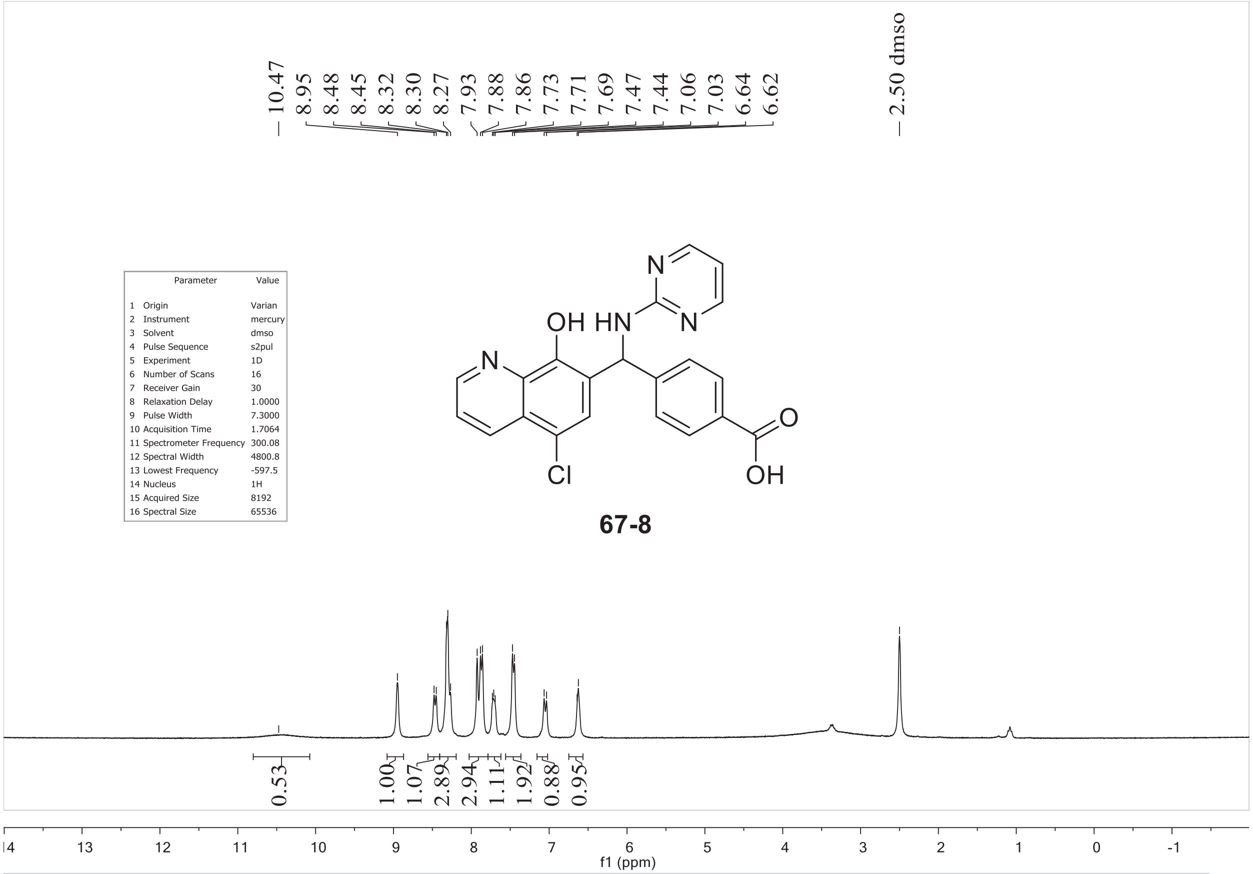
10.47
8.95
8.48
8.45
8.32
8.30
8.27
7.93
7.88
7.86
7.73
7.71
7.69
7.47
7.44
7.06
7.03
6.64
6.62
2.50 dmso
Parameter Value
1 Origin Varian
2 Instrument mercury
3 Solvent dmso
4 Pulse Sequence s2pul
5 Experiment 1D
6 Number of Scans 16
7 Receiver Gain 30
8 Relaxation Delay 1.0000
9 Pulse Width 7.3000
10 Acquisition Time 1.7064
11 Spectrometer Frequency 300.08
12 Spectral Width 4800.8
13 Lowest Frequency -597.5
14 Nucleus 1H
15 Acquired Size 8192
16 Spectral Size 65536
OH HN N N N Cl O OH
67-8
0.53
1.00
1.07
2.89
2.94
1.11
1.92
0.88
0.95
14 13 12 11 10 9 8 7 6 5 4 3 2 1 0 -1
f1 (ppm)

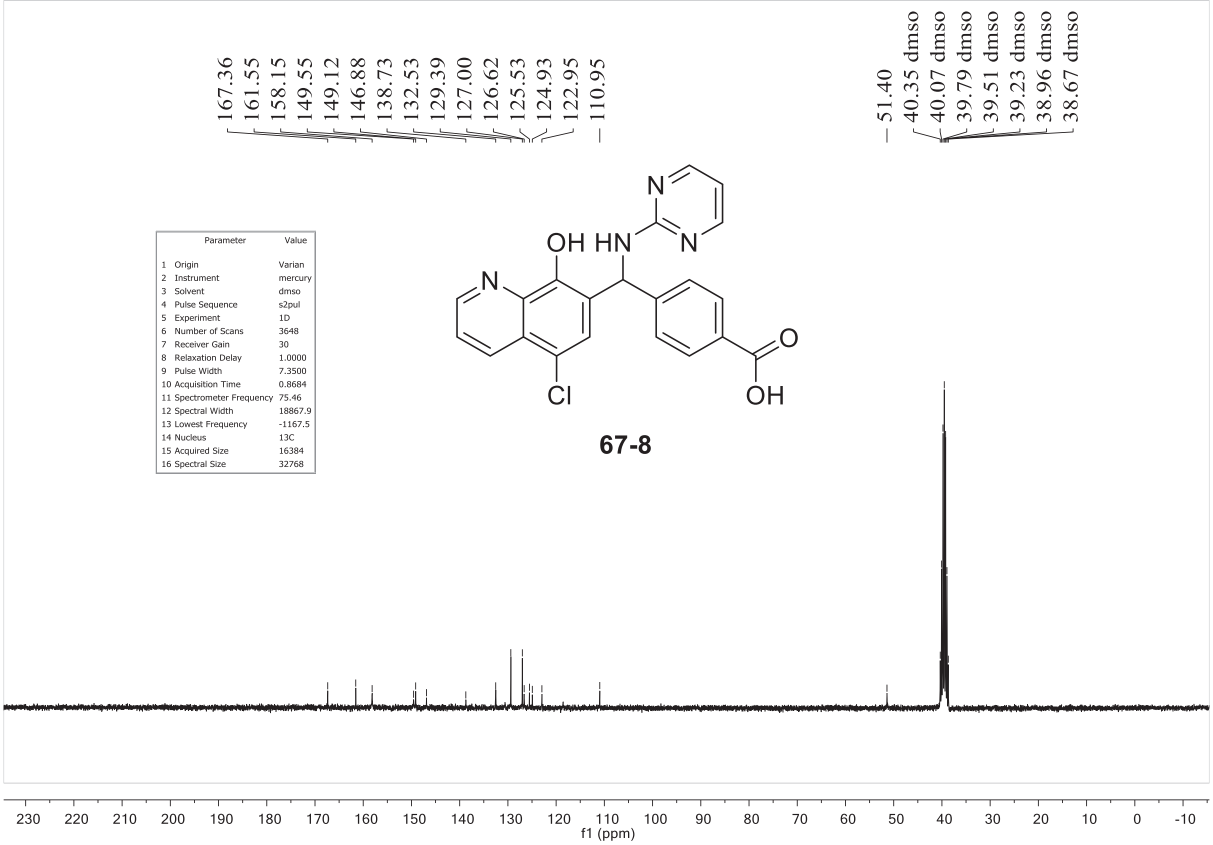
167.36
161.55
158.15
149.55
149.12
146.88
138.73
132.53
129.39
127.00
126.62
125.53
124.93
122.95
110.95
51.40
40.35 dmso
40.07 dmso
39.79 dmso
39.51 dmso
39.23 dmso
38.96 dmso
38.67 dmso
Parameter Value
1 Origin Varian
2 Instrument mercury
3 Solvent dmso
4 Pulse Sequence s2pul
5 Experiment 1D
6 Number of Scans 3648
7 Receiver Gain 30
8 Relaxation Delay 1.0000
9 Pulse Width 7.3500
10 Acquisition Time 0.8684
11 Spectrometer Frequency 75.46
12 Spectral Width 18867.9
13 Lowest Frequency -1167.5
14 Nucleus 13C
15 Acquired Size 16384
16 Spectral Size 32768
OH HN N N N Cl O OH
67-8
230 220 210 200 190 180 170 160 150 140 130 120 110 100 90 80 70 60 50 40 30 20 10 0 -10
f1 (ppm)

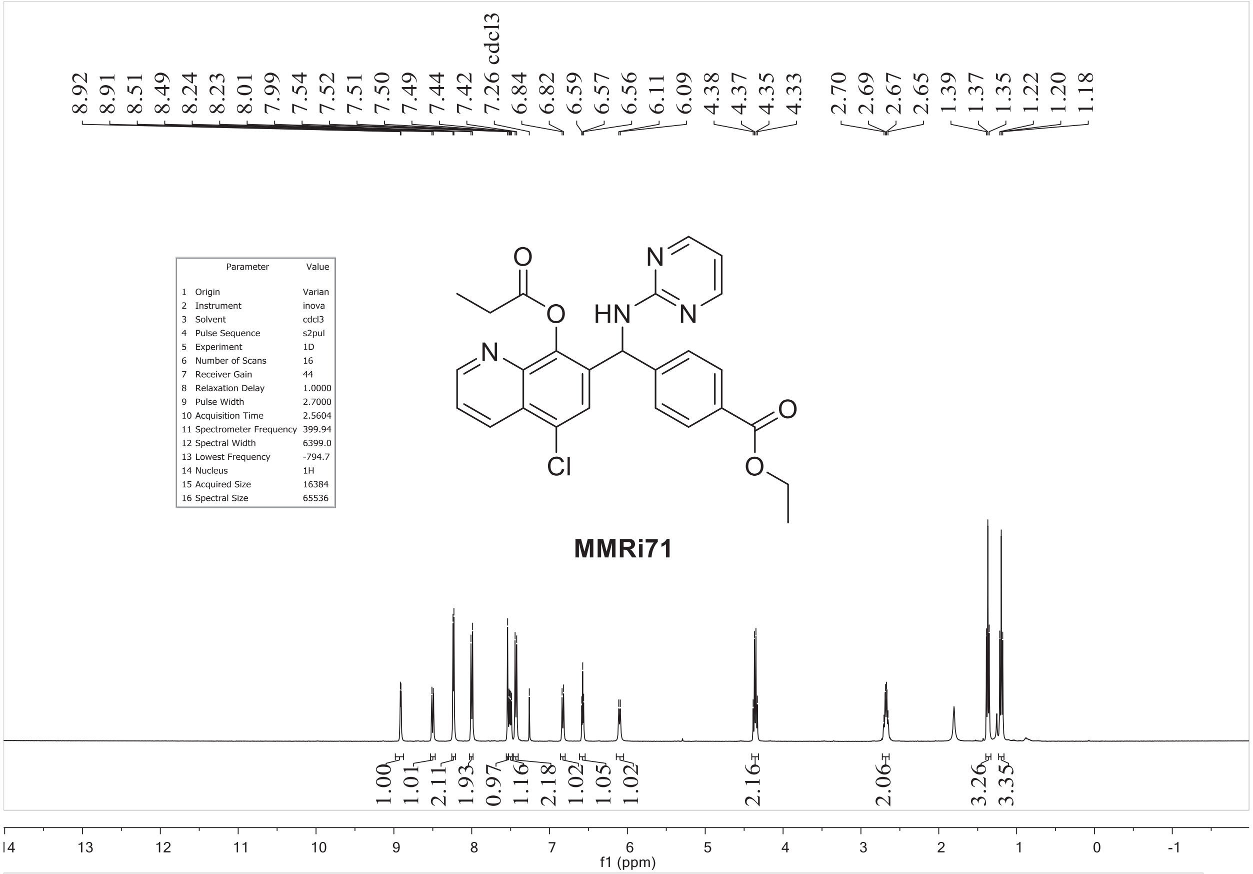
8.92 8.91 8.51 8.49 8.24 8.23 8.01 7.99 7.54 7.52 7.51 7.50 7.49 7.44 7.42 7.26 cdcl3 6.84 6.82 6.59 6.57 6.56 6.11 6.09 4.38 4.37 4.35 4.33 2.70 2.69 2.67 2.65 1.39 1.37 1.35 1.22 1.20 1.18
Parameter Value
1 Origin Varian
2 Instrument inova
3 Solvent cdcl3
4 Pulse Sequence s2pul
5 Experiment 1D
6 Number of Scans 16
7 Receiver Gain 44
8 Relaxation Delay 1.0000
9 Pulse Width 2.7000
10 Acquisition Time 2.5604
11 Spectrometer Frequency 399.94
12 Spectral Width 6399.0
13 Lowest Frequency -794.7
14 Nucleus 1H
15 Acquired Size 16384
16 Spectral Size 65536
O
O
HN
N
N
N
Cl
O
O
MMRi71
1.00 1.01 2.11 1.93 0.97 1.16 2.18 1.02 1.05 1.02 2.16 2.06 3.26 3.35
14 13 12 11 10 9 8 7 6 5 4 3 2 1 0 -1
f1 (ppm)

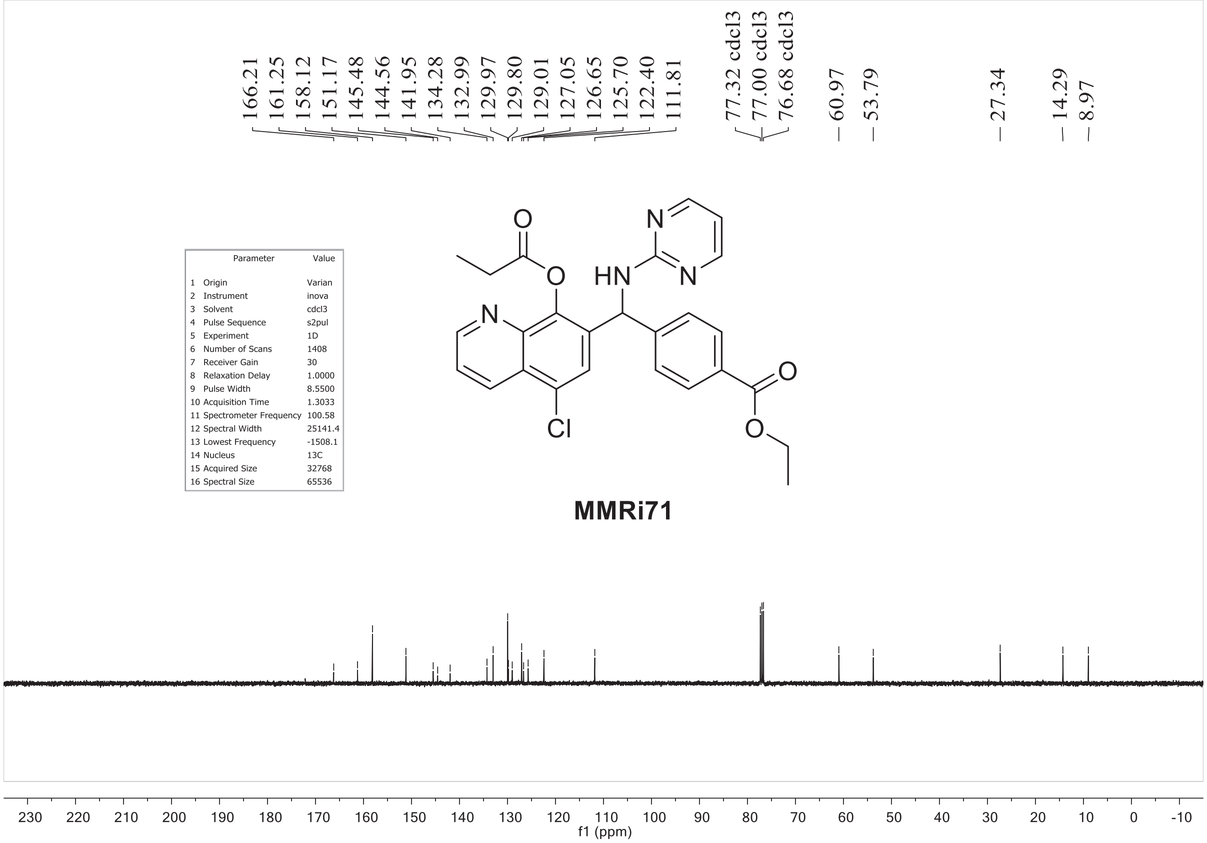
166.21 161.25 158.12 151.17 145.48 144.56 141.95 134.28 132.99 129.97 129.80 129.01 127.05 126.65 125.70 122.40 111.81 77.32 cdcl3 77.00 cdcl3 76.68 cdcl3 60.97 53.79 27.34 14.29 8.97
Parameter Value
1 Origin Varian
2 Instrument inova
3 Solvent cdcl3
4 Pulse Sequence s2pul
5 Experiment 1D
6 Number of Scans 1408
7 Receiver Gain 30
8 Relaxation Delay 1.0000
9 Pulse Width 8.5500
10 Acquisition Time 1.3033
11 Spectrometer Frequency 100.58
12 Spectral Width 25141.4
13 Lowest Frequency -1508.1
14 Nucleus 13C
15 Acquired Size 32768
16 Spectral Size 65536
O
O
HN
N
N
N
Cl
O
O
MMRi71
230 220 210 200 190 180 170 160 150 140 130 120 110 100 90 80 70 60 50 40 30 20 10 0 -10
f1 (ppm)
